# Folding molecular origami from ribosomal RNA

**DOI:** 10.1186/s12951-024-02489-2

**Published:** 2024-05-02

**Authors:** Anastasia Shapiro, Noah Joseph, Nadav Mellul, Almogit Abu-Horowitz, Boaz Mizrahi, Ido Bachelet

**Affiliations:** 1 Augmanity Nano Ltd., 8 Hamada St., 7670308 Rehovot, Israel; 2 Technion, Faculty of Biotechnology and Food Engineering, 32000 Haifa, Israel

**Keywords:** RNA origami, RNA/DNA nanotechnology, Nanostructures, Molecular origami stability, Polyethylene glycol (PEG) coating, Hybrid origami structures

## Abstract

Approximately 80 percent of the total RNA in cells is ribosomal RNA (rRNA), making it an abundant and inexpensive natural source of long, single-stranded nucleic acid, which could be used as raw material for the fabrication of molecular origami. In this study, we demonstrate efficient and robust construction of 2D and 3D origami nanostructures utilizing cellular rRNA as a scaffold and DNA oligonucleotide staples. We present calibrated protocols for the robust folding of contiguous shapes from one or two rRNA subunits that are efficient to allow folding using crude extracts of total RNA. We also show that RNA maintains stability within the folded structure. Lastly, we present a novel and comprehensive analysis and insights into the stability of RNA:DNA origami nanostructures and demonstrate their enhanced stability when coated with polylysine-polyethylene glycol in different temperatures, low Mg^2+^ concentrations, human serum, and in the presence of nucleases (DNase I or RNase H). Thus, laying the foundation for their potential implementation in emerging biomedical applications, where folding rRNA into stable structures outside and inside cells would be desired.

**Graphical Abstract:**

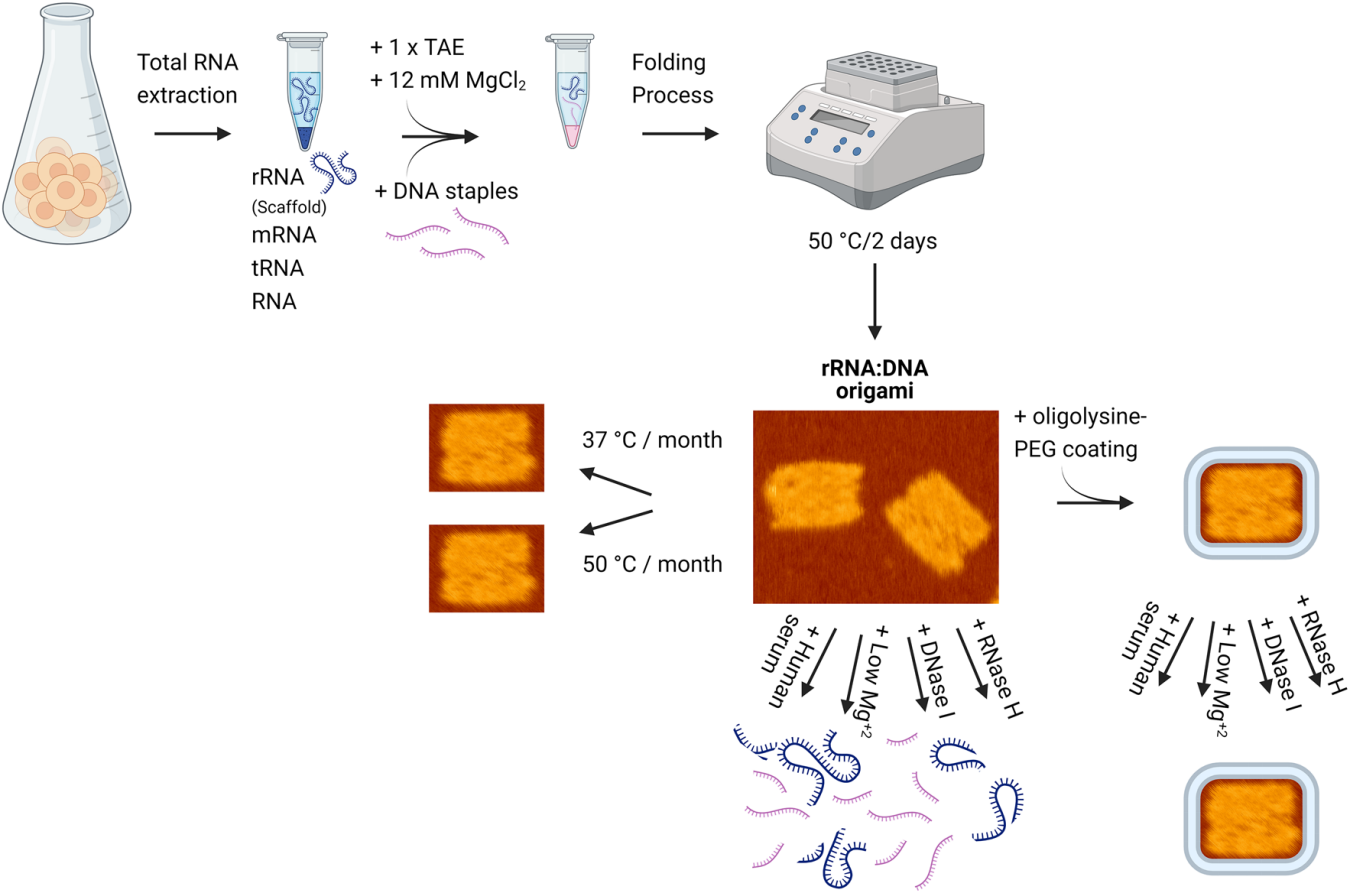

**Supplementary Information:**

The online version contains supplementary material available at 10.1186/s12951-024-02489-2.

## Introduction

The immobile DNA junction designed and synthesized first by Seeman nearly 4 decades ago [1], became the foundation for the entire field of DNA nanotechnology, which utilizes the physical and chemical properties of DNA for use as construction material rather than a carrier of genetic information. This uniquely-controllable nanostructure [2] and its derivatives have been investigated since, leading to a diverse range of DNA nanostructures in various applications [3–18].

DNA and RNA origami are promising technologies to fabricate artificial spatial nanostructures with programmable properties and functionalities with sub-nanometer precision, which have gained interest over the past two decades. In DNA origami, the folding of a single DNA scaffold is usually mediated by hybridization of many short DNA oligonucleotides termed staples [7], although unimolecular folding has also been demonstrated [6, 13, 19–21]. Efforts made in recent years have focused on techniques for manufacturing DNA origami in industrial scales [22, 23] and addressing pharmacological challenges [24, 25], as a prerequisite for its wider implementation [26–31].

The concept of “RNA origami” mainly refers to folding of shapes from single stranded RNA, while exploiting the assortment of RNA tertiary structural motifs in addition to canonical WC base pairing [19, 32]. RNA–DNA hybrid origami technique is conceptually similar to DNA origami [7] if considering the geometrical duplex differences (Additional file 1: Note S1), but has been much less explored. Ko et al. [33] were first to demonstrate nanofibers, 2D arrays, and 3D dodecahedra self-assembled from simple RNA–DNA branched nanomotifs assembled from 3 different strands. Wang et al. [34, 35] and Endo et al. [34, 35] describe preparation of hybrid origami using relatively short strands of artificially transcribed RNA with limited size, complexity and yield. Zheng et al. [36] reported hybrid nanowires ranging in size from 10 to 500 nm assembled from repetitive RNA sequences synthesized by rolling circle transcription and DNA staples. Recent works, demonstrated tube (~ 350 nt scaffold) [37], nanobrick (~ 401 nt scaffold) [38] and 3D wireframe origami hybrid nanostructures, such as tetrahedra, octahedron and bipyramids [39]. These reported structures were constructed from segments of mRNA (< 700 nt), genome RNA transcripts (< ~ 1000 nt) or a fragment of *E.Coli* 23S rRNA (1980 nt), which were all obtained by in-vitro transcription.

Synthetic RNA empirically differs from native RNA extracted from cells of tissue. Native cellular RNA undergoes a diverse variety of chemical modifications (~ 100 types of modification) [40] that are found in eukaryotes and prokaryotes and can significantly affect origami folding and the resulting structures. In particular, rRNA comprises a noticeable number of modifications, such as ribose sugar methylation, pseudouridylation and three additional base modifications: methylation, acetylation and aminocarboxypropylation [41].

Despite recent progress, the field of RNA:DNA origami remains in its nascent stages, with size, shape and complexity of nanostructures limited largely by scaffold constraints. Therefore, there is a pressing demand for low-cost and readily available sources of RNA, along with universal and scalable approaches for the fabrication and assembly of RNA–DNA nanostructures. Additionally, a comprehensive stability and integrity characterization of these nanostructures is critical to enable their use in emerging biomedical applications, where the effects of biological environments must be fully understood and considered.

This study aimed to highlight ribosomal RNA (rRNA) as a suitable and biologically-abundant precursor for building 2D and 3D origami nanostructures. rRNA is an essential component of the ribosomes of prokaryotes and eukaryotes. It comprises at least 80% of the total RNA across all cell types [42, 43], existing at tens of thousands to millions of copies per cell [44, 45], and representing approximately 1% of the total cell mass [46]. An individual prokaryotic cell has ~ 0.8 pg rRNA, whereas an eukaryotic cell contains 100-times more, ~ 8–20 pg rRNA [43, 47]. This makes the amount of rRNA per cell potentially higher by 3 to 4 orders of magnitude compared with a good yield of phage or plasmid DNA produced by a bacterial cell [47]. Furthermore, extracting total RNA from cells and tissues is relatively straightforward in bulk quantities [48].

Here we demonstrate the design and folding of hybrid rRNA-DNA molecular origami, showing that it could be utilized as an inexpensive and robust raw material. We present calibrated protocols for the robust folding of 2D and 3D contiguous shapes from one or two rRNA subunits from various organisms, and optimized designs that consider DNA-RNA geometry. We found that these protocols are sufficiently robust to allow efficient folding even using crude rRNA extracts, containing all the cellular RNA content, which can interfere with the assembly process. Remarkably, the long RNA scaffold strands are stable at room or higher temperatures when folded. Lastly, we introduce a full stability profile of rRNA:DNA structure in different biological environments (temperature, nucleases, human serum and low MgCl_2_ concentrations), and imply oligolysine-polyethylene glycol coating to improve stability and overcome nuclease degradation and low salt denaturation challenges as a prerequisite toward therapeutic application. Altogether, this work highlights rRNA as a suitable and abundant raw material for molecular origami, further expanding the methodological versatility of nucleic acid nanotechnology.

## Materials and methods

### rRNA scaffold

16S rRNA of *Escherichia coli (DH5α)* was purchased from Invitrogen™ (AM7940), while 18S and 26S rRNA from *Saccharomyces cerevisiae* were extracted from growing cells. The sequences of the scaffolds are listed in Additional file 1: Note S2.

### Staple oligonucleotides

DNA and RNA oligonucleotides staples were ordered from Integrated DNA Technologies (IDT) and reconstituted to 100 μM with ultrapure, DNase/RNase free water (Biological Industries, 01–869-1A). DNA oligonucleotides were stored at − 20 °C and RNA at − 80 ° C. The sequences of the oligonucleotides are listed in Additional file 1: Note S3.

### Total RNA extraction

Yeast cells (*S. cerevisiae*) were grown in 1500 mL of the appropriate medium to a density of approximately 1–2 ×${10}^{7}$ cells/mL (log phase) and pelleted by centrifugation. The pellet was resuspended in 30 mL of sterile saline (0.9% W/V, NaCl) and centrifuged at high speed. The pellet was resuspended in 3 mL of STE (0.32 M Sucrose, 20 mM Tris.Cl-pH 7.5, 10 mM EDTA-pH 8.0) and briefly vortexed in the presence of acid washed beads (Sigma #G8772). 18 mL of NTES (100 mM NaCl, 5 mM EDTA, 50 mM Tris.Cl-pH 7.5, 1% SDS) was added and the mixture briefly vortexed again. 15 mL of hot acidic phenol (Sigma #P4682, 65 °C) was added and the mixture was immediately vortexed and further incubated at 65 °C for 5 min with frequent vortexing. The aqueous phase of the mixture was removed and washed twice by centrifuging the mixture at high speed for 5 min, and the removing the aqueous phase and protein interface into about 15 mL aliquot of hot phenol and incubating at 65 °C for 2 min with frequent vortexing. The aqueous phase only was then removed into 12 mL of phenol/chloroform (1:1 phenol Sigma #P4682 and chloroform: isoamyl alcohol 24:1 Sigma #C0549) at room temperature, vortexed and spun down. The aqueous phase was re-extracted with 9 mL of chloroform (Sigma #C0549), vortexed and spun down. The aqueous phase was precipitated by the addition of 1/10 volume of 3 M sodium acetate (pH 5.2) (Ambion #AM9740), followed by 2.5 volumes of absolute ethanol, vortexed and incubated overnight at − 80 °C. Following precipitation, the mixture was centrifuged at high speed for 40 min. The pellet was washed in 30 mL 70% cold ethanol in DEPC, centrifuged at 4500 rpm for 10 min and briefly dried. RNA pellet was resuspended in 5 mL DEPC and left to be dissolved at 55 ºC for 10 min. RNA is then stored at − 20 to − 80 °C. Concentration and quality were estimated by spectrophotometry (O.D and 260/280 ratio).

### Ribosomal RNA purification

Total RNA was extracted from *Saccharomyces cerevisiae* as described above. 18S and 26S ribosomal RNA subunits were purified by running the total RNA in the AKTA explorer (Cytiva AKTA Start 29022094) or by gel extraction. Total RNA wes run on 1 × TAE, 1% agarose gel (Bio-Lab 000171235900) in 1 × TAE buffer (Invitrogen™ AM9869), following 18S extraction using Freeze 'N Squeeze™ DNA gel extraction spin columns (Bio-Rad 7326165) according to the manufacturer’s instructions.

### rRNA:DNA origami design

All the rectangular shapes were designed using caDNAno software [50] using the ribosomal RNA sequences as the scaffold strand. For combined rRNA:DNA rectangle design both 18S and 26S were used as one long scaffold strand. Adjustments of the crossover positions to fit the A-helix geometry found in RNA:DNA hybrids were done manually and described in detail in Additional file 1: Note S1. 2D and 3D cuboctahedrons were designed manually. See Additional file 1: Note S4 for full designs.

### rRNA:DNA origami folding

All shapes, besides the 2D and 3D cuboctahedrons, were folded at a 1:10 scaffold:staple ratio in 1 × TAE, 12 mM MgCl_2_ buffer. The samples were subjected to a thermal cycler (BioRad C1000 Touch Thermal Cycler) or heat-block (Major Science) and maintained at 50 °C over 2 days.

2D and 3D cuboctahedrons were folded differently. The scaffold and the staples strands were mixed at 1:10 ratio respectively, in 1 × TAE, 12.5 mM MgCl_2_ buffer. The samples were folded in a thermal cycler (BioRad C1000 Touch Thermal Cycler) according to adjusted protocol: 60 °C for 1 min, 55 °C for 5 min, followed by 10 min incubation at 50 °C, 37 °C and 25 °C. Folding reactions can be left at 37 °C over a few days to improve yields.

Additional tested folding protocols are described in Additional file 1: Note S5.

### Gel electrophoresis

Total RNA or rRNA were run on 1% agarose, 1 × TAE gel. The running buffer was 1 × TAE. rRNA:DNA origami samples were run on 1.5% agarose, 0.5 × TBE containing 10 mM MgCl_2_ gel in a cooled ice bath (80–100 V). The 0.5 × TBE running buffer also contained 10 mM MgCl_2_. Ethidium Bromide (Invitrogen 15585-011) was used to stain the RNA and origami structures. The Gels were imaged on a BioRad Gel Doc EZ™ Imager and analyzed on ImageLab v6.0.1 software.

### Atomic force microscopy

Folding quality and shape integrity were confirmed by atomic force microscopy (AFM), Brukers (JPK) NanoWizard ultra AFM III. 10–20 µl of 2–5 nM sample in folding buffer (1 × TAE, 12 mM MgCl_2_) was deposit on a freshly cleaved mica (TED PELLA, INC), and incubated at room temperature for 5–s10 min, following by gentle washing with 200 µl folding buffer (twice). Samples were scanned in 1 mL folding buffer in AC and HyperDrive mode using Ultra-Short cantilevers with force constant 0.3 N/m ordered from Nano World (USC-F0.3-k0.3).

2D and 3D cuboctahedrons were scanned slightly differently. Briefly, 3 µl of 25 mM Nickel (II) chloride hexahydrate (Hampton Research, HR2-687) were added to 20 µl of 10 nM sample. The solution was deposited on a mica for 5 min incubation, following one gentle wash with 50 µl folding buffer (1 × TAE, 12.5 mM MgCl_2_). Samples were scanned in 1 mL of folding buffer, with addition of 4 mM Nickel to keep the hybrid nanostructures attached to the mica. In addition to AC and HyperDrive modes, 3D samples were also scanned in QI mode using the same cantilevers. 16S rectangles were scanned similarly, however Nickel concentration was reduced to 1 mM. All obtained images were analyzed using JPK Data Processing v6.1.198.

### Folding efficiency and yields calculation

Folding efficiency was estimated by analyzing AFM images (200 nm scale). The numbers of well folded and misfolded shapes were calculated, and the yields were calculated as the fraction of the folded shapes out of the total.

### Folded shape purification

Upon folding, 18S rRNA:DNA rectangles were purified from staples’ excess and RNA leftovers using 100k ultra—0.5 mL centrifugal filter units with Ultracel-100 membrane (Merck Millipore, UFC510024, 100 kDa molecular weight cutoff). 3 washing steps were carried out using the folding buffer (1 TAE, 12 mM MgCl_2_) at 8×*g* over 5 min. Successful purification was verified by electrophoresis as described below.

Prior to K_10K_-PEG_5K_ coating, the 18S rRNA:DNA rectangles were purified as follows: separation of the high-molecular-weight DNA origami objects from the low-molecular-weight excess staple strands was performed using a 100k molecular weight cut-off membrane Amicon Ultra centrifugal filter device (Millipore). A total of three cycles of buffer exchange (1 × TAE and 10 mM MgCl_2_) and filtration were conducted using the following centrifugal parameters: 5000*g*, 5 min at room temperature. A detailed description of the calibration of the purification protocol is found in Additional file 1: Note S6.

### Thermal stability assay of *S.cerevisiae* rRNA scaffold

Total unpurified RNA extracted from *S.cerevisiae* diluted in 1 × TAE buffer supplemented with 12 mM MgCl_2_ to a final concentration of 20 nM. Next, the samples were maintained in temperature gradient (65 °C, 61 °C, 57.5 °C, 52.8 °C and 45 °C) for up to 1 h in a BioRad C1000 Touch Thermal Cycler. Samples were collected at the following time points: 0, 5, 10, 20, 30, 40 and 60 min, and analyzed on 1% agarose gel in a 1 × TAE buffer, at 80 V at room temperature. Scaffold stability and integrity was evaluated by migration and intensity of the bands relative to t = 0 time point, which served as 100%. Graphs were generated using GraphPad Prism (version 8.3.0).

All assays were performed in a thermal cycler (BioRad C1000 Touch Thermal Cycler). Shapes’ integrity was analyzed by gel electrophoresis and band intensity. The graphs were generated with GraphPad Prism (version 8.3.0).

### Loading Streptavidin on folded shapes

Purified 18S rRNA:RNA rectangles, having four staples comprising biotin on their 5′, were folded (using purified 18S *S. cerevisiae* rRNA) and purified as described above. Subsequently, shapes were incubated with Streptavidin at 1:10 ratio respectively over 2.5 h at room temperature. Samples were purified to discourage the free streptavidin prior scanning in atomic force microscopy.

### Coating of 18S rRNA:DNA with PEG Poly Lysine (K_10_–PEG_5K_)

K_10_-PEG_5K_ Coating was performed as reported previously by Ponnuswamy et al. [24]. Briefly, purified 18S rRNA:DNA rectangles were mixed with oligolysine-PEG (K_10_-PEG_5K_) at a final concentration of 1:360 with P:N ratio (phosphates in DNA:nitrogen in amines) of 1:1. Sample was then incubated at room temperature for 1 h, during which electrostatic interactions occurred between the negatively charged origami structure and positively charged lysine, resulting in coated nanostructures.

K_10_-PEG_5K_ was dissolved in 12 mM MgCl_2_, 40 mM Tris, 20 mM Acetate, 1 mM EDTA and purchased from tilibit nanosystems GmbH. Polydispersity index from gel permeation chromatography is between 1.00–1.20, and the average molecular weight as provided by the company is 6600 Da (Additional file 1: Note S7).

### Removal of the K_10_-PEG_5K_ coating shell from 18S rRNA:DNA rectangles

Removal was performed using Chondroitin sulfate sodium salt (Cat# C4384, Sigma), in an excess amount of 100 × the number of amines, while adjusting the final Mg^2+^ concentration to 12 mM as described by Ponnuswamy et al. [24]. The sample was incubated at 37 °C over 2 h. Removal of the K_10_-PEG_5K_ allows standard pattern migration of 18S rRNA:DNA in agarose gel for further analysis (Additional file 1: Note S7).

### 18S rRNA:DNA rectangles resistance against nucleases (DNase I and RNase H)

Bare and K_10_-PEG_5K_ coated 18S rRNA:DNA rectangles were diluted in a folding buffer (1 × TAE, 12 mM MgCl2) containing either 2 units of DNase I (DNase I, Cat# M0303S, NEB), or 1.25 units of RNase H (RNase H, Cat# M0297S, NEB),such that the final Mg^2+^ concentration was adjusted to 12 mM. Subsequently, the samples were incubated at 37 °C over 24 h in a thermal cycler. For DNase I experiments, samples were collected at the following time points: 0, 1 h, 2 h, 4 h, 8 h and 24 h, while for RNase H, samples were collected at time point of: 0, 0.5 h, 2 h and 4 h. All samples were analyzed using agarose gel as described above to assess the % of the stable structures based on band intensity. Total band intensity of each time point was normalized to time point 0 following subtraction of background signal. Quantification was performed using Image Lab gel analysis software (Bio-Rad, version 6.0.1).

### 18S rRNA:DNA rectangles stability in low Mg^2+^ concentration environments

Bare and K_10_–PEG_5K_ coated 18S rectangles were diluted in a folding buffer (1 × TAE, 12 mM MgCl_2_), such that the final Mg^2+^ concentrations were 12, 3, 1, 0.6 mM. The samples were incubated at 37 °C for 1 h and analyzed using gel electrophoresis as described above. Total band intensity of each magnesium concentration was normalized to 12 mM (which is the concentration used for folding these rectangles) following subtraction of background signal. Quantification was performed using Image Lab gel analysis software (Bio-Rad, version 6.0.1).

### 18S rRNA:DNA rectangles stability in human serum

Uncoated (bare) and K_10_-PEG_5K_ coated 18S rRNA:DNA rectangles were diluted in 10% human serum (Cat# H4522, Sigma) such that the final Mg^2+^ concentration was kept 12 mM. Next, the samples were incubated at 37 °C over 3 days in a thermal cycler. Samples were collected at the following time points: 0, 0.5, 1, 2, 4, 8, 24, 32, 48 and 72 h, and analyzed using agarose gel as described above to assess the % of the stable structures based on band intensity. Total band intensity of each time point was normalized to time point 0 following subtraction of background signal. Quantification was performed using Image Lab gel analysis software (Bio-Rad, version 6.0.1).

### Graphical abstract image

Graphical abstract image was created with biorender (BioRender.com).

## Results

As a proof of concept, we chose *Saccharomyces cerevisiae’s* 18S (1800 nt) and/or 26S (3396 nt) as scaffold strands for construction of our nanostructures. Following a standard RNA extraction protocol, we extracted 269 mg of total RNA from harvesting 21 L of yeast culture, equivalent to 35 gr of pellet. 58 mg of 18S rRNA and 72.5 mg of 26S rRNA were harvested **(**Additional file 1: Note S8).

**Fig. 1:**
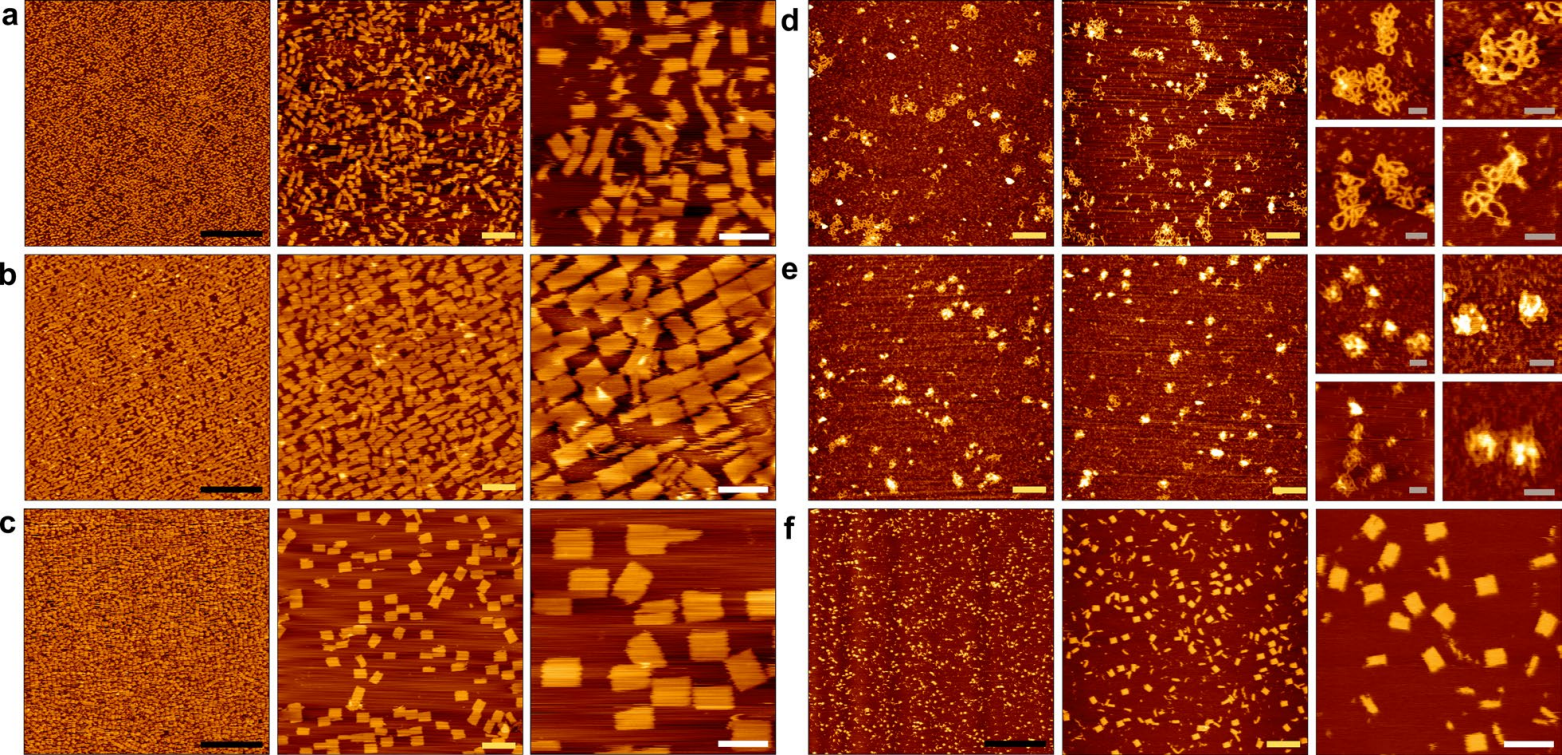
rRNA-DNAorigami nanostructure feasibility- AFM analysis. **a**, **b** rRNA-DNA origami rectangles folded using 18S or 26S rRNA (extracted from *S. cerevisiae*) as a scaffold respectively. **c** rRNA-DNA origami combined rectangle folded from both 18S and 26S subunits, which serve as one long scaffold strand and construct one shape. **d**, **e** 2D and 3D rRNA-DNA cuboctahedrons, open and close conformations respectively constructed from 26S rRNA subunit extracted from *S. cerevisiae*. **f** rRNA-DNA origami rectangles comprising 16S rRNA (extracted from *E. coli*) as a scaffold strand. Scale bar: black—1 µm, yellow—200 nm, white—100 nm and gray—50 nm

Unlike double-strand DNA, which adopts the canonical B-helix geometry, RNA–DNA hybrids form A-helix geometry with 11 bases/turn rather than 10.5 bases/turn [49]. To facilitate the shapes’ design we used caDNAno [11, 50], while adjusting staples’ crossover periodicity to 33-bases as previously described [34, 35] (Additional file 1: Note S1). Initially, we folded 18S and 26S into rRNA-DNA rectangles **(**Fig. 1a, b**)**. Next, by connecting the 18S and 26S rRNA subunits utilizing connector staples, we constructed “combined” rectangles with both rRNA units as a scaffold (5196 nt) (Fig. 1c). Drawing inspiration from the design of the DNA cuboctahedron [11], we fabricated more complex structures, 2D and 3D rRNA-DNA cuboctahedron, with open and closed conformations, respectively, using the 26S rRNA as scaffold (Fig. 1d, e). To showcase the simplicity and robustness of this method, we folded 16S rRNA (1542 nt) into rectangles using *E. coli* total RNA (Fig. 1f).

**Fig. 2:**
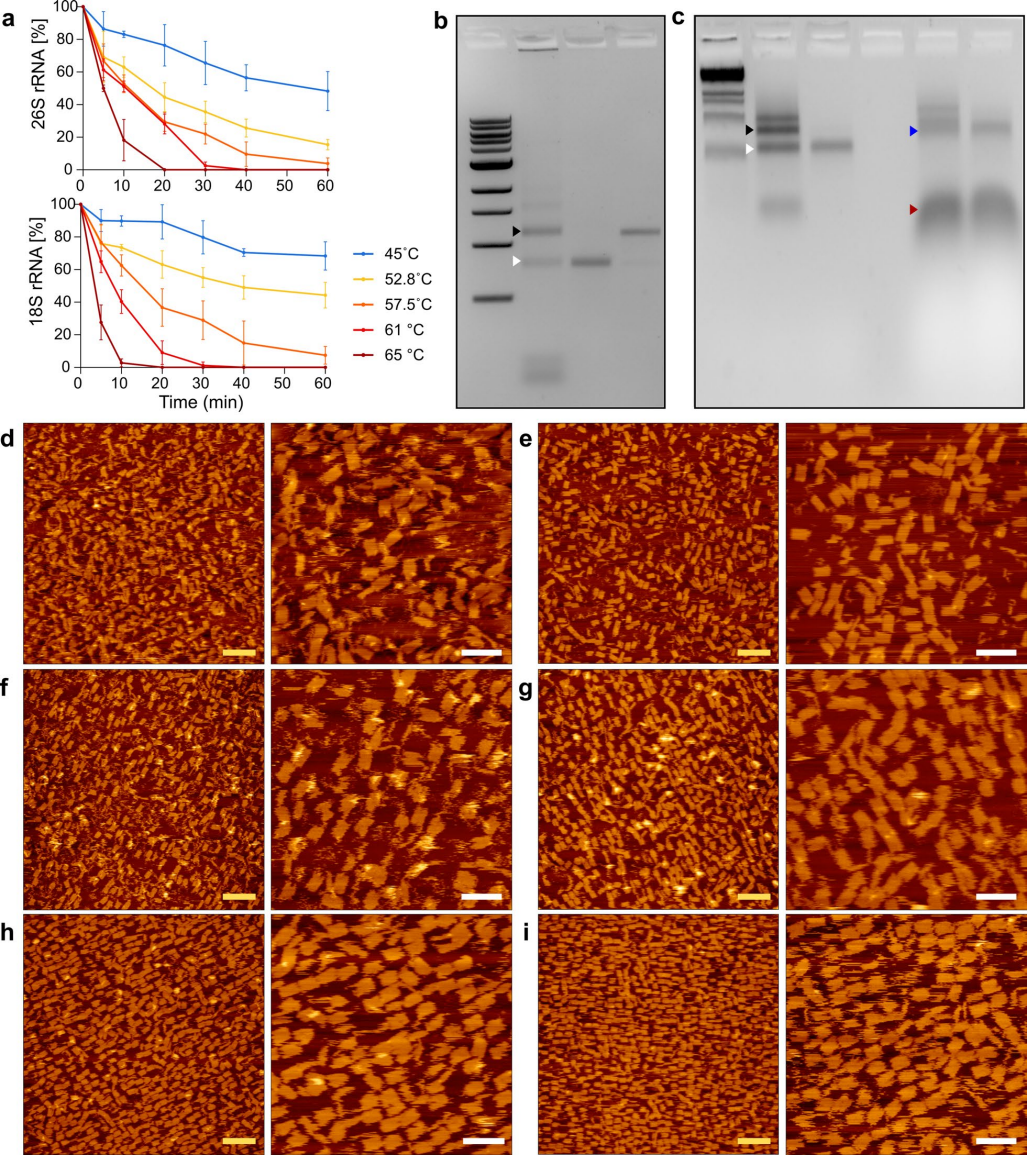
Protocol interrogation-AFM analysis. **a** Thermal stability of 18S and 26S rRNA subunits (*S. cerevisiae*) at a range of constant temperatures (65 °C–45 °C) over 1 h (top: 18S rRNA, bottom: 26S rRNA). **b** Gel electrophoresis of total RNA (lane 2), purified 18S subunit (lane 3) and 26S subunit (lane 4) rRNA extracted from *S. cerevisiae*. 4 main bands appear in total RNA sample run in lane 2, representing from top to bottom 26S precursor, 26S rRNA subunit (black arrow), 18S rRNA subunit (white arrow) and the rest extracted RNA molecules. Lane 1 contained a 1 kb ladder. **c,** Effect of scaffold purity on the assembly of 18S rRNA-DNA rectangles: lane 1 contained 1 kb ladder; lane 2 contained total RNA extracted from *S. cerevisiae*; lane 3 contained a purified 18S rRNA subunit. Lane 5 and 6 contain folding reactions of 18S rectangles from crude extracted total RNA or purified 18S scaffold respectively. The black arrow represents the bands of 26S subunits, the white arrow indicates the bands of 18S subunits, the blue arrow marks the bands of the folded 18S rectangles and the red arrow staples’ leftovers. The bottom smeared bands comprised “total” RNA molecules and DNA staple leftovers in lane 5 or DNA staple leftovers only in lane 6. **d**, **e**, 18S rectangles folded according to the following thermal annealing sequence: 60 °C/1 min, followed by 55 ºC/5 min, and 10 min at 50 °C, 37 °C and 25 °C. Sequentially, sample presented at **e** was held at 37 °C for additional 2.5 days. **f–i**, Kinetics of the folding of 18S rectangles at constant temperature (50 °C/2d) using crude extracted RNA from *S. cerevisiae* comprising 18S subunit (scaffold). The AFM scans were obtained at the following time points: **f–**6 h, **g–**1d, **h–**1.5d and **i–**2d. Scale bars: yellow—200 nm and white—100 nm

We next interrogated the key factors that affect the folding process. We started by assessing 18S and 26S rRNA thermal stability. RNA, known to be more susceptible to high temperatures compared to DNA, at time lags used in standard folding protocols. Our results revealed that rRNA subunits were near-complete degradation within 10 min at 65 °C, implying that this process happens faster at higher temperatures. Samples held at 61.6 °C degraded after 30 min, while those at 57.6 °C, 52.7 °C and 45 °C remained partially stable (20–80%). After an hour, samples at 57.5 °C exhibited extensive degradation (> 92% degradation), whereas partial stability was maintained at 52.7 °C and 45 °C. Notably, the 18S rRNA (52.7 °C-44%, 45 °C-68%) showed greater stability compared to 26S rRNA (52.7 °C-15%, 45 °C-48%) at these temperatures (Fig. 2a).

Relying on these results, we customized previously described folding protocol [35] to fold the 18S rectangle (thermal annealing sequence: 60 °C/1 min, following by 55 °C/5 min, and 10 min at 50 °C, 37 °C and 25 °C). Interestingly, we noticed that keeping the folded rectangles at 37 °C over a few days improved the shape integrity and folding efficiency tremendously from 28 to 63% after 2.5 days at 37 °C (Fig. 2d, e**,** Additional file 1: Note S9).

We next examined the effects of scaffold purity, MgCl_2_ concentration and removal of edge staples. No significant effect was apparent when folding 18S rRNA rectangles using total RNA as opposed to purified 18S rRNA subunit (Fig. 2b, c**)**. Moreover, increasing the concentration of MgCl_2_ and/or removing edge staples have not significantly improved the folding and shapes’ integrity (Additional file 1: Note S10).

To simplify and optimize the folding protocol we folded 18S and 26S rRNA rectangles at isothermal temperature of 50 °C over 2 days. Using our computational model [51] compatible with RNA–DNA melting table [52], we calculated the critical folding temperature as 53 °C for 18S rectangles and 56 °C for 26S rectangles. Considering the thermal stability of rRNA observed in our study, we decided to fold the shapes at a slightly lower temperature of 50 °C (Additional file 1: Note S11) [53–55]. Folding 18S rRNA:DNA rectangles at such conditions greatly improved the yields and shortened the protocol. After 6 h, the yield was 26.5% with partially folded shapes. After 1 day, the yield increased to 51%, and after an additional half day, it reached 79%. Worth mentioning that the yields are higher, as degraded 26S rRNA and other extracted RNA were counted as misfolded shapes (Fig. 2f–i).

**Fig. 3:**
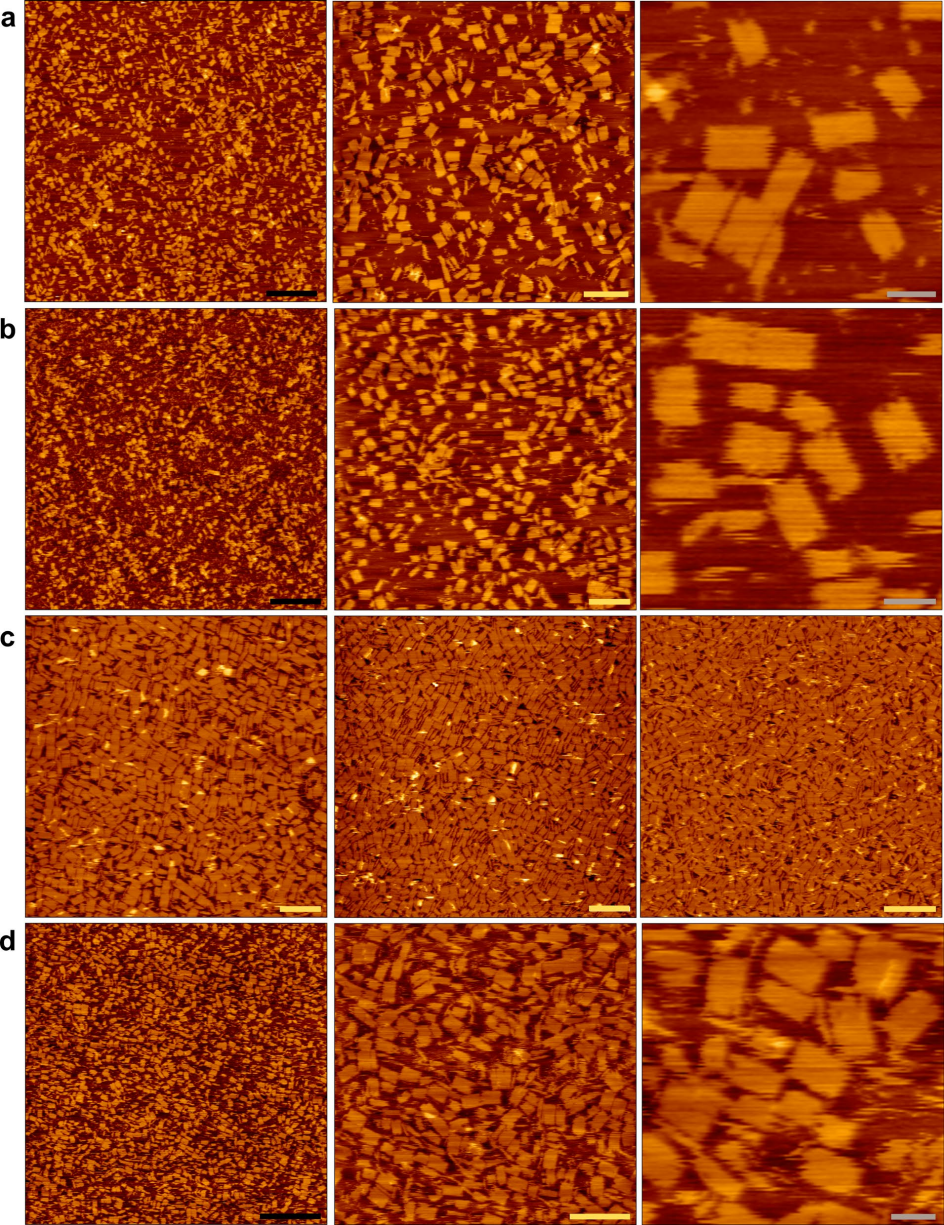
Simultaneous folding of both 18S and 26S rRNA into two discrete rectangles in one reaction as a feasibility for large scale folding. AFM analysis. **a**, **b** Decreasing scaffold:staple ratio—reducing scaffold to staple ratio from 1:10 (**a**) to 1:5 (**b**). The images presented (from left to right) at different scales: 500 nm, 200 nm and 50 nm. **c** Increasing scaffold concentration—increasing initial scaffold concentration in the folding reaction from 10 nM (left), to 50 nM (middle) and 80 nM (right). **d** Increasing volume reaction—18S and 26S rectangles folded simultaneously in 1.5 mL Eppendorf tubes in a heat block at 1:5 scaffold: staple ratio rather than using a thermal cycler. The images presented (from left to right) at different scales: Black—500 nm, yellow—200 nm and gray—50 nm

Lastly, we optimized the simultaneous bulk folding of both 18S and 26S rRNA into two discrete rectangles in a single reaction using crude extract of total RNA, serving as a testament to the feasibility and the robustness of our proposed method. First, we reduced the scaffold: staple ratio from 1:10 to 1:5 (Fig. 3a, b), and increased the initial concentration of the scaffold (18S concentration) from 10 to 50 nM and 80 nM (Fig. 3c). Respectively, the staples’ concentration also increased to maintain the 1:5 scaffold:staple ratio. Next, we increased the volume reaction, and carried the folding reaction in 1.5 mL (30 times larger than in a thermal cycler) folding buffer (1 × TAE buffer,12 mM MgCl_2_) over 2 days at constant temperature of 50 °C in a heat block, thus eliminating the need of a thermal cycler (Fig. 3d). AFM analysis confirmed the successful folding of both rectangles, 18S and 26S rectangles (Fig. 3). While, some misfolded shapes were observed, potentially attributable to the presence of cellular RNA molecules originating from the extraction process and the addition of two sets of staples. Nonetheless, when considering the overall amount of origami structures achievable rapidly and easily through this process, these misfolded structures are of negligible concern. The simplicity and robustness highlighted here suggest that this method could be easily implemented for folding origami at large scales.

Furthermore, we redesigned 4 core staples of the 18S rectangle and conjugated them with 5’-biotin. After assembly and purification, excess streptavidin was added. AFM analysis confirmed precise binding of Streptavidin at the intended target sites, emphasizing that rRNA:DNA nanostructures can be easily conjugated with various proteins or small molecules just as DNA origami [56] (Additional file 1: note S12).

Next, our objective was to thoroughly examine and obtain a comprehensive understanding of the stability profile of RNA:DNA hybrid nanostructures under conditions that are required for biomedical application. Stability of molecular origami is one of the main challenges holding nucleic acid nanotechnology back from wide implementation in various potential applications. While efforts have been made to overcome these challenges in the context of DNA origami, there has been no research conducted on the stability of RNA:DNA origami up to this point.

**Fig. 4.**
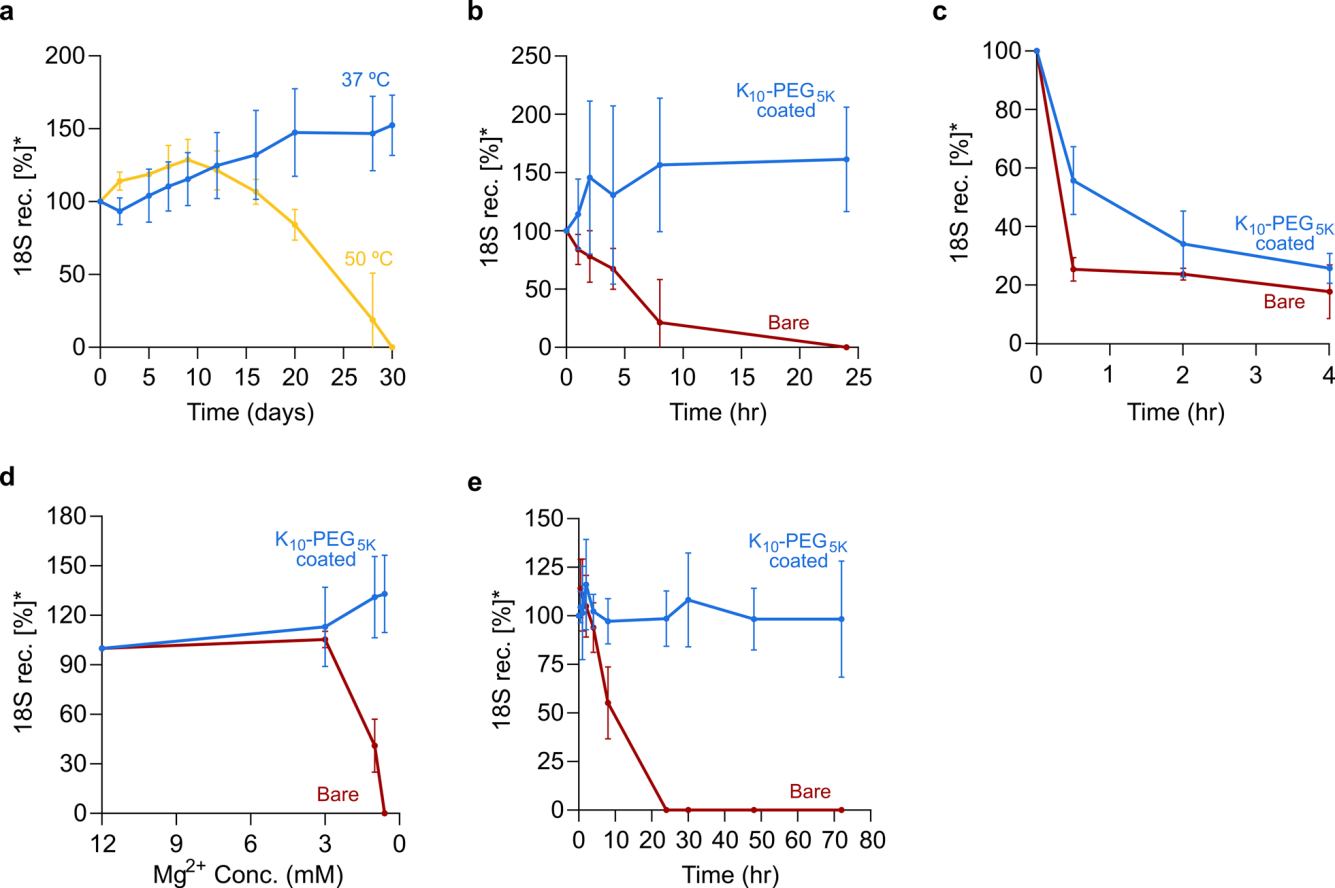
18S rRNA:DNA rectangles stability assays. **a** Shelf life, thermal stability of 18S rRNA-DNA rectangles at 37 °C (blue) and 50 °C (yellow) over a month. **b** DNase I assay. Stability profile of bare (red) and K_10_-PEG_5K_ coated (blue) 18S rRNA-DNA rectangles at 37 °C in the presence of DNase I over 24 h. **c** RNase H assay. Stability profile of bare (red) and K_10_-PEG_5K_ coated (blue) 18S rRNA-DNA rectangles at 37 °C in the presence of 1.25 units of RNase H over a period of 4 h. **d** Low Mg^+2^ concentration. Stability profile of bare (red) and K_10_-PEG_5K_ coated (blue) 18S rRNA-DNA rectangles at 37 °C in different Mg^+2^ concentrations over 1 h. **e** Human serum assay. Stability profile of bare (red) and K_10_-PEG_5K_ coated (blue) 18S rRNA-DNA rectangles at 37 °C in human serum over 72 h. (*) represents normalized amount of the folded 18S rectangles to time 0

We continued with 18S rRNA:DNA rectangles as a representative structure, and evaluated their stability in various biological conditions. First, we monitored these structures at 37 °C and 50 °C over a month. Our findings show that the structures remained stable at 37 °C after a month, while rectangles maintained at 50 °C started degrading after 15 days and reached 50% stability approximately at day 25. Interestingly, these results suggest that rRNA within the folded structure is more resistant to higher temperature than rRNA solely, as thermal stability profile of 18S rRNA scaffold showed 56% degradation at 52.7 °C or 32% degradation at 45 °C after 1 h (Figs. 2a, 4a, Additional file 1: Note S13). Moreover, both samples, rectangles held at 37 °C or at 50 °C, showed slight increases indicating that the assembly process of 18S rectangles was still occurring.

Subsequently, we challenged their stability in the presence of DNase I (2-units), known to be the most abundant nuclease in mammals [57]. We tested the structural stability of 18S rRNA:DNA rectangles and Rothmund’s DNA:DNA rectangles [7], which are ~ fourfold larger in size (1800 nt and 7249 nt respectively). DNA:DNA rectangles degraded entirely after 1 h, while rRNA:DNA rectangles showed prolonged resistance against nuclease digestion and ~ 85% of the structures remained stable over 1 h (Fig. 4b**,** Additional file 1: Note S14). Given these findings, we hypothesized that implementing polymer-based coating strategy, especially polyethylene glycol (PEG), can further increase the resistance of the rRNA:DNA structures to DNase I and other physiological conditions as has been previously reported in DNA:DNA origami nanostructures. PEGylation of DNA nanostructures address few of the challenges of structural DNA nanotechnology [25]. Ke et al. [58], showed a non-covalent uniform coating of DNA structures using intercalating PEG-tris-acridin, while Perrault and Shih increased in-vivo stability and circulation time of encapsulated DNA octahedrons using a PEGylated lipid bilayer [59]. Agarwal at el. electrostatically attached a cationic poly(ethylene glycol)-polylysine block copolymer to DNA nanostructures to improve stability [60]. Controllable PDMAEMA-PEG block copolymers origami coating was also shown to improve origamis’ biocompatibility [61]. Moreover, Ponnuswamet et al. [24], demonstrated increased stability at low salt conditions, fetal bovine serum and resistance to nuclease I, as well as improved cellular uptake and pharmacokinetics, by integrating oligolysine-PEG copolymer. Therefore, we decided to coat our 18S rRNA:DNA rectangles using oligolysine-PEG (K_10_-PEG_5K_) (Additional file 1: Note S7).

First we wanted to eliminate the potential effect of K_10_-PEG_5K_ on the folding process and shape of 18S rRNA:DNA rectangles, as polyamines can deform, condense and lead to aggregation. We validated successful folding and proper shapes using gel electrophoresis and AFM. The resulting images showed that neither the folding process nor the shape and yields of the 18S rectangles were affected by the K_10_-PEG_5K_ coating (Additional file 1: Note S15).

Following, we repeated the DNase I stability assay. This time, we compared bare and K_10_-PEG_5K_ coated 18S rectangles in a folding buffer in the presence of 2-units of DNase I at 37 °C over 24 h. Only ~ 20% of bare rectangles remained stable after 8 h, however coating of 18S rRNA:DNA rectangles using K_10_-PEG_5K_ greatly improved their resistance to DNase I, leading to full stability (Fig. 4b, Additional file 1: Note S16).

Subsequently, as our origami shapes represents hybrids of rRNA:DNA, we also conducted experiment to assess the resilience of 18S rectangles, both bare and K_10_-PEG_5K_, when exposed to RNase H. The 18S bare/coated rectangles were incubated in a folding buffer at 37 °C for 4 h in the presence of 1.25 units of RNase H. The differences between bare and coated rectangles were primarily notable during the first 1.5–2 h of incubation: K_10_-PEG_5K_ coated 18S rectangles exhibited augmented resistance to RNase H compared to bare rectangles. However, it is worth mentioning that the gap in the resistance between the coated and bare rectangle was smaller suggesting that the protective effect of the K_10_-PEG_5K_ layer was less effective against RNase H when compared to its effectiveness against other tested conditions **(**Fig. 4c, Additional file 1: Note S17 & 18). Encouraged by this result, we tackled the next obstacle that molecular origami is facing—low salt denaturation [25, 62]. Divalent cations play a crucial role in stabilizing DNA/RNA nanostructures [63]. Mg^2+^ minimizes the repulsive forces and provides electrostatic support that are essential for precise folding and maintenance of the intricate molecular nanostructures. In low Mg^2+^ concentration environments, molecular origami will ultimately undergo unraveling or disintegration. Therefore our subsequent purpose entailed examining the protective efficacy of shilling 18S rRNA:DNA rectangles using K_10_-PEG_5K_ against low salt denaturation, aiming to increase their suitability for a wide range of applications. While bare rectangles were almost 60% degraded at 1 mM Mg^+2^ and completely degraded at 0.6 mM Mg^2+^ after 1 h at 37 °C, the K_10_-PEG_5K_ coated rectangles remained completely stable under these Mg^2+^ concentrations (Fig. 4d).

Lastly, we investigated the stability of bare and K_10_-PEG_5K_ coated 18S rRNA:DNA rectangles in human serum. 10% of human serum was added to folded rectangles, and structure stability was measured at 37 °C over 3 days at different time points. Bare 18S rectangles reached ~ 55% degradation after 8 h and were completely degraded after 24 h, whereas the coated structures were well protected and exhibited 98% stability after 72 h (Fig. 4e, Additional file 1: Note S19).

## Discussion

In this work, we utilized rRNA from *S. cerevisiae* and *E. coli* as a robust and reliable construction material for arbitrary rRNA-DNA hybrid origami nanostructures. We demonstrated that rRNA is susceptible to higher temperatures, which led us to optimize and develop a scalable folding protocol at isothermal temperature in 1.5 mL in a heat block rather than thermal cycler, while achieving enhanced yields and integrity. Next, we loaded Streptavidin onto the 18S rectangles at specific positions using biotin-tagged staples, thus underscoring the potential of rRNA:DNA origami for conjugation with a wide range of molecules. Subsequently, we demonstrated a comprehensive stability profile of 18S rRNA:DNA rectangles in different conditions and showed their resilience at 37 °C and 50 °C for over a month. Lastly, we presented improved stability in low Mg^2+^ and human serum environments, and enhanced resistance against DNase I and RNase H, by employing K_10_-PEG_5K_ (oligolysine-PEG) coating, thus laying groundwork toward therapeutic and medical applications.

A point highlighted by our approach is the feasibility of industrial scale molecular origami manufacturing using rRNA. Although a DNA-based approach has been elegantly shown [22, 23], other approaches could expand our knowledge base and prove important in certain settings where crude chemical extraction is easier or cheaper to perform than biotechnological production. Total RNA can be extracted at low cost and at bulk quantities from abundant sources such as wasted food, particularly animal soft tissue meat, which is being wasted at an estimated rate of more than 70 million tons per year according to The Food and Agriculture Organization of the United Nations [64–67]. By scaling up the chemical extraction of total RNA and using it as-is the cost of this raw material could be reduced to 100$ per kilogram, underscoring the cost-effectiveness and robustness of this method for scaffold generation compared to existing techniques. In addition, this approach also embodies noteworthy principles of recycling and sustainability.

Regarding the necessity for short synthetic oligonucleotides, known as staples, in origami techniques, diverse strategies have already been proposed to minimize their costs [22, 68, 69]. Implementing these approaches with rRNA as scaffold can significantly reduce the production costs of molecular origamis even further. Moreover, recent techniques have presented unimolecular folding [19–21, 32], wherein long single stranded nucleic acid is designed to autonomously fold, thus eliminating the need for staples entirely. Therefore, the primary factor affecting the production costs is the selection of the scaffold.

Several additional points are highlighted by this approach. First, using rRNA, as any other cellular RNA, could dramatically expand the shape and function space of molecular origami. Second, integrating RNA into molecular origami could necessitate the refinement of current design tools such that they take into account the geometry of DNA-RNA double helices, which might not be optimally supported by DNA-DNA origami folding protocols.

A third interesting point is the possibility to direct folding *in-situ* in order to lock rRNA in a stable origami structure, effectively knocking out this RNA from functioning in the cell. Cellular RNA can be utilized for folding in-situ [70] under cellular conditions. This opens up a fascinating alternative route for RNA silencing that is categorically different from existing approaches such as antisense oligonucleotides and RNAi. Based on preliminary studies we conducted, the introduction and cellular uptake of RNA staples or transformation or of a plasmid that fold rRNA in-situ resulted in growth inhibition in certain bacterial strains **(**Additional file 1: Notes S20 & 21), as did the expression of genetically-encoded RNA staples locking rRNA subunits into an origami rectangle. A critical advantage of this method is that molecular origami exhibits resilience to faults, i.e., shapes still fold efficiently even when sequence mutations occur. This could render such an approach very challenging for microbial pathogens to counter by mutation, as antibiotics are routinely countered. Studies are currently underway in our lab to elucidate the potential and scope of this antimicrobial approach.

## Conclusions

In summary, this work highlights rRNA as an inexpensive and efficient precursor material for molecular origami, further expanding the methodological versatility of nucleic acid nanotechnology. Moreover, it opens potential new avenues for research into large-scale manufacturing of molecular origami and novel applications of this fascinating field.

### Supplementary Information

**Additional file 1.** Folding Molecular Origami from Ribosomal RNA.

## Data Availability

The raw data generated and/or analyzed during the current study are available from the corresponding author upon request.

## References

[1] Seeman NC (1982). Nucleic acid junctions and lattices. J Theor Biol.

[2] Seeman NC, Kallenbach NR (1983). Design of immobile nucleic acid junctions. Biophys J.

[3] Chen JH, Seeman NC (1991). Synthesis from DNA of a molecule with the connectivity of a cube. Nature.

[4] Fu TJ, Seeman NC (1993). DNA double-crossover molecules. Biochemistry.

[5] Winfree E, Liu F, Wenzler LA, Seeman NC (1998). Design and self-assembly of two-dimensional DNA crystals. Nature.

[6] Shih WM, Quispe JD, Joyce GF (2004). A 1.7-kilobase single-stranded DNA that folds into a nanoscale octahedron. Nature.

[7] Rothemund PWK (2006). Folding DNA to create nanoscale shapes and patterns. Nature.

[8] He Y, Ye T, Su M, Zhang C, Ribbe AE, Jiang W (2008). Hierarchical self-assembly of DNA into symmetric supramolecular polyhedra. Nature.

[9] Zheng J, Birktoft JJ, Chen Y, Wang T, Sha R, Constantinou PE (2009). From molecular to macroscopic via the rational design of a self-assembled 3D DNA crystal. Nature.

[10] Douglas SM, Dietz H, Liedl T, Högberg B, Graf F, Shih WM (2009). Self-assembly of DNA into nanoscale three-dimensional shapes. Nature.

[11] Zhang F, Jiang S, Wu S, Li Y, Mao C, Liu Y (2015). Complex wireframe DNA origami nanostructures with multi-arm junction vertices. Nat Nanotechnol.

[12] Douglas SM, Bachelet I, Church GM (2012). A logic-gated nanorobot for targeted transport of molecular payloads. Science.

[13] Wei B, Dai M, Yin P (2012). Complex shapes self-assembled from single-stranded DNA tiles. Nature.

[14] Ke Y, Ong LL, Shih WM, Yin P (2012). Three-dimensional structures self-assembled from DNA bricks. Science.

[15] Benson E, Mohammed A, Gardell J, Masich S, Czeizler E, Orponen P (2015). DNA rendering of polyhedral meshes at the nanoscale. Nature.

[16] Li Z, Liu L, Zheng M, Zhao J, Seeman NC, Mao C (2019). Making engineered 3D DNA crystals robust. J Am Chem Soc.

[17] Wang Y, Guo X, Kou B, Zhang L, Xiao S-J (2020). Small circular DNA molecules as triangular scaffolds for the growth of 3D single crystals. Biomolecules.

[18] Jun H, Zhang F, Shepherd T, Ratanalert S, Qi X, Yan H (2019). Autonomously designed free-form 2D DNA origami. Sci Adv.

[19] Han D, Qi X, Myhrvold C, Wang B, Dai M, Jiang S (2017). Single-stranded DNA and RNA origami. Science.

[20] Yang M, Bakker D, Raghu D, Li ITS (2023). A single strand: a simplified approach to DNA origami. Front Chem.

[21] Jia Y, Chen L, Liu J, Li W, Gu H (2021). DNA-catalyzed efficient production of single-stranded DNA nanostructures. Chem.

[22] Praetorius F, Kick B, Behler KL, Honemann MN, Weuster-Botz D, Dietz H (2017). Biotechnological mass production of DNA origami. Nature.

[23] Stahl E, Martin TG, Praetorius F, Dietz H (2014). Facile and scalable preparation of pure and dense DNA origami solutions. Angew Chem Int Ed Engl.

[24] Ponnuswamy N, Bastings MMC, Nathwani B, Ryu JH, Chou LYT, Vinther M (2017). Oligolysine-based coating protects DNA nanostructures from low-salt denaturation and nuclease degradation. Nat Commun.

[25] Ramakrishnan S, Ijäs H, Linko V, Keller A (2018). Structural stability of DNA origami nanostructures under application-specific conditions. Comput Struct Biotechnol J.

[26] Arora AA, de Silva C (2018). Beyond the smiley face: applications of structural DNA nanotechnology. Nano Rev Exp.

[27] Bila H, Kurisinkal EE, Bastings MMC (2019). Engineering a stable future for DNA-origami as a biomaterial. Biomater Sci.

[28] Kuzyk A, Jungmann R, Acuna GP, Liu N (2018). DNA origami route for nanophotonics. ACS Photonics.

[29] Zeng Y, Nixon RL, Liu W, Wang R (2021). The applications of functionalized DNA nanostructures in bioimaging and cancer therapy. Biomaterials.

[30] Dobrovolskaia MA, Bathe M (2021). Opportunities and challenges for the clinical translation of structured DNA assemblies as gene therapeutic delivery and vaccine vectors. Wiley Interdiscip Rev Nanomed Nanobiotechnol.

[31] Dey S, Fan C, Gothelf KV, Li J, Lin C, Liu L (2021). DNA origami. Nat Rev Methods Primers.

[32] Geary C, Rothemund PWK, Andersen ES (2014). A single-stranded architecture for cotranscriptional folding of RNA nanostructures. Science.

[33] Ko SH, Su M, Zhang C, Ribbe AE, Jiang W, Mao C (2010). Synergistic self-assembly of RNA and DNA molecules. Nat Chem.

[34] Endo M, Yamamoto S, Tatsumi K, Emura T, Hidaka K, Sugiyama H (2013). RNA-templated DNA origami structures. Chem Commun.

[35] Wang P, Ko SH, Tian C, Hao C, Mao C (2013). RNA-DNA hybrid origami: folding of a long RNA single strand into complex nanostructures using short DNA helper strands. Chem Commun.

[36] Zheng H-N, Ma Y-Z, Xiao S-J (2014). Periodical assembly of repetitive RNA sequences synthesized by rolling circle transcription with short DNA staple strands to RNA–DNA hybrid nanowires. Chem Commun.

[37] Wu X, Liu Q, Liu F, Wu T, Shang Y, Liu J (2021). An RNA/DNA hybrid origami-based nanoplatform for efficient gene therapy. Nanoscale.

[38] Zhou L, Chandrasekaran AR, Yan M, Valsangkar VA, Feldblyum JI, Sheng J (2021). A mini DNA-RNA hybrid origami nanobrick. Nanoscale Adv.

[39] Parsons MF, Allan MF, Li S, Shepherd TR, Ratanalert S, Zhang K (2023). 3D RNA-scaffolded wireframe origami. Nat Commun.

[40] Boccaletto P, Machnicka MA, Purta E, Piątkowski P, Bagiński B, Wirecki TK (2017). MODOMICS: a database of RNA modification pathways 2017 update. Nucleic Acids Res.

[41] Sharma S, Entian K-D. Chemical modifications of ribosomal RNA. Ribosome Biogenesis. 2022;149–66. 10.1007/978-1-0716-2501-9_9 PMC9761533 35796987

[42] Westermann AJ, Vogel J (2021). Cross-species RNA-seq for deciphering host-microbe interactions. Nat Rev Genet.

[43] von der Haar T (2008). A quantitative estimation of the global translational activity in logarithmically growing yeast cells. BMC Syst Biol.

[44] Warner JR (1999). The economics of ribosome biosynthesis in yeast. Trends Biochem Sci.

[45] Lodish. Ism T/A Molecular Cell Biology 4e. 2000.

[46] Marguerat S, Schmidt A, Codlin S, Chen W, Aebersold R, Bähler J (2012). Quantitative analysis of fission yeast transcriptomes and proteomes in proliferating and quiescent cells. Cell.

[47] Westermann AJ. Dual RNA-seq of Pathogen and Host: Duale RNA-Sequenzierung Eines Pathogens und Seines Wirts. 2014.

[48] Escherichia Coli and Salmonella: Cellular and Molecular Biology. 1996.

[49] Arnott S, Chandrasekaran R, Millane RP, Park HS (1986). RNA-RNA, DNA-DNA, and DNA-RNA polymorphism. Biophys J.

[50] Douglas SM, Marblestone AH, Teerapittayanon S, Vazquez A, Church GM, Shih WM (2009). Rapid prototyping of 3D DNA-origami shapes with caDNAno. Nucleic Acids Res.

[51] Shapiro A, Hozeh A, Girshevitz O, Abu-Horowitz A, Bachelet I (2015). Cooperativity-based modeling of heterotypic DNA nanostructure assembly. Nucleic Acids Res.

[52] Sugimoto N, Nakano S, Katoh M, Matsumura A, Nakamuta H, Ohmichi T (1995). Thermodynamic parameters to predict stability of RNA/DNA hybrid duplexes. Biochemistry.

[53] Sobczak J-PJ, Martin TG, Gerling T, Dietz H (2012). Rapid folding of DNA into nanoscale shapes at constant temperature. Science.

[54] Hartung J, McCann N, Doe E, Hayth H, Benkato K, Johnson MB (2023). Toehold-mediated shape transition of nucleic acid nanoparticles. ACS Appl Mater Interfaces.

[55] Halman JR, Satterwhite E, Roark B, Chandler M, Viard M, Ivanina A (2017). Functionally-interdependent shape-switching nanoparticles with controllable properties. Nucleic Acids Res.

[56] Voigt NV, Tørring T, Rotaru A, Jacobsen MF, Ravnsbaek JB, Subramani R (2010). Single-molecule chemical reactions on DNA origami. Nat Nanotechnol.

[57] Yang W (2011). Nucleases: diversity of structure, function and mechanism. Q Rev Biophys.

[58] Ke Y, Bellot G, Voigt NV, Fradkov E, Shih WM (2012). Two design strategies for enhancement of multilayer-DNA-origami folding: underwinding for specific intercalator rescue and staple-break positioning. Chem Sci.

[59] Perrault SD, Shih WM (2014). Virus-inspired membrane encapsulation of DNA nanostructures to achieve in vivo stability. ACS Nano.

[60] Agarwal NP, Matthies M, Gür FN, Osada K, Schmidt TL (2017). Block copolymer micellization as a protection strategy for DNA origami. Angew Chem Int Ed Engl.

[61] Kiviaho JK, Linko V, Ora A, Tiainen T, Järvihaavisto E, Mikkilä J (2016). Cationic polymers for DNA origami coating - examining their binding efficiency and tuning the enzymatic reaction rates. Nanoscale.

[62] Kielar C, Xin Y, Shen B, Kostiainen MA, Grundmeier G, Linko V (2018). On the stability of DNA origami nanostructures in low-magnesium buffers. Angew Chem Int Ed Engl.

[63] Chen Y, Wang P, Liu Y, Liu T, Xu Y, Zhu S (2018). Stability and recovery of DNA origami structure with cation concentration. Nanotechnology.

[64] What food is wasted? https://toogoodtogo.org/en/movement/knowledge/what-food-is-wasted. Accessed 5 Sep 2021

[65] Hegnsholt E, Unnikrishnan S, Pollmann-Larsen M, Askelsdottir B, Gerard M. Tackling the 1.6-Billion-Ton Food Loss and Waste Crisis. BCG Global; 2018. https://www.bcg.com/publications/2018/tackling-1.6-billion-ton-food-loss-and-waste-crisis. Accessed 5 Sep 2021

[66] Gustavsson J. Global Food Losses and Food Waste: Extent, Causes and Prevention : Study Conducted for the International Congress “Save Food!” at Interpack 2011 Düsseldorf, Germany. Food & Agriculture Organization of the UN (FAO); 2011.

[67] Swaminathan MS (2015). Food losses and food waste. Combat Hunger Achieve Food Security..

[68] Ducani C, Kaul C, Moche M, Shih WM, Högberg B (2013). Enzymatic production of “monoclonal stoichiometric” single-stranded DNA oligonucleotides. Nat Methods.

[69] Schmidt TL, Beliveau BJ, Uca YO, Theilmann M, Da Cruz F, Wu C-T (2015). Scalable amplification of strand subsets from chip-synthesized oligonucleotide libraries. Nat Commun.

[70] Delebecque CJ, Lindner AB, Silver PA, Aldaye FA (2011). Organization of intracellular reactions with rationally designed RNA assemblies. Science.

