# Supplementary material for: Folding molecular origami from ribosomal RNA
Source: J Nanobiotechnology. 2024 May 2;22:218. doi: 10.1186/s12951-024-02489-2 (PMC11067225; doi:10.1186/s12951-024-02489-2)
Supplement: Supplementary file 1 — Additional file 1. Folding Molecular Origami from Ribosomal RNA. [file 12951_2024_2489_MOESM1_ESM.docx]

**Supplementary Material**

1. Staple periodicity: A-Helix (RNA-DNA) vs. B-Helix (DNA-DNA) Page 1
2. List of RNA scaffolds Page 2
3. List of DNA/RNA staples Page 6
4. Design schemes of the rRNA:DNA origami structures Page 36
5. Additional tested folding protocols Page 42
6. Filtration and purification of folded 18S rRNA:DNA rectangles Page 47
7. K_10_-PEG_5K_ coating and uncoating using chondroitin sulfate of

18S rRNA:DNA rectangles Page 48

1. Total RNA extraction from  *S.cerevisiae*  Page 49
2. Improved folding yields and shaped integrity by maintaining the samples

at 37 ºC upon folding Page 50

1. The effect of MgCl_2_ concentration and edge staples on the folding of 18S rectangles Page 51
2. Critical folding temperature prediction of 18S and 26S rectangles assembly Page 52
3. Loading of 18S rRNA:DNA rectangles with Streptavidin Page 53
4. Shelf life of 18S rRNA:DNA rectangles at 37 ºC and 50 ºC Page 54
5. DNA-DNA rectangle resistance to DNase I Page 55
6. AFM images of K_10_-PEG_5K_ coated 18S rRNA-DNA rectangles Page 56
7. 18S rRNA:DNA rectangles tolerance to treatment with DNase I Page 57
8. 18S rRNA:DNA rectangles tolerance to treatment with different

amounts of RNase H Page 58

1. 18S rRNA:DNA rectangles tolerance to treatment with

1.25-units of RNase H Page 59

1. 18S rRNA:DNA rectangles stability in human serum Page 60
2. Growth inhibition of *Staphylococcus aureus* and *Escherichia coli* Page 61
3. Plasmid’s design and list of sequences for inhibiting *Escherichia coli* growth Page 68

**Supplementary note 1**

**Staple periodicity: A-Helix (RNA-DNA) vs. B-Helix (DNA-DNA)**

All described rRNA:DNA origami nanostructures, besides the 2D and 3D [cuboctahedron](https://www.google.com/search?rlz=1C1CHBD_enIL845IL845&sxsrf=ALeKk01xb7PYiS7YTd9DmdcF5PmuEl83WA:1606308171151&q=cuboctahedron&spell=1&sa=X&ved=2ahUKEwj6rMOf3J3tAhWR26QKHTWhCKcQkeECKAB6BAgPEDA), were designed using caDNAno software [^38^](https://paperpile.com/c/FGinqH/ijT7E). In order to adjust the software’s square lattice to fit the A-helix geometry (11 bp/turn) rather than the typical DNA double strand geometry (B-helix : 10.5 bp/turn) , we applied a frameshift of +1 bases to all the suggested staples’ crossover positions. This resulted in $\mathbf{c}+33\mathbf{n}$ crossover periodicity, instead of $\mathbf{c}+32\mathbf{n}$, where $\mathbf{c}$ represent the potential cross over positions and can be 0, 8, 16 or 24, while $\mathbf{n}$ represent the periodicity in a square lattice.


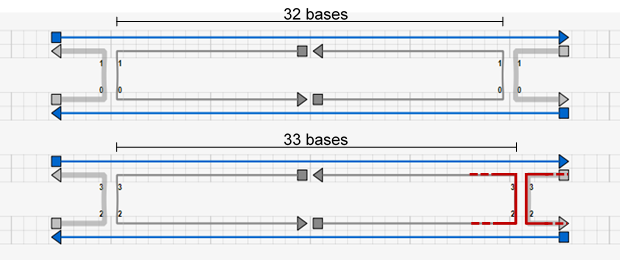


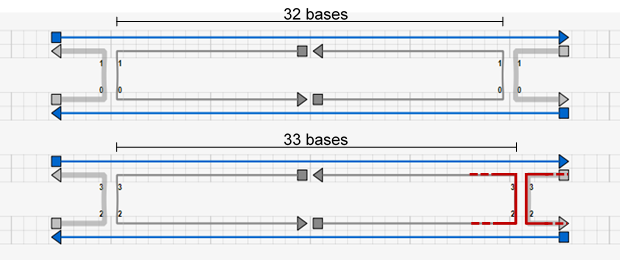


**Figure S1. Adjustment of crossover periodicity in caDNAno software.** **Top**, staples’ crossover periodicity suitable for B-helix geometry usually found in typical double stranded DNA. **Bottom**, staples’ crossover periodicity used in our designs and compatible with A-helix geometry found in RNA:DNA (as well as rRNA:DNA) hybrids and RNA:RNA.

**Supplementary note 2**

**List of RNA scaffolds**

18S *S. cerevisiae* rRNA (1800 bases):

>gi|2190509|emb|Z75578.1|:1-1800 S.cerevisiae 18S rRNA gene

UAUCUGGUUGAUCCUGCCAGUAGUCAUAUGCUUGUCUCAAAGAUUAAGCCAUGCAUGUCUAAGUAUAAGCAAUUUAUACAGUGAAACUGCGAAUGGCUCAUUAAAUCAGUUAUCGUUUAUUUGAUAGUUCCUUUACUACAUGGUAUAACUGUGGUAAUUCUAGAGCUAAUACAUGCUUAAAAUCUCGACCCUUUGGAAGAGAUGUAUUUAUUAGAUAAAAAAUCAAUGUCUUCGGACUCUUUGAUGAUUCAUAAUAACUUUUCGAAUCGCAUGGCCUUGUGCUGGCGAUGGUUCAUUCAAAUUUCUGCCCUAUCAACUUUCGAUGGUAGGAUAGUGGCCUACCAUGGUUUCAACGGGUAACGGGGAAUAAGGGUUCGAUUCCGGAGAGGGAGCCUGAGAAACGGCUACCACAUCCAAGGAAGGCAGCAGGCGCGCAAAUUACCCAAUCCUAAUUCAGGGAGGUAGUGACAAUAAAUAACGAUACAGGGCCCAUUCGGGUCUUGUAAUUGGAAUGAGUACAAUGUAAAUACCUUAACGAGGAACAAUUGGAGGGCAAGUCUGGUGCCAGCAGCCGCGGUAAUUCCAGCUCCAAUAGCGUAUAUUAAAGUUGUUGCAGUUAAAAAGCUCGUAGUUGAACUUUGGGCCCGGUUGGCCGGUCCGAUUUUUUCGUGUACUGGAUUUCCAACGGGGCCUUUCCUUCUGGCUAACCUUGAGUCCUUGUGGCUCUUGGCGAACCAGGACUUUUACUUUGAAAAAAUUAGAGUGUUCAAAGCAGGCGUAUUGCUCGAAUAUAUUAGCAUGGAAUAAUAGAAUAGGACGUUUGGUUCUAUUUUGUUGGUUUCUAGGACCAUCGUAAUGAUUAAUAGGGACGGUCGGGGGCAUCAGUAUUCAAUUGUCAGAGGUGAAAUUCUUGGAUUUAUUGAAGACUAACUACUGCGAAAGCAUUUGCCAAGGACGUUUUCAUUAAUCAAGAACGAAAGUUAGGGGAUCGAAGAUGAUCAGAUACCGUCGUAGUCUUAACCAUAAACUAUGCCGACUAGGGAUCGGGUGGUGUUUUUUUAAUGACCCACUCGGCACCUUACGAGAAAUCAAAGUCUUUGGGUUCUGGGGGGAGUAUGGUCGCAAGGCUGAAACUUAAAGGAAUUGACGGAAGGGCACCACCAGGAGUGGAGCCUGCGGCUUAAUUUGACUCAACACGGGGAAACUCACCAGGUCCAGACACAAUAAGGAUUGACAGAUUGAGAGCUCUUUCUUGAUUUUGUGGGUGGUGGUGCAUGGCCGUUCUUAGUUGGUGGAGUGAUUUGUCUGCUUAAUUGCGAUAACGAACGAGACCUUAACCUACUAAAUAGUGGUGCUAGCAUUUGCUGGUUAUCCACUUCUUAGAGGGACUAUCGGUUUCAAGCCGAUGGAAGUUUGAGGCAAUAACAGGUCUGUGAUGCCCUUAGACGUUCUGGGCCGCACGCGCGCUACACUGACGGAGCCAGCGAGUCUAACCUUGGCCGAGAGGUCUUGGUAAUCUUGUGAAACUCCGUCGUGCUGGGGAUAGAGCAUUGUAAUUAUUGCUCUUCAACGAGGAAUUCCUAGUAAGCGCAAGUCAUCAGCUUGCGUUGAUUACGUCCCUGCCCUUUGUACACACCGCCCGUCGCUAGUACCGAUUGAAUGGCUUAGUGAGGCCUCAGGAUCUGCUUAGAGAAGGGGGCAACUCCAUCUCAGAGCGGAGAAUUUGGACAAACUUGGUCAUUUAGAGGAACUAAAAGUCGUAACAAGGUUUCCGUAGGUGAACCUGCGGAAGGAUCAUUA

26S *S. cerevisiae* rRNA (3396 bases)

>NR_132209.1 Saccharomyces cerevisiae S288C 25S ribosomal RNA (RDN25-1), rRNA

GUUUGACCUCAAAUCAGGUAGGAGUACCCGCUGAACUUAAGCAUAUCAAUAAGCGGAGGAAAAGAAACCAACCGGGAUUGCCUUAGUAACGGCGAGUGAAGCGGCAAAAGCUCAAAUUUGAAAUCUGGUACCUUCGGUGCCCGAGUUGUAAUUUGGAGAGGGCAACUUUGGGGCCGUUCCUUGUCUAUGUUCCUUGGAACAGGACGUCAUAGAGGGUGAGAAUCCCGUGUGGCGAGGAGUGCGGUUCUUUGUAAAGUGCCUUCGAAGAGUCGAGUUGUUUGGGAAUGCAGCUCUAAGUGGGUGGUAAAUUCCAUCUAAAGCUAAAUAUUGGCGAGAGACCGAUAGCGAACAAGUACAGUGAUGGAAAGAUGAAAAGAACUUUGAAAAGAGAGUGAAAAAGUACGUGAAAUUGUUGAAAGGGAAGGGCAUUUGAUCAGACAUGGUGUUUUGUGCCCUCUGCUCCUUGUGGGUAGGGGAAUCUCGCAUUUCACUGGGCCAGCAUCAGUUUUGGUGGCAGGAUAAAUCCAUAGGAAUGUAGCUUGCCUCGGUAAGUAUUAUAGCCUGUGGGAAUACUGCCAGCUGGGACUGAGGACUGCGACGUAAGUCAAGGAUGCUGGCAUAAUGGUUAUAUGCCGCCCGUCUUGAAACACGGACCAAGGAGUCUAACGUCUAUGCGAGUGUUUGGGUGUAAAACCCAUACGCGUAAUGAAAGUGAACGUAGGUUGGGGCCUCGCAAGAGGUGCACAAUCGACCGAUCCUGAUGUCUUCGGAUGGAUUUGAGUAAGAGCAUAGCUGUUGGGACCCGAAAGAUGGUGAACUAUGCCUGAAUAGGGUGAAGCCAGAGGAAACUCUGGUGGAGGCUCGUAGCGGUUCUGACGUGCAAAUCGAUCGUCGAAUUUGGGUAUAGGGGCGAAAGACUAAUCGAACCAUCUAGUAGCUGGUUCCUGCCGAAGUUUCCCUCAGGAUAGCAGAAGCUCGUAUCAGUUUUAUGAGGUAAAGCGAAUGAUUAGAGGUUCCGGGGUCGAAAUGACCUUGACCUAUUCUCAAACUUUAAAUAUGUAAGAAGUCCUUGUUACUUAAUUGAACGUGGACAUUUGAAUGAAGAGCUUUUAGUGGGCCAUUUUUGGUAAGCAGAACUGGCGAUGCGGGAUGAACCGAACGUAGAGUUAAGGUGCCGGAAUACACGCUCAUCAGACACCACAAAAGGUGUUAGUUCAUCUAGACAGCCGGACGGUGGCCAUGGAAGUCGGAAUCCGCUAAGGAGUGUGUAACAACUCACCGGCCGAAUGAACUAGCCCUGAAAAUGGAUGGCGCUCAAGCGUGUUACCUAUACUCUACCGUCAGGGUUGAUAUGAUGCCCUGACGAGUAGGCAGGCGUGGAGGUCAGUGACGAAGCCUAGACCGUAAGGUCGGGUCGAACGGCCUCUAGUGCAGAUCUUGGUGGUAGUAGCAAAUAUUCAAAUGAGAACUUUGAAGACUGAAGUGGGGAAAGGUUCCACGUCAACAGCAGUUGGACGUGGGUUAGUCGAUCCUAAGAGAUGGGGAAGCUCCGUUUCAAAGGCCUGAUUUUAUGCAGGCCACCAUCGAAAGGGAAUCCGGUUAAGAUUCCGGAACCUGGAUAUGGAUUCUUCACGGUAACGUAACUGAAUGUGGAGACGUCGGCGCGAGCCCUGGGAGGAGUUAUCUUUUCUUCUUAACAGCUUAUCACCCCGGAAUUGGUUUAUCCGGAGAUGGGGUCUUAUGGCUGGAAGAGGCCAGCACCUUUGCUGGCUCCGGUGCGCUUGUGACGGCCCGUGAAAAUCCACAGGAAGGAAUAGUUUUCAUGCCAGGUCGUACUGAUAACCGCAGCAGGUCUCCAAGGUGAACAGCCUCUAGUUGAUAGAAUAAUGUAGAUAAGGGAAGUCGGCAAAAUAGAUCCGUAACUUCGGGAUAAGGAUUGGCUCUAAGGGUCGGGUAGUGAGGGCCUUGGUCAGACGCAGCGGGCGUGCUUGUGGACUGCUUGGUGGGGCUUGCUCUGCUAGGCGGACUACUUGCGUGCCUUGUUGUAGACGGCCUUGGUAGGUCUCUUGUAGACCGUCGCUUGCUACAAUUAACGAUCAACUUAGAACUGGUACGGACAAGGGGAAUCUGACUGUCUAAUUAAAACAUAGCAUUGCGAUGGUCAGAAAGUGAUGUUGACGCAAUGUGAUUUCUGCCCAGUGCUCUGAAUGUCAAAGUGAAGAAAUUCAACCAAGCGCGGGUAAACGGCGGGAGUAACUAUGACUCUCUUAAGGUAGCCAAAUGCCUCGUCAUCUAAUUAGUGACGCGCAUGAAUGGAUUAACGAGAUUCCCACUGUCCCUAUCUACUAUCUAGCGAAACCACAGCCAAGGGAACGGGCUUGGCAGAAUCAGCGGGGAAAGAAGACCCUGUUGAGCUUGACUCUAGUUUGACAUUGUGAAGAGACAUAGAGGGUGUAGAAUAAGUGGGAGCUUCGGCGCCAGUGAAAUACCACUACCUUUAUAGUUUCUUUACUUAUUCAAUGAAGCGGAGCUGGAAUUCAUUUUCCACGUUCUAGCAUUCAAGGUCCCAUUCGGGGCUGAUCCGGGUUGAAGACAUUGUCAGGUGGGGAGUUUGGCUGGGGCGGCACAUCUGUUAAACGAUAACGCAGAUGUCCUAAGGGGGGCUCAUGGAGAACAGAAAUCUCCAGUAGAACAAAAGGGUAAAAGCCCCCUUGAUUUUGAUUUUCAGUGUGAAUACAAACCAUGAAAGUGUGGCCUAUCGAUCCUUUAGUCCCUCGGAAUUUGAGGCUAGAGGUGCCAGAAAAGUUACCACAGGGAUAACUGGCUUGUGGCAGUCAAGCGUUCAUAGCGACAUUGCUUUUUGAUUCUUCGAUGUCGGCUCUUCCUAUCAUACCGAAGCAGAAUUCGGUAAGCGUUGGAUUGUUCACCCACUAAUAGGGAACGUGAGCUGGGUUUAGACCGUCGUGAGACAGGUUAGUUUUACCCUACUGAUGAAUGUUACCGCAAUAGUAAUUGAACUUAGUACGAGAGGAACAGUUCAUUCGGAUAAUUGGUUUUUGCGGCUGUCUGAUCAGGCAUUGCCGCGAAGCUACCAUCCGCUGGAUUAUGGCUGAACGCCUCUAAGUCAGAAUCCAUGCUAGAACGCGGUGAUUUCUUUGCUCCACACAAUAUAGAUGGAUACGAAUAAGGCGUCCUUGUGGCGUCGCUGAACCAUAGCAGGCUAGCAACGGUGCACUUGGCGGAAAGGCCUUGGGUGCUUGCUGGCGAAUUGCAAUGUCAUUUUGCGUGGGGAUAAAUCAUUUGUAUACGACUUAGAUGUACAACGGGGUAUUGUAAGCAGUAGAGUAGCCUUGUUGUUACGAUCUGCUGAGAUUAAGCCUUUGUUGUCUGAUUUGU

16S E. coli BL21 DE3 rRNA (1542 bases)

>CP001509.3:3832055-3833596 Escherichia coli BL21(DE3), complete genome

AAAUUGAAGAGUUUGAUCAUGGCUCAGAUUGAACGCUGGCGGCAGGCCUAACACAUGCAAGUCGAACGGUAACAGGAAACAGCUUGCUGcUUCGCUGACGAGUGGCGGACGGGUGAGUAAUGUCUGGGAAACUGCCUGAUGGAGGGGGAUAACUACUGGAAACGGUAGCUAAUACCGCAUAACGUCGCAAGACCAAAGAGGGGGACCUUaGGGCCUCUUGCCAUCGGAUGUGCCCAGAUGGGAUUAGCUAGUAGGUGGGGUAACGGCUCACCUAGGCGACGAUCCCUAGCUGGUCUGAGAGGAUGACCAGCCACACUGGAACUGAGACACGGUCCAGACUCCUACGGGAGGCAGCAGUGGGGAAUAUUGCACAAUGGGCGCAAGCCUGAUGCAGCCAUGCCGCGUGUAUGAAGAAGGCCUUCGGGUUGUAAAGUACUUUCAGCGGGGAGGAAGGGAGUAAAGUUAAUACCUUUGCUCAUUGACGUUACCCGCAGAAGAAGCACCGGCUAACUCCGUGCCAGCAGCCGCGGUAAUACGGAGGGUGCAAGCGUUAAUCGGAAUUACUGGGCGUAAAGCGCACGCAGGCGGUUUGUUAAGUCAGAUGUGAAAUCCCCGGGCUCAACCUGGGAACUGCAUCUGAUACUGGCAAGCUUGAGUCUCGUAGAGGGGGGUAGAAUUCCAGGUGUAGCGGUGAAAUGCGUAGAGAUCUGGAGGAAUACCGGUGGCGAAGGCGGCCCCCUGGACGAAGACUGACGCUCAGGUGCGAAAGCGUGGGGAGCAAACAGGAUUAGAUACCCUGGUAGUCCACGCCGUAAACGAUGUCGACUUGGAGGUUGUGCCCUUGAGGCGUGGCUUCCGGAGCUAACGCGUUAAGUCGACCGCCUGGGGAGUACGGCCGCAAGGUUAAAACUCAAAUGAAUUGACGGGGGCCCGCACAAGCGGUGGAGCAUGUGGUUUAAUUCGAUGCAACGCGAAGAACCUUACCUGGUCUUGACAUCCACGGAAGUUUUCAGAGAUGAGAAUGUGCCUUCGGGAACCGUGAGACAGGUGCUGCAUGGCUGUCGUCAGCUCGUGUUGUGAAAUGUUGGGUUAAGUCCCGCAACGAGCGCAACCCUUAUCCUUUGUUGCCAGCGGUCCGGCCGGGAACUCAAAGGAGACUGCCAGUGAUAAACUGGAGGAAGGUGGGGAUGACGUCAAGUCAUCAUGGCCCUUACGACCAGGGCUACACACGUGCUACAAUGGCGCAUACAAAGAGAAGCGACCUCGCGAGAGCAAGCGGACCUCAUAAAGUGCGUCGUAGUCCGGAUUGGAGUCUGCAACUCGACUCCAUGAAGUCGGAAUCGCUAGUAAUCGUGGAUCAGAAUGCCACGGUGAAUACGUUCCCGGGCCUUGUACACACCGCCCGUCACACCAUGGGAGUGGGUUGCAAAAGAAGUAGGUAGCUUAACCUUCGGGAGGGCGCUUACCACUUUGUGAUUCAUGACUGGGGUGAAGUCGUAACAAGGUAACCGUAGGGGAACCUGCGGUUGGAUCACCUCCUUA**Supplementary note 3**

**List of DNA/RNA staples**

DNA staples of 18S (*S. cerevisiae*) rRNA:DNA rectangles (11-fold geometry)

| Name | Sequence |
| --- | --- |
| core_01 | GTTTGTCCAAATTCTCC |
| core_02 | GCTTGAAACCGATAGTCGAAGAGCAATAATTACA |
| core_03 | CCGTTACCCGTTGAAAAATGAACCATCGCCAG |
| core_04 | GAGCCACAAGGACTCATATTGGAGCTGGAATT |
| core_05 | ATACTTAGACATGCAT |
| core_06 | TAAGCCATTCAATCGGT |
| core_07 | GGTATCTGATCATCTTCCAAATCACTCCACCAAC |
| core_08 | ACCACTATTTAGTAGGTAAGAACGGCCATGCA |
| core_09 | ACGATAACTGATTTAATTCTAATAAATACATCTC |
| core_10 | CTGGTGGTGTAGCGCGCGTGCGGCCCCGGCCAAG |
| core_11 | GGCTTAATCTTTGAGACCACAAGGCCATGCGATT |
| core_12 | CCAGTACACGAAAAAATTTCTTGATTAATGAAAA |
| core_13 | GAGCCATTCGCAGTTT |
| core_14 | TCACAGACCTGTTATTGACGACGGAGTTTCACAA |
| core_15 | CGACCATACTCCCCCCCATTACGATGGTCCTA |
| core_16 | ATGTGGTAGCCGTTTCTACCGCGGCTGCTGGCAC |
| core_17 | TTGGGTAATTTGCGCGCTTGTTCCTCGTTAAGGT |
| core_18 | ATATTCGAGCAATACGATTTACATTGTACTCA |
| core_19 | AATCGAACCCTTATTCCACAACTTTAATATACGC |
| core_20 | GCCCCCTTCTCTAAGCA |
| core_21 | TTCACCTACGGAAACCGATTACCAAGACCTCT |
| core_22 | AGGAACTATCAAATAA |
| core_23 | CCATGGTAGGCCACTATGCCCAAAGTTCAACTAC |
| core_24 | AGGTTAGCCAGAAGGAATTTCGCAGTAGTTAGTC |
| core_25 | GCAGGATCAACCAGATAGATAGGGCAGAAATTTG |
| core_26 | AATTTTTTCAAAGTAACAGACTTGCCCTCCAA |
| core_27 | CTGCTGCCTTCCTTGGAGACATTGATTTTTTA |
| core_28 | TTGTTACGACTTTTAGT |
| core_29 | AAGTCCTGGTTCGCCAAATTTCACCTCTGACAAT |
| core_30 | CTAGTCGGCATAGTTTACCACCACCCACAAAATC |
| core_31 | AAGCATATGACTACTG |
| core_32 | ATGTATTAGGTTATTTATTGTCACTATTCCAATT |
| core_33 | CCTCCCTGAATTAGGATTCCAAAGGGTCGAGA |
| core_34 | CCTCAAACTTCCATCGTGTCTGGACCTGGTGA |
| core_35 | ATTTCTCGTAAGGTGCTGAATACTGATGCCCC |
| core_36 | CGAGTGGGTCATTAAAATCTGTCAATCCTTATTG |
| core_37 | TCCTCTAAATGACCAAATGCTCTATCCCCAGC |
| core_38 | TGGTTAAGACTACGACCGTCCTTGGCAAATGC |
| core_39 | GATCCTGAGGCCTCACACGTAATCAACGCAAG |
| core_40 | TTAAGGTCTCGTTCGTTGTGTACAAAGGGCAGGG |
| core_41 | CAGGCTCCCTCTCCGGCGAAAAGTTATTATGA |
| core_42 | GTTATACCATGTAGTAATTTTAAGC |
| core_43 | GCTCTGAGATGGAGTTCTAGGAATTCCTCGTT |
| core_44 | CCTGCTTTGAACACTCTCGACCGTCCCTATTAAT |
| core_45 | AACCAGCAAATGCTAGCCTGATGACTTGCGCTTA |
| core_46 | CAAAATAGATTTAAGTTTCAGCCTTGCTCCACTC |
| core_47 | CCTCTAAGAAGTGGATAAGAAAGAGCTCTCAA |
| core_48 | AGAACGTCTAAGGGCACAAATTAAGCCGCAGG |
| core_49 | AGGCCCCGTTGGAAATGAGCTTTTTAACTGCA |
| core_50 | AGAACCCAAAGACTTTGGTTTCCCCGTGTTGAGT |
| core_51 | AAACACCACCCGATCCTTCAATAAATCCAAGA |
| core_52 | CACTGTATAAATTGCTTATCATCAAAGAGTCCGA |
| core_53 | ACAAGACCCTATTATTCCATGCTAATGAAACCAA |
| core_54 | GTTAGACTCAATGATCCTTCCGCAGG |
| egde_01 | CGAAAGTT |
| egde_02 | TTTTTTTTAACTTTCGCGGACCGGTTTTTTTT |
| egde_05 | ttttttttCGGGCGGTATCGCAATtttttttt |
| egde_06 | ttttttttCCAACCGGCCTACCATtttttttt |
| egde_09 | ttttttttTAAGCAGAGATCCCCTtttttttt |
| egde_12 | TAAGCCATTCAATCGGTACTAGCGAtttttttt |
| egde_03 | ttttttttTCAATTCCACCAAACGtttttttt |
| egde_04 | ttttttttCCTGTATCCTCTAGAAtttttttt |
| egde_07 | GCTGGCTCtttttttt |
| egde_08 | ttttttttCGTCAGTGCCCTTCCGtttttttt |
| egde_10 | ttttttttTTACCACA |
| egde_11 | ttttttttTCCTATTCGAATGGGCtttttttt |

Biotinylated DNA staples used for loading of 18S (*S. cerevisiae*) rRNA:DNA rectangles (11-fold geometry)

| Name | Sequence |
| --- | --- |
| core_4_5'Bio | /5BiotinTEG/CCTCTAAGAAGTGGATAAGAAAGAGCTCTCAA |
| core_48_5'Bio | /5BiotinTEG/AGAACGTCTAAGGGCACAAATTAAGCCGCAGG |
| core_03_5'Bio | /5BiotinTEG/CCGTTACCCGTTGAAAAATGAACCATCGCCAG |
| core_27_5'Bio | /5BiotinTEG/CTGCTGCCTTCCTTGGAGACATTGATTTTTTA |

DNA staples of 26S (*S. cerevisiae*) rRNA:DNA rectangles (11-fold geometry)

| Name | Sequence |
| --- | --- |
| core_01 | GACGGTCTAACAAATCAGACAACAAA |
| core_02 | CGAGGGACTAAAGGATCCATGGATTCTGACTTAG |
| core_03 | AAATTTGAGCTTTTGCCGCTATCGGTCTCTCGCC |
| core_04 | GAGTCAAGCTCAACAGGGGATCAGCCCCGAATGG |
| core_05 | GCTTCACTCGCCGTTA |
| core_06 | GATTCTGCCAAGCCCGTGCCAAACTCCCCACCTG |
| core_07 | GACGAGGCATTTGGCTATCAAGGGGGCTTTTACC |
| core_08 | AAGCTCTTCATTCAAAGTTCACTTTCATTACG |
| core_09 | GTATTCCCACAGGCTATCCCAACAGCTATGCTCT |
| core_10 | TGTCCACGTTCAATTAAAGCGTGTATTCCGGCAC |
| core_11 | AATACTTACCGAGGCAATCACTGTACTTGTTC |
| core_12 | GGGACAGTGGGAATCTACATTCAGAGCACTGG |
| core_13 | GAGCTTCTGCTATCCTGTTTCCTCTGGCTTCA |
| core_14 | TGTTCCAAGGAACATA |
| core_15 | CATTTGAATATTTGCTTACACACTCCTTAGCG |
| core_16 | AACGCTTGACTGCCACATCAGACAGCCGCAAAAA |
| core_17 | AAGTTGCCCTCTCCAA |
| core_18 | ACTACCACCAAGATCTGTAAGACCCCATCTCCGG |
| core_19 | GACTTACGTCGCAGTCCAAGACATCAGGATCGGT |
| core_20 | AGGCTTCGTCACTGACAGAGTATAGGTAACAC |
| core_21 | GATAGGCCACACTTTCCTTTTGTTCTACTGGA |
| core_22 | CCAACTGCTGTTGACGTCCCAGGGCTCGCGCCGA |
| core_23 | TTATGCCAGCATCCTTTTAGAGCTGCATTCCC |
| core_24 | AACCGGATTGCAAGTAGTCCGCCTAGACCTACCA |
| core_25 | CTAATCATTCGCTTTATCACCATCTTTCGGGT |
| core_26 | CTTCCCCATCTCTTAGGTACGTTACCGTGAAGAA |
| core_27 | CGCATCGCCTGGCCTGCATAAAATCAGGAATCTT |
| core_28 | GAATAGGTCAAGGTCATACTCAAATCCATCCG |
| core_29 | CCTTTCCGCCAAGTGCAGGCGTTCAGCCATAA |
| core_30 | CTCCACGCCTGCCTACTCTGTGGATTTTCACGGG |
| core_31 | TTTCGACCCCGGAACCTGATTCCGACTTCCATGG |
| core_32 | CAAGGCCCTCACTACCCAGATTCCCCTTGTCCGT |
| core_33 | ACACCCTCTATGTCTCTACGTGGAAAATGAATTC |
| core_34 | ATGACATTGCAATTCGTCGCGGCAATGCCTGA |
| core_35 | ATCGTAACAACAAGGCT |
| core_36 | CCTCATAAAACTGATACTTCGGCCGGTGAGTTGT |
| core_37 | CCTGCTGCGGTTATCACCGTCACAAGCGCACC |
| core_38 | AGCTCCCACTTATTCTCGACGGTCTACAAGAG |
| core_39 | ATTTATCCCCACGCAAA |
| core_40 | CGGGCGGCATATAACCAGCGAGGCCCCAACCTAC |
| core_41 | GGTTCGATTAGTCTTTCATATCAACCCTGACGGT |
| core_42 | TCGCTAGATAGTAGATATCTGCGTTATCGTTTAA |
| core_43 | GGTCCGTGTTTCAAGAGAAGGCACTTTACAAA |
| core_44 | GCTGTTCACCTTGGAGATGAATTTCTTCACTTTG |
| core_45 | TCAGTCTTCAAAGTTCTGTGATAAGCTGTTAAGA |
| core_46 | GACATCGAAGAATCAAAACTGTTCCTCTCGTACT |
| core_47 | GAACCAGCTACTAGATTGCACGTCAGAACCGC |
| core_48 | GGATCTATTTTGCCGAATAAACCAATTCCGGG |
| core_49 | ATGGTTCAGCGACGCCAATCACCGCGTTCTAG |
| core_50 | GGTAACTTTTCTGGCACTCCAGCGGATGGTAGCT |
| core_51 | GGAACCTTTCCCCACTCCACCGTCCGGCTGTC |
| core_52 | ATGGTTTGTATTCACACATTGTGTGGAGCAAAGA |
| core_53 | CACTAGAGGCCGTTCGTTTCAGGGCTAGTTCA |
| core_54 | GCGTCACTAATTAGATTTTACCCGCGCTTGGT |
| core_55 | TATGATAGGAAGAGCCGACCTTGAATGCTAGA |
| core_56 | GGCCTTTGAAACGGAGCTTAACTCTACGTTCG |
| core_57 | TACCTGATTTGAGGTCACACCATGTCTGATCAAA |
| core_58 | CTTCCCTTATCTACATTGCAGAAATCACATTGCG |
| core_59 | CCAGCAAGCACCCAAGG |
| core_60 | TACCCCGTTGTACATCT |
| core_61 | CCTTATCCCGAAGTTACACCATCGCAATGCTATG |
| core_62 | TCCCTTGGCTGTGGTTTCAACATCACTTTCTG |
| core_63 | CCTACCCACAAGGAGCAAAATTCGACGATCGATT |
| core_64 | AAGCAGTCCACAAGCACTCGTTAATTGTAGCAAG |
| core_65 | AGCCAGTTATCCCTGTCAGATGTGCCGCCCCA |
| core_66 | AACTGATGCTGGCCCAGTACGAGCCTCCACCAGA |
| core_67 | GTAACAAGGACTTCTTCGATTGTGCACCTCTT |
| core_68 | ACCGAATTCTGCTTCGGGCGGTAACATTCATCAG |
| core_69 | TTTTACACCCAAAAATGGCCCACTAAGTTCATCC |
| core_70 | AAGAAACTAGTTCCCTATTAGTGGGTTGTCTCAC |
| core_71 | AGGCCGTCTTATTTCACTGGCGCCGAAATAAGTA |
| core_72 | ATTACAACTCGGGCACCTGGAATTTACCACCCAC |
| core_73 | GTCTTCTTTCCCCGCTTTTTAATTAGACAGTC |
| core_74 | CAGAGCAAGCCCCACCTCCATATCCAGGTTCC |
| core_75 | GAACAATCCAACGCTTCAGCTCCGCTTCATTG |
| core_76 | GTACGACCTGGCATGAACATAGTTACTCCCGCCG |
| core_77 | AAGTCGTATACAAATGCCAATTATCCGAATGA |
| core_78 | ATTCTATCAACTAGAGTGGCCTCTTCCAGCCA |
| core_79 | GCCCGCTGCGTCTGACCGTCTCCACATTCAGT |
| core_80 | ACTCTACTGCTTACAAAAGTTCAATTACTATT |
| core_81 | CTAAGGCAATCCCGGTTGTTCTTTTCATCTTTCC |
| core_82 | GGTTTCTTTTCCTCCG |
| core_83 | TCACAATGTCAAACTAACCAGTTCTAAGTTGA |
| core_84 | AAGCAATGTCGCTATGACAATGTCTTCAACCC |
| core_85 | TCAGTCCCAGCTGGCAAATATTTAGCTTTAGA |
| core_86 | AGCTACATTCCTATGGACCCTATTCAGGCATAGT |
| core_87 | ACATATTTAAAGTTTGATAGATGAACTAACACCT |
| core_88 | GTTCAGCGGGTACTCC |
| core_89 | ACCGTTGCTAGCCTGCT |
| core_90 | CTCTAGCCTCAAATTCAGCCCCCCTTAGGACA |
| core_91 | ACCCGACCTTACGGTCTGGAGCCAGCAAAGGTGC |
| core_92 | GACCCTTAGAGCCAATAGAAAAGATAACTCCT |
| core_93 | GAGGGAAACTTCGGCAGGCTTGAGCGCCATCCAT |
| core_94 | GACAAGGAACGGCCCCAAAACAACTCGACTCTTC |
| core_95 | TGAAATGCGAGATTCCTGCCCTTCCCTTTCAA |
| core_96 | TTTATCCTGCCACCAATCACTCTCTTTTCAAA |
| core_97 | GGCTTAATCTCAGCAGTAGGGTAAAACTAACC |
| core_98 | CTTATTGATATGCTTAACAATTTCACGTACTTTT |
| core_99 | CGTTAATCCATTCATGCGATTTCTGTTCTCCATG |
| core_100 | GAAGGTACCAGATTTC |
| core_101 | CTCCTCGCCTAGACGTTAGACTCCTTCGTATGGG |
| core_102 | ATCGACTAACCCACGTTTTGTGGTGTCTGATG |
| edge_01 | ttttttCGATGGAGTTCTtttttt |
| edge_02 | ttttttGAGAGTAACTATtttttt |
| edge_03 | ttttttGCTCACTAAAGGtttttt |
| edge_04 | ttttttCTCGCAACACGGtttttt |
| edge_05 | ttttttATCTATTGAAAAtttttt |
| edge_06 | ttttttTCCTTCCGTCAGtttttt |
| edge_07 | ttttttTAGTGGACAACAtttttt |
| edge_08 | ttttttATACCCGAGGGCtttttt |
| edge_09 | ttttttAGGCACCCCTTTtttttt |
| edge_10 | ttttttGATTCTCACCCTCTATGACGTCCGAACCGCA |
| edge_11 | ttttttGCTTACCAAACAtttttt |
| edge_12 | ttttttGGCATCGCCCCTtttttt |
| edge_13 | ttttttTCAAAACCTTAAtttttt |
| edge_14 | ACAAGGACGCCTTATTCGTATCCtttttt |
| edge_015 | AACCCAtttttt |
| edge_016 | ACAAAAAAC |

DNA staples of 26S+18S (*S. cerevisiae*) rRNA:DNA Combined rectangles (11-fold geometry)

| Name | Sequence |
| --- | --- |
| core_01 | ACAGGCTATAATACTTGATGCTGGCCCAGTGA |
| core_08 | TTCAATTAAGTAACAAGACCCCGGAACCTCTA |
| core_03 | GCTCCGTCAGTGTAGCCCCTTCCGTCAATTCC |
| core_04 | TACCCGTTGAAACCATGAGGACTCAAGGTTAGCC |
| core_05 | ACAAGGACGCCTTATTTCAGACAGCCGCAAAA |
| core_06 | ACATCACTTTCTGACCACCAGCTCCGCTTCATTG |
| core_07 | CAATTGAATACTGATGAGAAGGAAAGGCCCCG |
| core_08 | GACGAGGCATTTGGCTCCTTATCTACATTATT |
| core_09 | CTTCGATCCCCTAACTATATACGCTATTGGAG |
| core_10 | TCACAAGCGCACCGGAGCTATCAACTAGAGGCTG |
| core_11 | ACCGAGGCAAGCTACATTAGGTCAAGGTCATTTC |
| core_12 | AACTCTACGTTCGGTTCTTGAATATTTGCTACTA |
| core_13 | AAGAATTTCACCTCTGAGTTTCCCCGTGTTGAGT |
| core_14 | TTTCGATGGTGGCCTGCGCGACGGTCTACAAGAG |
| core_15 | TTTATGGTTAAGACTAGCTGGCACCAGACTTG |
| core_16 | GGGCCCTGTATCGTTATCTGGAATTACCGCGGCT |
| core_17 | TAAGTCGTATACAAATGGAGCCATTCGCAGTTTC |
| core_18 | AACGCTTGACTGCCACCTGCCAAGCCCGTTCC |
| core_19 | CGTTATCGTTTAACAGGGATCAGCCCCGAATG |
| core_20 | GGGTACTCCTACCTGATATCCTGCCACCAAAACT |
| core_21 | TTTCTTCACTTTGACAGCCCTCACTACCCGAC |
| core_22 | CCGTCCGGCTGTCTAGACTTCGTCACTGACCTCC |
| core_23 | AACCATCGCCAGCACAAGCAATACGCCTGCTTTG |
| core_24 | GGGGGCTTTTACCCTTAATAAGTAAAGAAACT |
| core_25 | CCAGCAAAGGTGCTGGACGCCTGCCTACTCGT |
| core_26 | CGCGTCACTAATTAGATTTCTTTCCCCGCTGATT |
| core_27 | GGACTTCTTACATATTTCTTTGAAACGGAGCTTC |
| core_28 | AAACAACTTCAAATCCATCCGAAGACTTTCGGGT |
| core_29 | ACCAAGACCTCTCGGCCGACCATACTCCCCCC |
| core_30 | AGCATCCTTGACTTACGCTTCTGCTATCCTGAGG |
| core_31 | TAATTAGACAGTCAGATGGACCTTGAATGCTAGA |
| core_32 | GGATTTTCACGGGCCGCCTGACGGTAGAGTAT |
| core_33 | TTCAGAGCACTGGGCAGATAAAGGTAGTGGTATT |
| core_34 | AAAACGTCCTTGGCAATCAACTACGAGCTTTT |
| core_35 | TAATTTGCGCGCCTGCCGATAACT |
| core_36 | CAGCTGGCAGTATTCCCATCATTCGCTTTACCTC |
| core_37 | AACCCTTATTCCCCGTTTTAAGCA |
| core_38 | TGCCTTCCTTGGATGTGCCAACCGGGCCCAAAGT |
| core_39 | CCCCCGACCGTCCCTATTCTGTCAATCCTTATTG |
| core_40 | GCCGGTGAGTTGTTACAGGCATAGTTCACCAT |
| core_41 | GGTAACTTTTCTGGCACAAGCTCAACAGGGTC |
| core_42 | GTTACTCCCGCCGTTTATCCCGAAGTTACGGA |
| core_43 | TAAGAAGTGGATAACCAAGAAAGAGCTCTCAA |
| core_44 | ATGGTTTGTATTCACATCACTGGCGCCGAAGC |
| core_45 | CACCGCGTTCTAGCATGCGAAAAGTTATTATGAA |
| core_46 | TCTTAACCGGATTCCCCCCATCTCTTAGGATC |
| core_47 | TCTGTTCTCCATGAGCACGTGGAAAATGAATT |
| core_48 | AATGACATTGCAATTCGAGGAACTATCAAATAAA |
| core_49 | TACTCTACTGCTTACAAATACTTAGACATGCATG |
| core_50 | TTGTGCACCTCTTGCGACTGGCTTCACCCTATTC |
| core_51 | TCATTCAAATGTCCACGGACTAACCCACGTCCAA |
| core_52 | CCCCCTTAGGACATCTGTTCGCGGCAATGCCTGA |
| core_53 | GTAGGCCACTATCCTACTAATAAATACATCTC |
| core_54 | ACACTCCTTAGCGGATTCAGGGCATCATATCAAC |
| core_55 | TAGAACCAAACGTCCTAACACTCTAATTTTTT |
| core_56 | ACCCGCGCTTGGTTGAATCCCACTTATTCTACAC |
| core_57 | GTTTCAAGACGGGCGGCCCAGCTACTAGATGGTT |
| core_58 | CTGAAAATCAAAATCAATAAGTTCAATTACTATT |
| core_59 | ATATAACCATTATGCCCTGATCAAATGCCCTT |
| core_60 | TCGCAGTCCTCAGTCCCCCACAAGGAGCAGAG |
| core_61 | TGTTCCTCATCCCTAGTCGGCATAGCGAGTGGGT |
| core_62 | GCCTTTCCGCCAAGTGCCTCTAGAATTACCACAG |
| core_63 | AGACTCCTTGGTCCGTCGTACTTTTTCACTCT |
| core_64 | AGTCTTCAATAAATCCGAAAAAATCGGACCGG |
| core_65 | GGGCTCGCGCCGACGTTCTTCAAAGTTCTCAT |
| core_66 | ACTACTGGACATTGTACTCATTCCACCCTCCAAT |
| core_67 | ACACTCGCATAGACGTTCCTATACCCAAATTCGA |
| core_68 | CACTTTCATTACGCGTACAGAACCGCTACGAGCC |
| core_69 | TATGGTTCAGCGACGCCTTCCAAAGGGTCGAGAT |
| core_70 | TCTGCTTACCAAAAATGACCTTTCCCCACTTCAG |
| core_71 | TAATCATTACGATGGTTTCGCCAAGAGCCACA |
| core_72 | CCTCTTCCAGCCATAAGTCTATTTTGCCGACTTC |
| core_73 | ATTACAAGACCCGAATGCTTAATC |
| core_74 | CTCCACATTCAGTTACGAGCAAGCCCCACCAAGC |
| core_75 | GGTTTCGCAAAGCAATGTCGCTATGGATAGGAAG |
| core_76 | ATGTGCCGCCCCAGCCAGAGGCGTTCAGCCATAA |
| core_77 | AAACTTCCATCGGCTTTGTCTGGACCTGGTGA |
| core_78 | CGTATCCATCTATATTGAGACATTGATTTTTTAT |
| core_79 | TAAGCTGTTAAGAAGACCACCAAGATCTGCAC |
| core_80 | AGTTCTAAGTTGATCGTGACAATGTCTTCAACCC |
| core_81 | ACCTTAAGAGAGTCATACAATGTCAAACTAGAGT |
| core_82 | GCCCACTAAAAGCTCTATAAAACTGATACGAG |
| core_83 | ATCAGTACAGGGACAGTGGGAATCTCTTGGCTGT |
| core_84 | TTCGTTCTTGATTAATGTTTAAGTTTCAGCCTTG |
| core_85 | CCTGAATTAGGATTGGGTAACTGCAACAACTTTA |
| core_86 | GCTTGAGCAAACTATTCCTTCCTGTGCTGCGGTT |
| core_87 | AAAGATAACTCCTCCCACGCTGCGTCTGACCAAG |
| core_88 | CGTTAATCCATTCATGTTCACCTTGGAGACCT |
| core_89 | CGAGGGACTAAAGGATCCTCTATGTCTCTTCA |
| core_90 | ATCCCGCATCGCCAGTGAAACTTCGGCAGGAA |
| core_91 | TTACCGTGAAGAATCCCTGCTGTTGACGTGGA |
| core_92 | ATGCTTTCGCAGTAGTTCTCCACTCCTGGTGGTG |
| core_93 | TTGTTCTACTGGAGATTACCAATTATCCGAATGA |
| core_94 | TGGGTTTTACACCCAACATCTTTCCATCACTG |
| core_95 | CCTAGAAACCAACAAAACCACCACCCACAAAATC |
| core_96 | CCATCGAAAGTTGATAGCAAAGTAAAAGTCCTGG |
| core_97 | AAGCCAGTTATCCCTGTCAATCCAACGCTTACCG |
| core_98 | CTCCCTCTCCGGAATCGTTGGAAATCCAGTACAC |
| core_99 | AAATCACATTGCGTCAAGTCCACAAGCACGCC |
| core_100 | AACCAATTCCGGGGTGACCTTAGAGCCAATCCTT |
| core_101 | CGTCTAAGGGCATCACCAAATTAAGCCGCAGG |
| core_102 | TACCCCGTTGTACATCCCTATTAGTGGGTGAA |
| core_103 | CATCAGGATCGGTCGAACCACCCACTTAGAGC |
| core_104 | TGAACTAACACCTTTTCGATCGATTTGCACGT |
| core_105 | CGACGGTATCTGATCATAGAACCCAAAGACTTTG |
| core_106 | ATATCCAGGTTCCGGAATACAACAAGGCACGCAA |
| core_107 | ATTCTATTATTCCATGCCAAATCACTCCACCAAC |
| core_108 | TCCCCTTGTCCGTACCACCTACCAAGGCCGTC |
| core_109 | GGCCCCAACCTACGTTTCTCTCGCCAATATTT |
| core_110 | CCAGCAAGCACCCAAGGCGGTAACATTCATCA |
| core_111 | TCTATCCCCAGCACGAATTTCTCGTAAGGTGC |
| core_112 | GTGGTGTCTGATGAGCGTAGAGGCCGTTCGACCC |
| core_113 | CCTCTAGCCTCAAATTCAAACCCAGCTCACGTTC |
| core_114 | CGATAGGCCACACTTTCGTAGGGTAAAACTAACC |
| core_115 | AGCCGACAAGGCTTAATCTCAGCAGAAGCATATG |
| core_116 | ACCCCATCTCCGGATAGACCTTACGGTCTAGG |
| core_117 | CCCAACAGCAGGGCTAGTTCATTCGAGGTAACAC |
| core_118 | GGCAGAAATTTGAATGTCATCAAA |
| coreC_119 | GATTTAATATTTATCCCCACGCAATGTCTCACGACGGTCT |
| coreC_120 | GAGTCCGATGTGGAGCAAAGAAATTCCAGCGGATGGTAGC |
| coreC_121 | TGTATTAGACCGTTGCTAGCCTGCACTGTTCCTCTCGTAC |
| coreC_122 | TTTGAGACATCGTAACAACAAGGCAATTCTGCTTCGGTAT |
| core_123 | TCGCAATGCTATGTTTGTAGTCCGCCTAGCAG |
| core_124 | TGTATTCCGGCACCTTCGATTAGTCTTTCGCC |
| core_125 | CCGACTTCCATGGCCATCCACCAGAGTTTCCT |
| core_126 | GTAGCCGTTTCTCAGGTTATACCATGTAGTAA |
| core_127 | TTATTGTCACTACCTCACTGTATAAATTGCTT |
| edge_01 | ttttttATTCGAGGCCATtttttt |
| edge_02 | ttttttAGCAGATAATATtttttt |
| edge_03 | ttttttCATTTTCTATGCtttttt |
| edge_04 | ttttttCCACCTTAATTGtttttt |
| edge_05 | GGTCTCGTTCGTTATCGCAATTAtttttt |
| edge_06 | ttttttTAGCAAATAAAAtttttt |
| edge_07 | ttttttGTAGATGACCTGtttttt |
| edge_08 | ttttttTGAGAATCCTATtttttt |
| edge_09 | ttttttAATCAATAGATAtttttt |
| edge_10 | ttttttGACTTAAACTCCtttttt |
| edge_11 | ttttttGCGATTGATTCTtttttt |
| edge_12 | ttttttGCATGAGCCATCtttttt |
| edge_13 | CATTAAAAAGCAATAATTACAATGCTTGCGCTTACTAGGAATTCCTCGtttttt |
| edge_14 | ttttttTCTTACCGACTCtttttt |
| edge_15 | ttttttTTGAAGAAACACtttttt |
| edge_16 | ttttttTCAGGCAAAGTTtttttt |
| edge_17 | ttttttCACCCGGTTAAGtttttt |
| edge_18 | ttttttTTCGAAGGCACTTTACAAAGAACTGCATTCCC |
| edge_19 | ttttttGGATTTTTGAGGTCAAAC |
| 18,26Rec_core_scaff_connector | GTATTTCAGGATCAACCAGATAACAAATCAGACAACAATCGAAG |
| CoreLinker01_0[64] | TATGCTTAAGTTCAGC |
| CoreLinker13_19[214] | CGGGCGGTGTGTACAA |
| CoreLinker03_0[82] | ATCCCGGTTGGTTTCT |
| CoreLinker03_19[82] | CGGAAACCTTGTTACG |
| CoreLinker07_0[148] | TCGGGCACCGAAGGTA |
| CoreLinker11_19[247] | AACGCAAGCTGATGAC |
| CoreLinker11_0[214] | ATGACGTCCTGTTCCA |
| CoreLinker05_0[115] | GCTTTTGCCGCTTCAC |
| CoreLinker09_0[81] | ACGGCCCCAAAGTTGC |
| CoreLinker09_19[181] | GGCCTCACTAAGCCAT |
| CoreLinker05_19[115] | ATGACCAAGTTTGTCC |
| CoreLinker07_16[148] | ATGGAGTTGCCCCCTT |
| CoreLinker01_17[64] | TAATGATCCCTATTTAGTAGGTTAATAAGAACGGCCATGCA |
| CoreLinker13_0[247] | CGCACTCCTCGCCACA |
| CoreLinker12_18[246] | CGGAGTTTCACAAGATTAGGGCAGGGACGTAATC |
| CoreLinker02_18[81] | AGCAAATGCTAGCACCATTCCGCAGGTTCACCTA |
| CoreLinker06_18[147] | AGACCTGTTATTGCCTCAAATTCTCCGCTCTGAG |
| CoreLinkr12_1[246] | CGGGATTCTCACCCTCTAGCTTTAGATGGAATTT |
| CoreLinker08_1[180] | CCTCTCCAAATTACAACCTTTTCAAAGTTCTTTT |
| CoreLinker10_18[213] | CAAGGTTAGACTCGCTGTCAATCGGTACTAGCGA |
| CoreLinker02_1[81] | TTTCCTCCGCTTATTGAAATGCGAGATTCCCCTA |
| CoreLinker06_1[147] | CCAGATTTCAAATTTGACCCTTTCAACAATTTCA |
| CoreLinker10_1[213] | AGGAACATAGACAAGGATACTTGTTCGCTATCGG |
| CoreLinker04_1[114] | TCGCCGTTACTAAGGCAGGCACAAAACACCATGT |
| CoreLinker04_18[114] | GAAACCGATAGTCCCTCACTTTTAGTTCCTCTAA |
| CoreLinker08_18[180] | GCGCGTGCGGCCCAGAACTCTAAGCAGATCCTGA |

DNA staples of 26S (*S. cerevisiae*) 2D rRNA:DNA Cuboctahedrons (11-fold geometry)

| Name | Sequence |
| --- | --- |
| st3 | CAAAATGACATTGCAATTCGCCTCAAACACAAATCAGACAACAA |
| st5 | CTTAGAGGCGTTTTTTTTCAGCCATAATCCAGCGGATGG |
| st9 | ATCAGTAGGGTTTTTTTAAAACTAACCTCCTATTAGTGGTTTTTTGTGAAC |
| st10 | AATCCGAAGAGCCGACTTTTTTATCGAAGAATCAAAAAGCAATGTCCCTGTGGTA |
| st11 | ACTTTTCTGGCTTTTTTACCTCTAGCCTCAAATTCCGAG |
| st15 | TTCTCCATGAGTTTTTTCCCCCCTTAGGACATCTGCGTTCCCACCTGACA |
| st16 | ATGTCTTCAACTTTTTTCCGGATCAGCCCCGAATGGGAC |
| st20 | ACTTATTCTACTTTTTTACCCTCTATGTACAGGGTCTTCTTTTTTTTTCCC |
| st21 | CGCTGTCGCTAGATAGTTTTTTTAGATAGGGACAGTGGGAATCTACGAGGCATTT |
| st22 | GGCTACCTTAATTTTTTGAGAGTCATAGTTACTCCCGCC |
| st24 | GACATTCAGAGTTTTTTCACTGGGCAGAAATCACATTGCTTTTAATTAGA |
| st25 | CAGTCAGATTCTTTTTTCCCTTGTCCGTACCAGTTCTAA |
| st27 | AAGAGACCTACTTTTTTCAAGGCCGTCTAGCAAGCCCCATTTTTTCCAAGC |
| st28 | AGTCCTCACTACCCGATTTTTTCCCTTAGAGCCAATCCTTATCCTCTACATTATT |
| st29 | CTATCAACTAGTTTTTTAGGCTGTTCACCTTGGAGACCT |
| st33 | TTCCAGCCATATTTTTTAGACCCCATCTCCGGATAAACCTAACTCCTCCC |
| st34 | AGGGCTCGCGCTTTTTTCGACGTCTCCACATTCAGTTAC |
| st38 | ATCTCTTAGGATTTTTTTCGACTAACCCCCACTTCAGTCTTTTTTTTCAAA |
| st39 | GTTCTGCACTAGAGGCTTTTTTCGTTCGACCCGACCTTACGGTCCGTCAGGGCAT |
| st40 | CATATCAACCCTTTTTTTGACGGTAGAGTATAGGTAACA |
| st44 | CTAGATGAACTTTTTTTAACACCTTTTGTGGTGTCTGATGTTCATCCCGC |
| st45 | ATCGCCAGTTCTTTTTTTGCTTACCAAAAATGGCCCACT |
| st49 | CTCTAATCATTTTTTTTCGCTTTACCTCGGAAACTTCGGTTTTTTCAGGAA |
| st50 | CCAGCTACCCAAATTCTTTTTTGACGATCGATTTGCACGTCAGATTCACCCTATT |
| st51 | CAGGCATAGTTTTTTTTCACCATCTTTCGGGTCCCAACA |
| st55 | GTTTTACACCCTTTTTTAAACACTCGCATAGACGTTAGAAACCATTATGC |
| st56 | CAGCATCCTTGTTTTTTACTTACGTCGCGGCTATAATACTTTTTTTTACCGAGGCA |
| st57 | AGCTACATTCCCCCAGTGAAATGCGAGATTCCCTTTTTTCTACCCACAAG |
| st61 | TTGTTCGCTATTTTTTTCGGTCTCTCGCACTTAGAGCTGTTTTTTCATTCC |
| st62 | CAAACCCGCACTCCTCTTTTTTGCCACACGGGATTCTCACCCTCACGGCCCCAAA |
| st63 | GTTGCCCTCTCTTTTTTCAAATTACAACTCGGGCACCGA |
| st4 | CGCCACAAGGATTTTTTCGCCTTATTCGTATCCATCTATATGGATTCTGA |
| st6 | TAGCTTCGCGGCAATGCCTGAT |
| st8 | GTACTAAGTTCAATTACTATTGCGGTAACATTC |
| st12 | GGACTAAAGGATCGATAGGCCA |
| st14 | GCTTTTACCCTTTTGTTCTACTGGAGATTTCTG |
| st17 | CTTGAATGCTAGAACGTGGAAA |
| st19 | AGGTAGTGGTATTTCACTGGCGCCGAAGCTCCC |
| st23 | GTTTACCCGCGCTTGGTTGAATTTCTTCACTTT |
| st26 | GTTGATCGTTAATTGTAGCAAGCGACGGTCTAC |
| st30 | GCTGCGGTTATCAGTACGACCT |
| st32 | AAGCGCACCGGAGCCAGCAAAGGTGCTGGCCTC |
| st35 | GTTACCGTGAAGAATCCATATC |
| st37 | CATAAAATCAGGCCTTTGAAACGGAGCTTCCCC |
| st41 | CGCTTGAGCGCCATCCATTTTC |
| st43 | GGATTCCGACTTCCATGGCCACCGTCCGGCTGT |
| st46 | AAAAGCTCTTCATTCAAATGTC |
| st48 | GAGAATAGGTCAAGGTCATTTCGACCCCGGAAC |
| st52 | GCTATGCTCTTACTCAAATCCA |
| st54 | CCCAACCTACGTTCACTTTCATTACGCGTATGG |
| st58 | GAGCAGAGGGCACAAAACACCATGTCTGATCAA |
| st60 | TTCAAAGTTCTTTTCATCTTTCCATCACTGTAC |
| st64 | AGGTACCAGATTTCAAATTTGA |
| st7 | CAGACAGCCGCAAAAACCAATTTTTTTTATCCGAATGAACTGTTCCTCTC |
| st13 | CACTTTCATGGTTTGTATTCACTTTTTTACTGAAAATCAAAATCAAGGGG |
| st18 | ATGAATTCCAGCTCCGCTTCATTTTTTTTGAATAAGTAAAGAAACTATAA |
| st31 | GGCATGAAAACTATTCCTTCCTTTTTTTGTGGATTTTCACGGGCCGTCAC |
| st36 | CAGGTTCCGGAATCTTAACCGGTTTTTTATTCCCTTTCGATGGTGGCCTG |
| st42 | AGGGCTAGTTCATTCGGCCGGTTTTTTTGAGTTGTTACACACTCCTTAGC |
| st47 | CACGTTCAATTAAGTAACAAGGTTTTTTACTTCTTACATATTTAAAGTTT |
| st53 | TCCGAAGACATCAGGATCGGTCTTTTTTGATTGTGCACCTCTTGCGAGGC |
| st59 | ATGCCCTTCCCTTTCAACAATTTTTTTTTCACGTACTTTTTCACTCTCTT |
| st65 | GCTTTTGCCGCTTCACTCGCCGTTTTTTTTACTAAGGCAATCCCGGTTGG |
| st2 | TCCGCCAAGTGCACTAAGTTCAGCGGGTACTCCTACCTGATTTGAGGAGCAAGCACCCAAGGCCTT |
| st1 | TTTCTTTTCCTCCGCTTTTTTTTATTGATATGCTCGTTGCTAGCCTTTTTTTGCTATGGTTCAGCGA |
| st13-14 | GAGCAAAGAAACCCAGCTCACGTTCGTCTCACGACGGTCTAAATCACCGCGTTCTAGCATTGTGTG |
| st13-10 | CCTGTTCCATGCTTCGGTATGATAGAACGCTTACCGAATTCAGGAACATAGACAAGGATATGACGT |
| st10-12 | AACGCTTGATCCCTTGGCTGTGGTTATTCTGCCAAGCCCGTCTGCCACAAGCCAGTTATCGCTATG |
| st10-8 | ACAAAGAAAACTCGACTCTTCGAAGCGTCACTAATTAGATGCGTTAATCCATTCATGCGGCACTTT |
| st2-5 | CGGATCTATACTACCACCAAGATCTCATTTGAATATTTGCTTTTGCCGACTTCCCTTACGAAGTTA |
| st2-1 | AACCTTTCCACGTCCAACTGCTGTTCTGTTAAGAAGAAAAGAAATTCCGGGGTGATAAGGACGTGG |
| st12-11 | CAAGCTCACTCTTCACAATGTCAACCGCCCCAGCCAAACTCATCGTTTAACAGATGTGACTAGAGT |
| st7-6 | GCACGCAACAAGACGGGCGGCATATCTCCTTGGTCCGTGTTTGTAGTCCGCCTAGCAGACAACAAG |
| st8-7 | TTCCCACAAGTCCTCAGTCCCAGCACCATCGCAATGCTATGGTCAACATCACTTTCTGTGGCAGTA |
| st8-9 | AGCTTTAGACCAAAACTGATGCTGGTATGGATTTATCCTGCCATGGAATTTACCACCCCAATATTT |
| st6-5 | GAGCCTCCACGTCTGACCAAGGCCCACAAGCACGCCCGCTGCCAGAGTTTCCTCTGGCACCGCTAC |
| st5-3 | GTCACTGACTAGTCTTTCGCCCCTATACTAGATGGTTCGATCTCCACGCCTGCCTACTTAGGCTTC |
| st3-4 | ATTCCGGCACTTCTGCTATCCTGAGATAAAACTGATACGAGCCTTAACTCTACGTTCGGAGCGTGT |

DNA staples of 26S (*S. cerevisiae*) rRNA:DNA 3D Cuboctahedrons (11-fold geometry)

| Name | Sequence |
| --- | --- |
| 3D-st1 | ATCGCCAGTTCTTTTTTGCTTACCAAACGCCACAAGGATTTTTCGCCTTATTCG |
| 3D-st2 | TATCCATCTATATGGATTCTGA |
| 3D-st3 | CTTAGAGGCGTTTTTTTCAGCCATAATCCAGCGGATGGCGTCCGGCTGT |
| 3D-st5 | ATTCGGCCGGTTTTTTGAGTTGTTACAAAAAACCAATTTTTTTATCCGAATGAA |
| st9 | ATCAGTAGGGTTTTTTAAAACTAACCTCCTATTAGTGGTTTTTGTGAAC |
| st10 | AATCCGAAGAGCCGACTTTTTATCGAAGAATCAAAAAGCAATGTCCCTGTGGTA |
| 3D-st8 | TTTGTATTCACTTTTTACTGAAAATCAATCTTAACCGGTTTTTATTCCCTTTCG |
| 3D-st10 | AGGGCTCGCGCTTTTTCGACGTCTCCACATTCAGTTACGGAGATTTCTG |
| st15 | TTCTCCATGAGTTTTTCCCCCCTTAGGACATCTGCGTTCCCACCTGACA |
| 3D-st11 | ATGTCTTCAACTTTTTCCGGATCAGCCTTCCAGCCATATTTTTAGACCCCATCT |
| 3D-st13 | CGGGCCGTCACATGAATTCCAGCTCCGCTTCATTTTTTTGAATAAGTAA |
| st20 | ACTTATTCTACTTTTTACCCTCTATGTACAGGGTCTTCTTTTTTTTCCC |
| st21 | CGCTGTCGCTAGATAGTTTTTTAGATAGGGACAGTGGGAATCTACGAGGCATTT |
| st24 | GACATTCAGAGTTTTTCACTGGGCAGAAATCACATTGCTTTTAATTAGA |
| 3D-st16 | CAGTCAGATTCTTTTTCCCTTGTCCGTTATTCCTTCCTTTTTTGTGGATTTTCA |
| st27 | AAGAGACCTACTTTTTCAAGGCCGTCTAGCAAGCCCCATTTTTCCAAGC |
| st28 | AGTCCTCACTACCCGATTTTTCCCTTAGAGCCAATCCTTATCCTCTACATTATT |
| st38 | ATCTCTTAGGATTTTTTCGACTAACCCCCACTTCAGTCTTTTTTTCAAA |
| st39 | GTTCTGCACTAGAGGCTTTTTCGTTCGACCCGACCTTACGGTCCGTCAGGGCAT |
| st44 | CTAGATGAACTTTTTTAACACCTTTTGTGGTGTCTGATGTTCATCCCGC |
| 3D-st22 | TTCACTCGCCGTTTTTTTACTAAGGCAATCCCGGTTGGCACGTTCAATT |
| 3D-st23 | AAGTAACAAGGTTTTTACTTCTTACAT |
| st49 | CTCTAATCATTTTTTTCGCTTTACCTCGGAAACTTCGGTTTTTCAGGAA |
| st50 | CCAGCTACCCAAATTCTTTTTGACGATCGATTTGCACGTCAGATTCACCCTATT |
| 3D-st26 | CAGGATCGGTCTTTTTGATTGTGCACCTTTCAACAATTTTTTTTCACGTACTTT |
| st55 | GTTTTACACCCTTTTTAAACACTCGCATAGACGTTAGAAACCATTATGC |
| st56 | CAGCATCCTTGTTTTTACTTACGTCGCGGCTATAATACTTTTTTTACCGAGGCA |
| st61 | TTGTTCGCTATTTTTTCGGTCTCTCGCACTTAGAGCTGTTTTTCATTCC |
| st62 | CAAACCCGCACTCCTCTTTTTGCCACACGGGATTCTCACCCTCACGGCCCCAAA |
| st1 | ATTGATATGCTCGTTGCTAGCC |
| st3 | CAAAATGACATTGCAATTCGCCTCAAACACAAATCAGACAACAA |
| 3D-st7 | ACTTTTCTGGCTTTTTACCTCTAGCCTCAAATTCCGAGCGGTAACATTC |
| 3D-st15 | GGCTACCTTAATTTTTGAGAGTCATAGTTACTCCCGCCCCGAAGCTCCC |
| 3D-st18 | CTATCAACTAGTTTTTAGGCTGTTCACCTTGGAGACCTCGACGGTCTAC |
| 3D-st19 | CATATCAACCCTTTTTTGACGGTAGAGTATAGGTAACAGGAGCTTCCCC |
| 3D-st21 | TGCTATGGTTCAGCGAAATGGCCCACTAAAAGCTCTTCATTCAAATGTCTTTCTTTTCCTCCGCTT |
| 3D-st25 | CAGGCATAGTTTTTTTCACCATCTTTCGGGTCCCAACAGACCCCGGAAC |
| 3D-st28 | AGCTACATTCCCCCAGTGAAATGCGAGATTCCCTTTTTCTACCCACAAGGAGCAGAGGGCTACGCG  TATGG |
| 3D-st30 | GTTGCCCTCTCTTTTTCAAATTACAACTCGGGCACCGACATCACTGTAC |
| 3D-st4 | CCGACTTCCATGGCCACTAGCTTCGCGGCAATGCCTGATCAGACAGCCGCCACTCCTTAGCGGATT |
| 3D-st6 | AGGCCACACTTTCATGGCTGTTCCTCTCGTACTAAGTTCAATTACTATTGGGACTAAAGGATCGAT |
| 3D-st9 | CATATCCAGGTTCCGGAAAATCAAGGGGGCTTTTACCCTTTTGTTCTACTGTTACCGTGAAGAATC |
| 3D-st12 | ATGCTAGAACGTGGAAAAAGCGCACCGGAGCCAGCAAAGGTGCTGGCCTCCCGAATGGGACCTTGA |
| 3D-st14 | TTGAATTTCTTCACTTTAGAAACTATAAAGGTAGTGGTATTTCACTGGCGGTTTACCCGCGCTTGG |
| 3D-st17 | CGACCTGGCATGAAAACACCAGTTCTAAGTTGATCGTTAATTGTAGCAAGGCTGCGGTTATCAGTA |
| 3D-st20 | ATTTTCAGGGCTAGTTCATGGTGGCCTGCATAAAATCAGGCCTTTGAAACCGCTTGAGCGCCATCC |
| 3D-st24 | AATCCATCCGAAGACATATTTAAAGTTTGAGAATAGGTCAAGGTCATTTCGCTATGCTCTTACTCA |
| 3D-st27 | CCTACGTTCACTTTCATACAAAACACCATGTCTGATCAAATGCCCTTCCCTCTTGCGAGGCCCCAA |
| 3D-st29 | ATTTGAGCTTTTGCCGCTTCACTCTCTTTTCAAAGTTCTTTTCATCTTTCAGGTACCAGATTTCAA |
| st2 | TCCGCCAAGTGCACTAAGTTCAGCGGGTACTCCTACCTGATTTGAGGAGCAAGCACCCAAGGCCTT |
| st13-14 | GAGCAAAGAAACCCAGCTCACGTTCGTCTCACGACGGTCTAAATCACCGCGTTCTAGCATTGTGTG |
| st13-10 | CCTGTTCCATGCTTCGGTATGATAGAACGCTTACCGAATTCAGGAACATAGACAAGGATATGACGT |
| st10-12 | AACGCTTGATCCCTTGGCTGTGGTTATTCTGCCAAGCCCGTCTGCCACAAGCCAGTTATCGCTATG |
| st10-8 | ACAAAGAAAACTCGACTCTTCGAAGCGTCACTAATTAGATGCGTTAATCCATTCATGCGGCACTTT |
| st2-5 | CGGATCTATACTACCACCAAGATCTCATTTGAATATTTGCTTTTGCCGACTTCCCTTACGAAGTTA |
| st2-1 | AACCTTTCCACGTCCAACTGCTGTTCTGTTAAGAAGAAAAGAAATTCCGGGGTGATAAGGACGTGG |
| st12-11 | CAAGCTCACTCTTCACAATGTCAACCGCCCCAGCCAAACTCATCGTTTAACAGATGTGACTAGAGT |
| st7-6 | GCACGCAACAAGACGGGCGGCATATCTCCTTGGTCCGTGTTTGTAGTCCGCCTAGCAGACAACAAG |
| st8-7 | TTCCCACAAGTCCTCAGTCCCAGCACCATCGCAATGCTATGGTCAACATCACTTTCTGTGGCAGTA |
| st8-9 | AGCTTTAGACCAAAACTGATGCTGGTATGGATTTATCCTGCCATGGAATTTACCACCCCAATATTT |
| st6-5 | GAGCCTCCACGTCTGACCAAGGCCCACAAGCACGCCCGCTGCCAGAGTTTCCTCTGGCACCGCTAC |
| st5-3 | GTCACTGACTAGTCTTTCGCCCCTATACTAGATGGTTCGATCTCCACGCCTGCCTACTTAGGCTTC |
| st3-4 | ATTCCGGCACTTCTGCTATCCTGAGATAAAACTGATACGAGCCTTAACTCTACGTTCGGAGCGTGT |

DNA staples of 16S (*E.Coli*) rRNA:DNA rectangles (11-fold geometry)

| Name | Sequence |  |
| --- | --- | --- |
| core_01 | CTACCTACTTCTTTTGATTCACCGTGGCATTC |  |
| core_02 | CCCTCTACGAGACTCAGGTATTAACTTTACTC |  |
| core_03 | AAGCAGCAAGCTGTTTCGGTCCCCCTCTTTGGTC |  |
| core_04 | GGGCCCCCGTCAATTCACTGTCTCACGGTTCCCG |  |
| core_05 | GCGGTCGACTTAACGCTTTACGGCGTGGACTA |  |
| core_06 | CACAAAGTGGTAAGCGCGATTCCGACTTCATG |  |
| core_07 | ATCGAATTAAACCACATTCTGAAAACTTCCGTGG |  |
| core_08 | GTGCAATATTCCCCACTTAACGTCAATGAGCAAA |  |
| core_09 | AAGGGCCATGATGACTTGAGTCGAGTTGCAGACT |  |
| core_10 | GCCTGCGTGCGCTTTACCCTCCAAGTCGACATCG |  |
| core_11 | GCGGCCGTACTCCCCAGTCACAACACGAGCTGAC |  |
| core_12 | TCACCCCAGTCATGAAT |  |
| core_13 | TTCCCGGCCGGACCGCTACAAGGCCCGGGAACGT |  |
| core_14 | GTCTGGACCGTGTCTCATGGCACGGAGTTAGCCG |  |
| core_15 | TCATCCTCTCAGACCAGTAACGCTTGCACCCTCC |  |
| core_16 | GACGTCATCCCCACCTGACAGCCATGCAGCAC |  |
| core_17 | AGCCCGGGGATTTCACACCAGGGTATCTAATCCT |  |
| core_18 | CTGTTACCGTTCGACT |  |
| core_19 | TTTGAGTTTTAACCTTGTTTGCTCCCCACGCT |  |
| core_20 | CAGGCTTGCGCCCATTTTGCGACGTTATGCGG |  |
| core_21 | TGCAGTTCCCAGGTTGGTGCTTCTTCTGCGGG |  |
| core_22 | GTTAGCTCCGGAAGCCAGGTTGCGCTCGTTGCGG |  |
| core_23 | ACGCGGCATGGCTGCATCCTTCCTCCCCGCTGAA |  |
| core_24 | CACCTGGAATTCTACCCCCGCCTTCGCCACCGGT |  |
| core_25 | CCCTCCCGAAGGTTAAG |  |
| core_26 | AGACATTACTCACCCGTTATTAGCTACCGTTTCC |  |
| core_27 | AGCTTGCCAGTATCAGATTCGCACCTGAGCGTCA |  |
| core_28 | GCTCCACCGCTTGTGCGTCTTCGTCCAGGGGG |  |
| core_29 | TGCATGTGTTAGGCCTGCATCTGGGCACATCCGA |  |
| core_30 | GTTCCAGTGTGGCTGGACCTACTAGCTAATCC |  |
| core_31 | TGAGCCATGATCAAACTAGGTGAGCCGTTACCCC |  |
| core_32 | GTGTAGCCCTGGTCGTAAGGCACATTCTCATC |  |
| core_33 | GCTGCCTCCCGTAGGATGGCAAGAGGCCCTAA |  |
| core_34 | GGCAGTCTCCTTTGAGGACTTAACCCAACATT |  |
| core_35 | GCGCCATTGTAGCACGTCGCACTTTATGAGGTCC |  |
| core_36 | TCCTCCAGTTTATCACTTGATCCACGATTACTAG |  |
| core_37 | CGCAGGTTCCCCTACGG |  |
| core_38 | CCGCCAGCGTTCAATC |  |
| core_39 | TCTGACTTAACAAACCGTATTACCGCGGCTGC |  |
| core_40 | TTACCTTGTTACGACTCCAATCCGGACTACGA |  |
| core_41 | CCGCCACTCGTCAGCG |  |
| edge_01 | ttttttttCGGTGTGTGGCAACAAtttttttt |  |
| edge_02 | CAACCCACTCCCATGGTGTGACGGGtttttttt |  |
| edge_03 | ttttttttTCGCTTCTCTTTGTATATGTCAAGACCAGGTAtttttttt |  |
| edge_04 | ttttttttAGGATAAGCGCCTCAAtttttttt |  |
| edge_05 | ttttttttGGGCACAAGCCCAGTAtttttttt |  |
| edge_06 | ttttttttACGCATTTCACCGCTAAGTACTTTACAACCCGtttttttt |  |
| edge_07 | ttttttttAAGGCCTTCTTCATACAGTAGTTATCCCCCTCtttttttt |  |
| edge_08 | ttttttttAGGTTCTTCGCGTTGCATTCCTCCAGATCTCTtttttttt |  |
| edge_09 | ttttttttCGTCGCCTCTTCAATTTttttttt |  |
| edge_10 | tttttttTAAGGAGGTGATCCAACGCTTGCTCTCGCGAGGtttttttt |  |
| edge_11 | ttttttttATTCCGATCTAGGGATtttttttt |  |
| edge_12 | ttttttttCATCAGGCAGTTTCCC |  |

**Supplementary note 4**

**Design schemes of the rRNA:DNA origami structures**

2D rectangles

All 2D rectangles (18S, 26S, combined and 16S rectangles) were designed using caDNAno, with manual adjustment of staple cross over periodicity to fit the A-helix geometry, 11-fold, as described in **Supplementary note 2.**

**
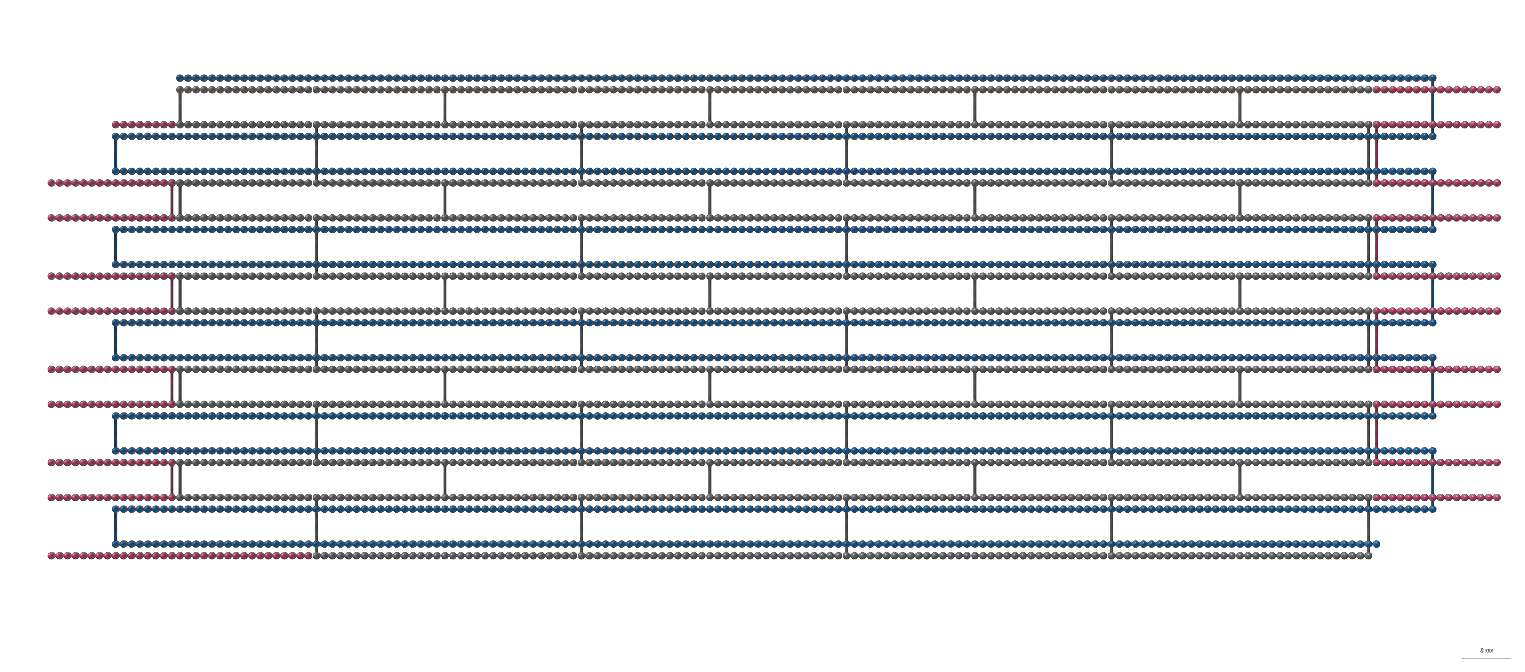
**

2 nm

**
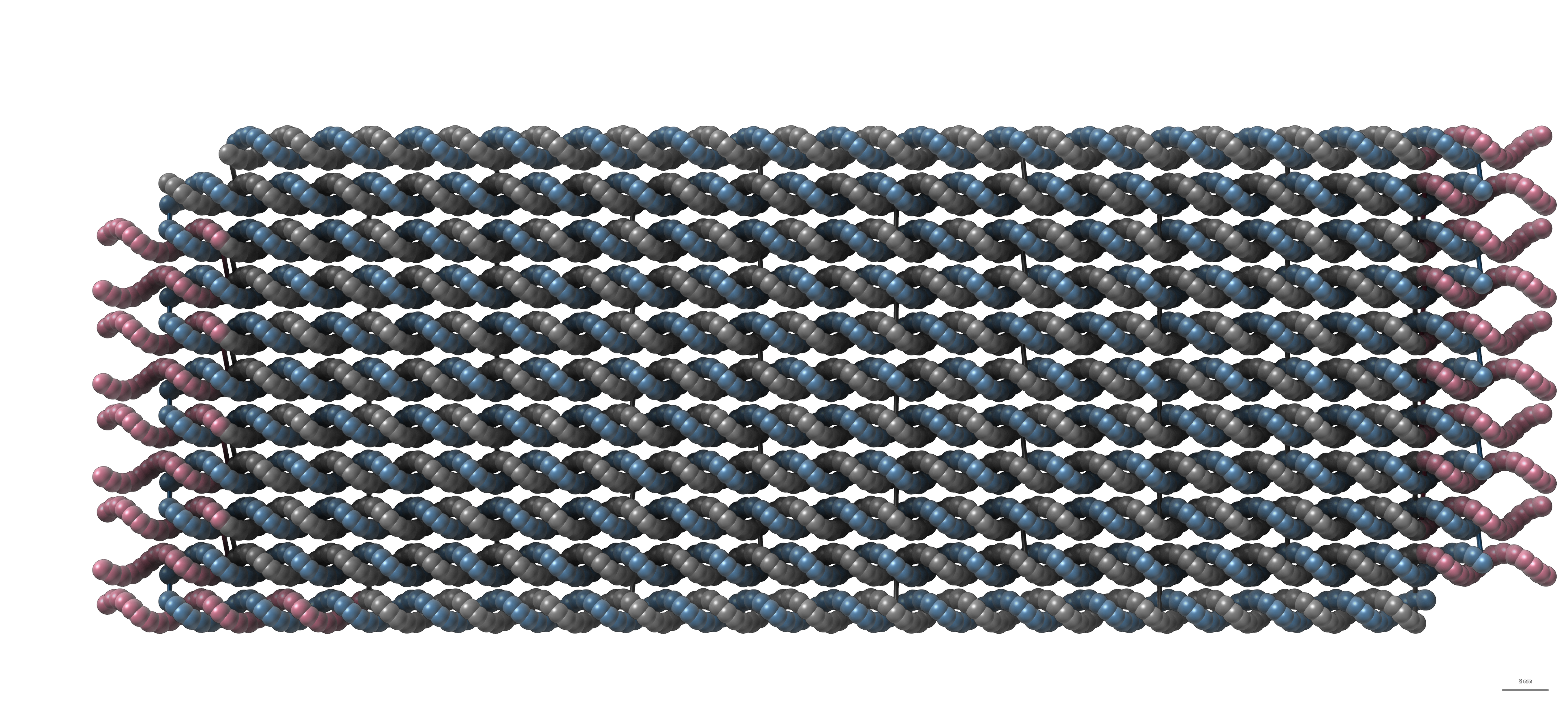
**

2 nm

**Figure S2**. **2D and 3D schematic illustrations of 18S rRNA:DNA rectangle.** 18S rRNA of *S. cerevisiae* (1800b) is used as a scaffold strand (blue). Core and edge DNA staples are colored in gray and fuchsia respectively. 5’ of the scaffold strand is indicated by black arrow.


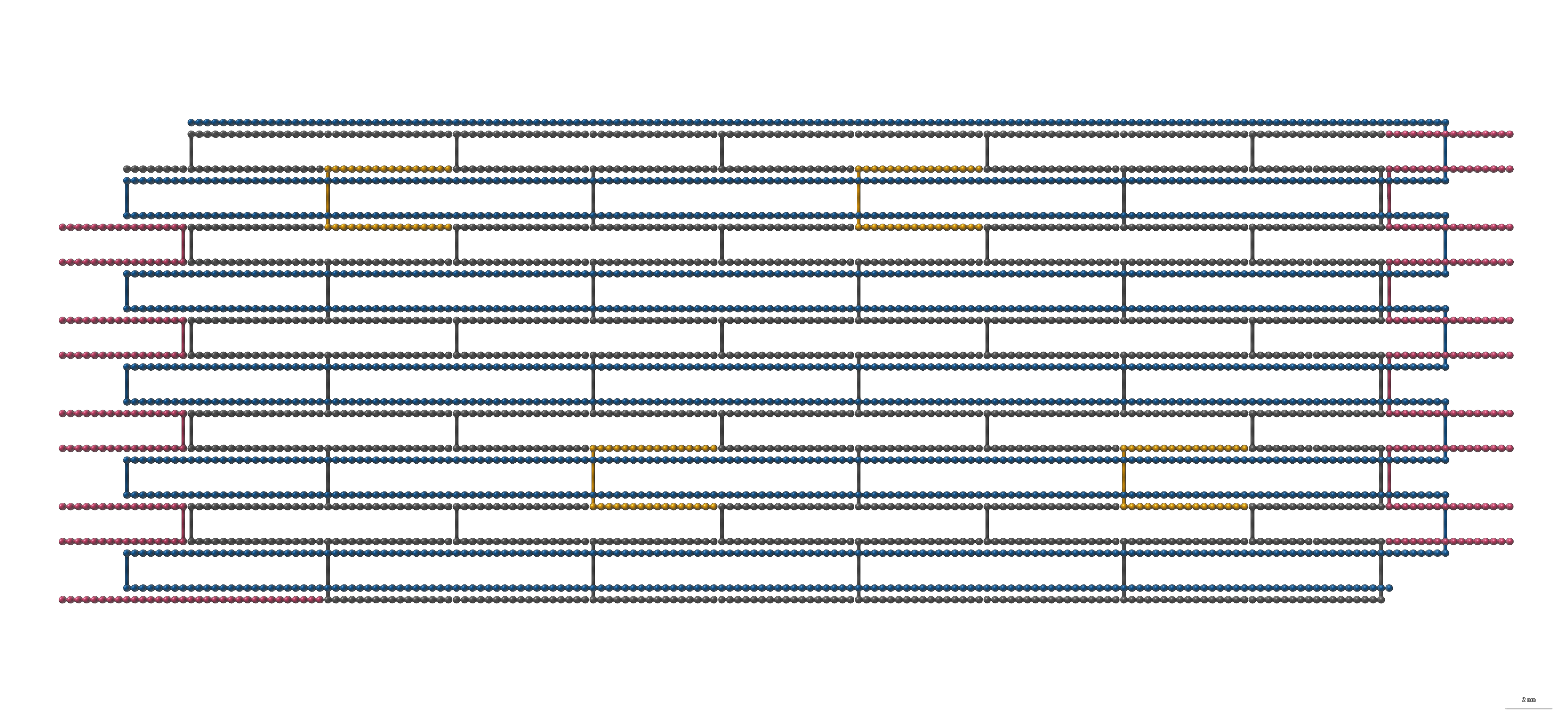


2 nm


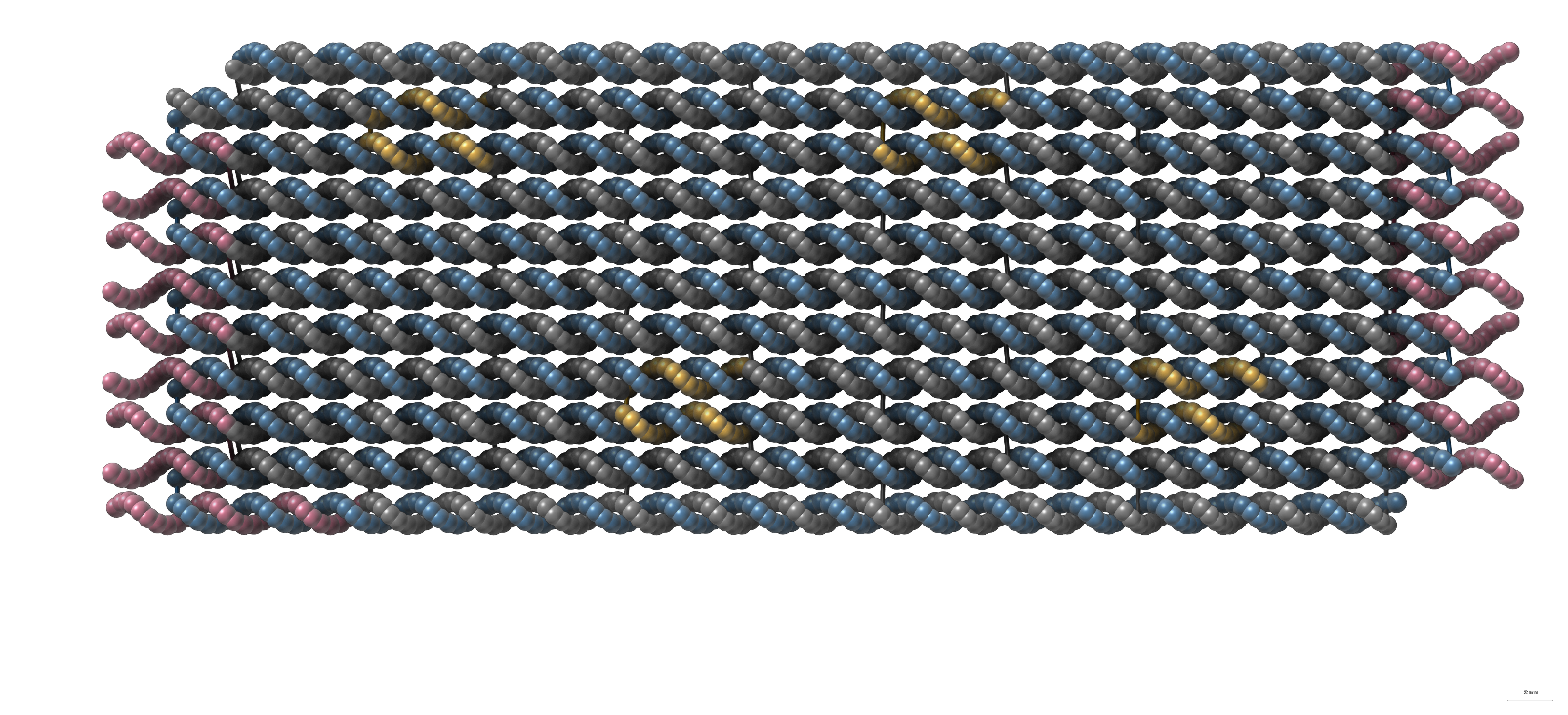


2 nm

**Figure S3. 2D and 3D schematic illustration of 18S rRNA:DNA rectangle with 4 biotin tagged staples.** 18S rRNA of *S. cerevisiae* (1800b) is used as a scaffold strand (blue). Core and edge DNA staples are colored in gray and fuchsia respectively. Biotin was attached to 5’ of the yellow staples, thus the structures comprises four binding sites to Streptavidin. 5’ of the scaffold strand is indicated by black arrow.


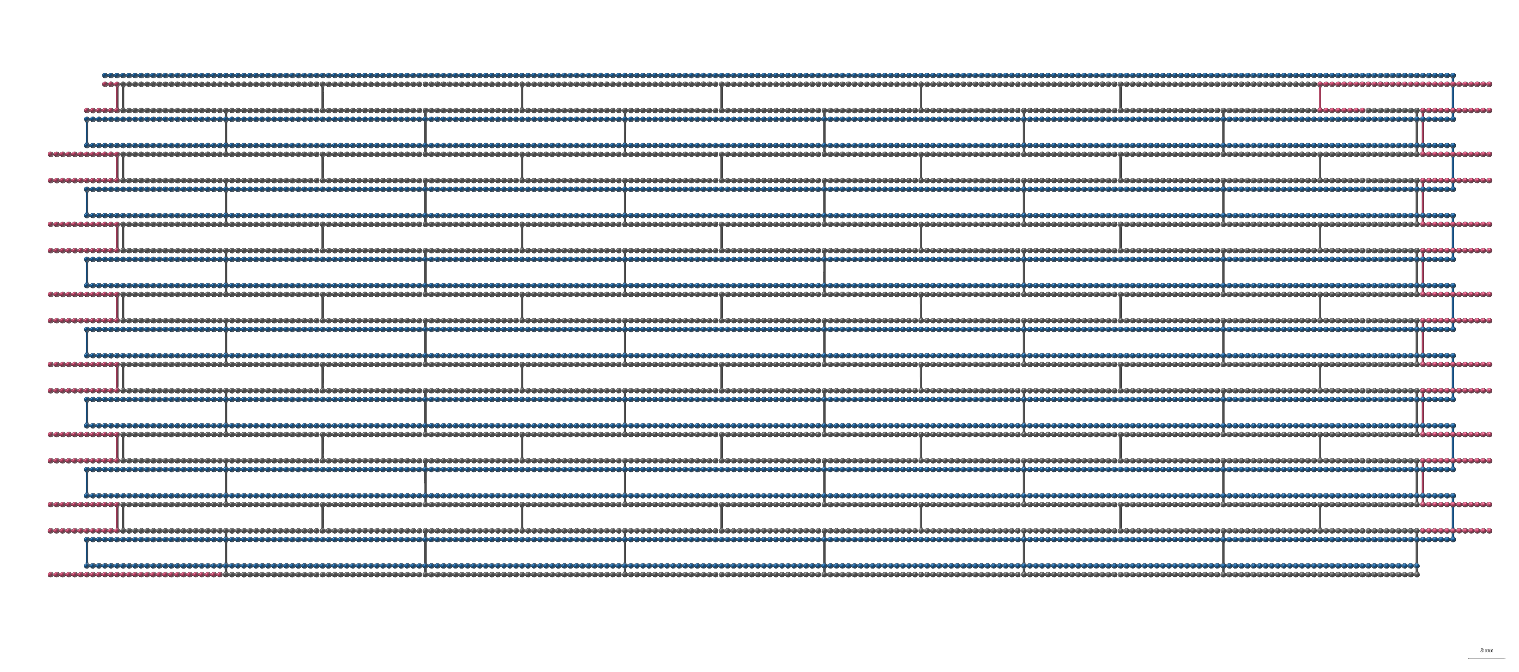


2 nm


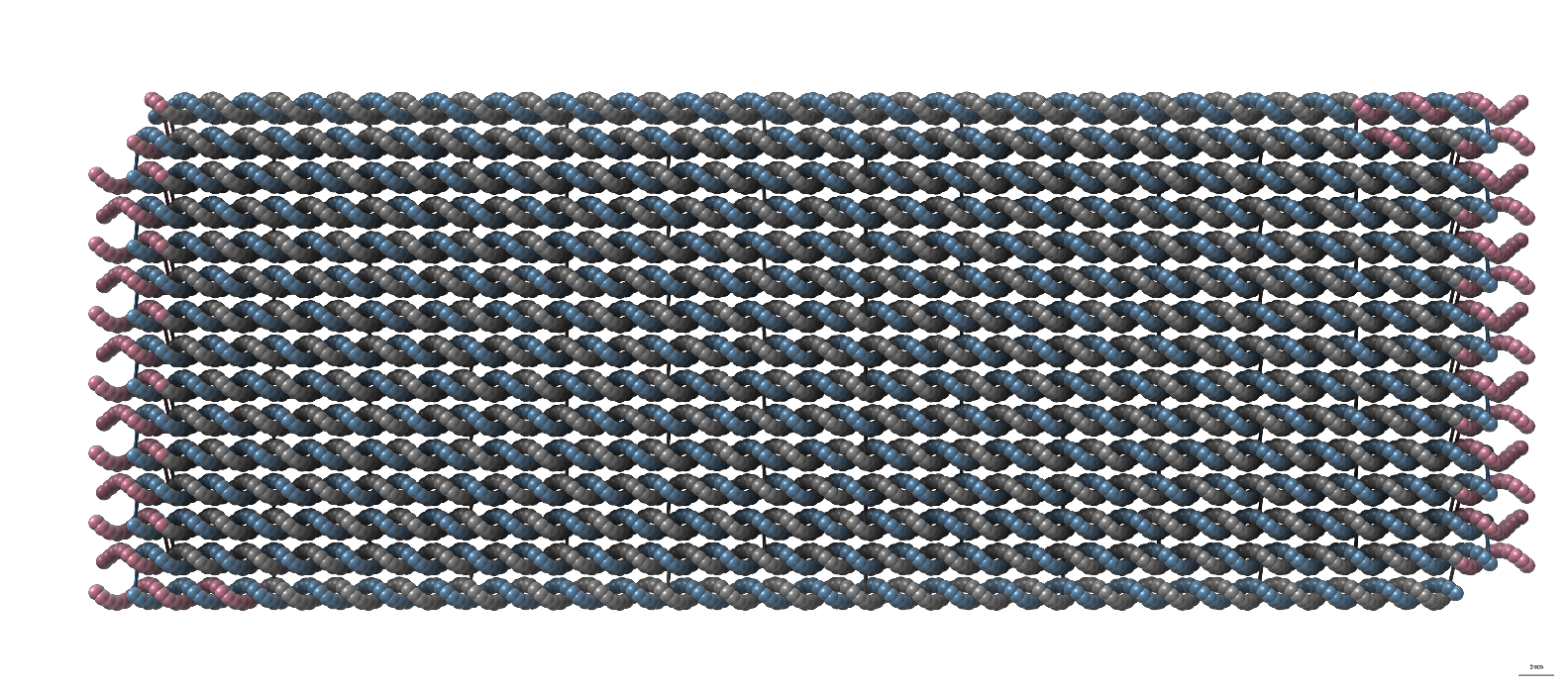


2 nm

15

**Figure S4. 2D and 3D schematic illustration of 26S rRNA:DNA rectangle.** 26S rRNA of *S. cerevisiae* (3396b) is used as a scaffold strand (blue). Core and edge DNA staples are colored in gray and fuchsia respectively. 5’ of the scaffold strand is indicated by black arrow.


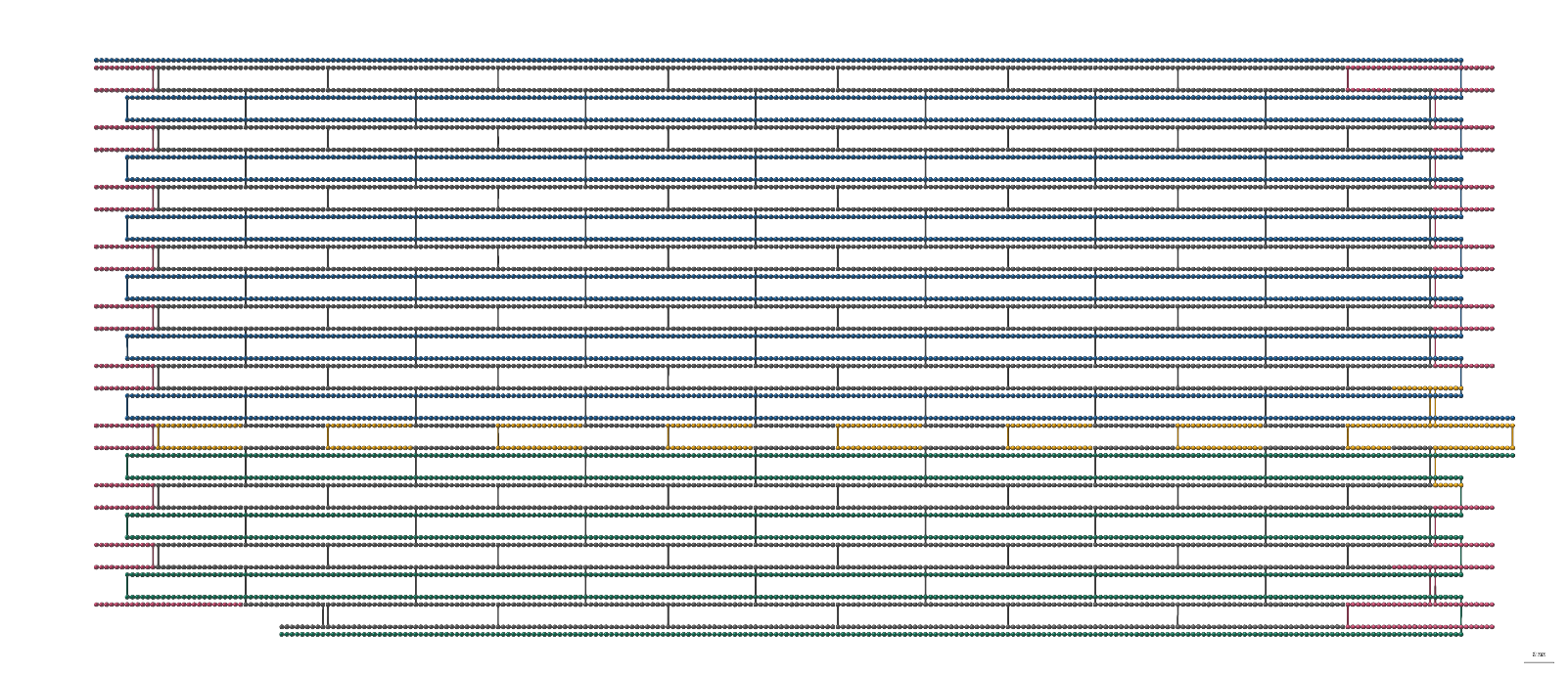


2 nm

15


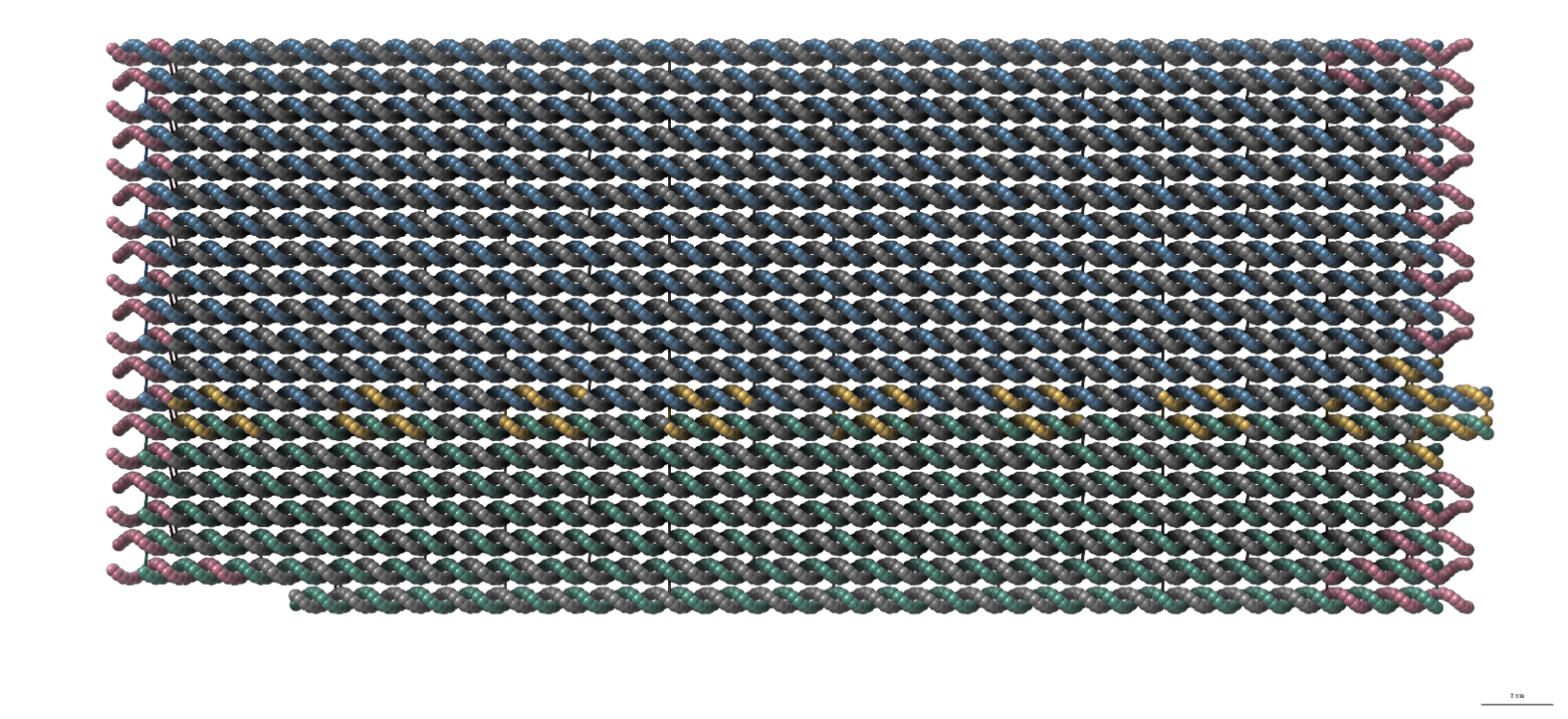


5 nm

15

**Figure S5. 2D and 3D schematic illustration of 26S+18S rRNA:DNA combined rectangle.** 26S and 18S rRNA of *S. cerevisiae* (1800b + 3396b) are used as scaffold strands (18S rRNA – green, 26S rRNA-blue). Core and edge DNA staples are colored in gray and fuchsia respectively. The connector staples that connect 26S rRNA with 18S rRNA are colored in yellow. 5’ of the scaffold strand is indicated by black arrow.


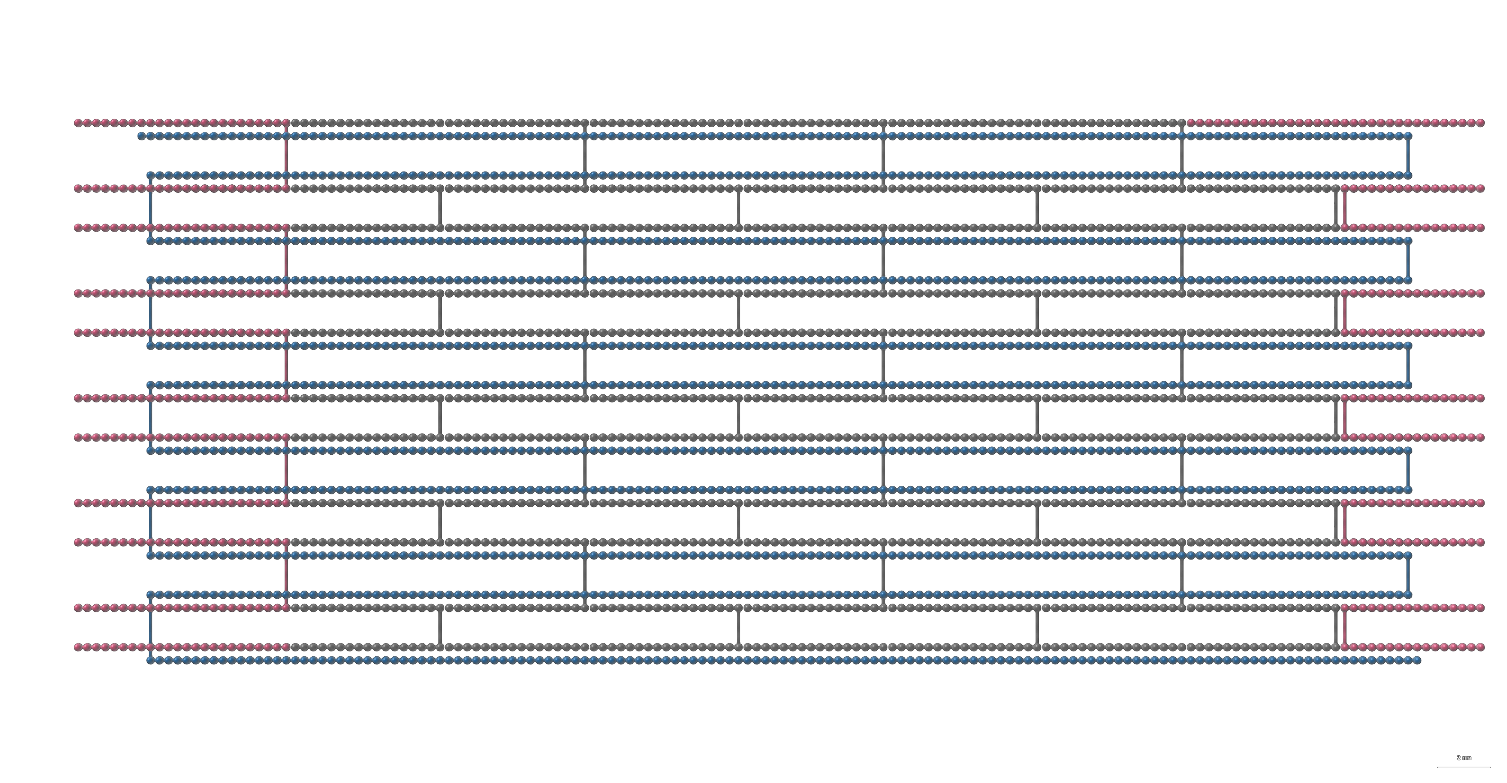


2 nm

15


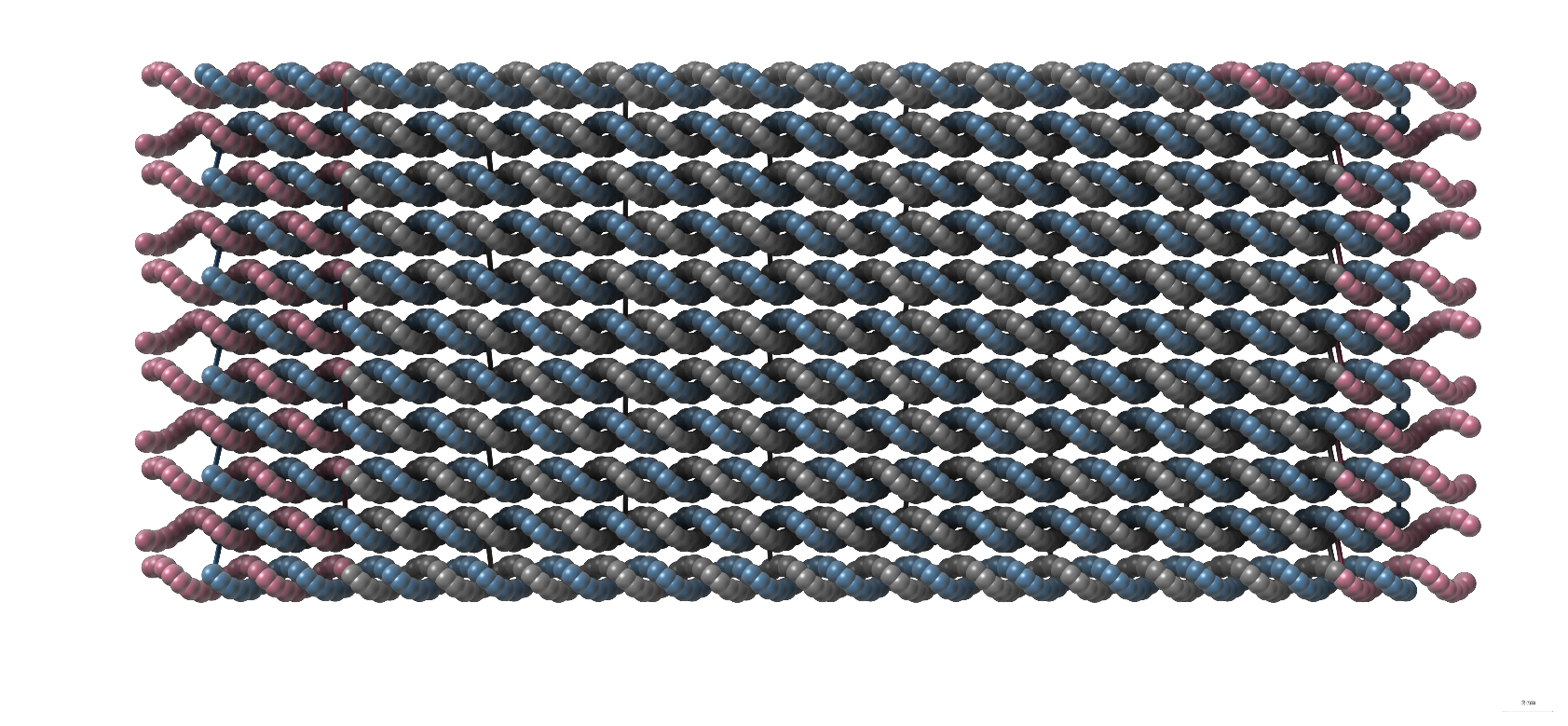


2 nm

15

**Figure S6. 2D and 3D schematic illustration of 16S rRNA:DNA rectangle.** 16S rRNA of *E.Coli* (1542b) is used as scaffold strands (blue). Core and edge DNA staples are colored in gray and fuchsia respectively. 5’ of the scaffold strand is indicated by black arrow.

2D and 3D cuboctahedrons

2D and 3D cuboctahedrons were designed manually based on Zhang et al.,[^11^](https://paperpile.com/c/FGinqH/E2Vfk) work describing complex wireframe DNA origami nanostructures , adapting the staples’ helical periodicity to A-helix geometry (11-fold).


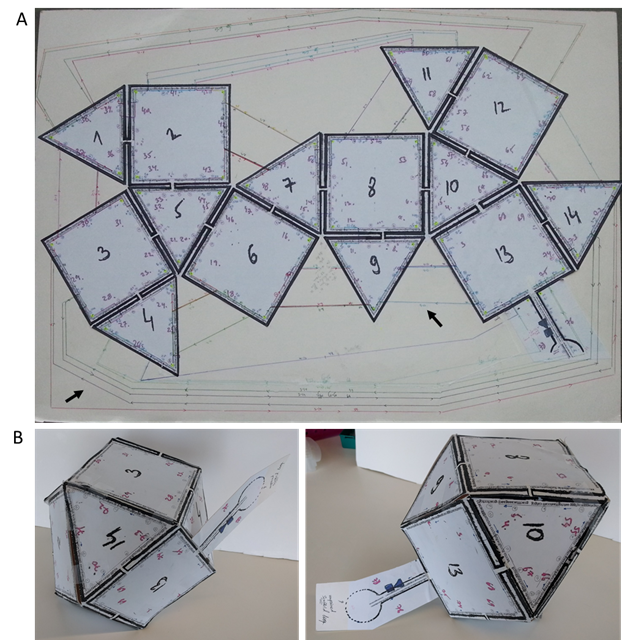


**Figure S7. Schematic representation of 2D and 3D cuboctahedrons. A,** Schematic representation of 2D conformation and the cross-face staples required for 3D conformation (example of such staples indicated by arrows). **B,** Schematic representation of the 3D conformation.

**Supplementary note 5**

**Additional tested folding protocols**

Additional tested folding protocols for folding of 18S rRNA:DNA rectangles

Folding protocol 1:

- 60 ºC, 1 min
- 55 ºC, 5 min
- 50 ºC, 10 min
- 37 ºC, 10 min
- 25 ºC, 10 min

Folding protocol 2:

- 60 ºC, 1 min
- 55 ºC, 5 min
- 50 ºC, 10 min
- 37 ºC, 10 min
- 25 ºC, 10 min
- 37 ºC, up to 10 days to improve yields

AFM images are found in the article **(Fig. 2D, 2E)**.

Additional tested folding protocols for folding of 26S rRNA:DNA rectangles

Folding protocol 1:

- 60 ºC, 1 min
- 55 ºC, 5 min
- 50 ºC, 10 min
- 37 ºC, 10 min
- 25 ºC, 10 min

**
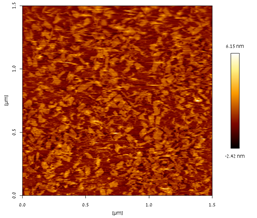

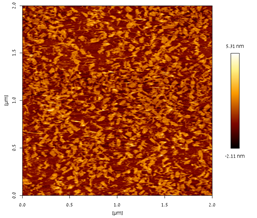
**

**Fig S8.** 26S rRNA:DNA rectangles folded according to folding protocol 1

Folding protocol 2:

- 60 ºC, 1.5 min
- 55 ºC, 7.5 min
- 50 ºC, 15 min
- 37 ºC, 15 min
- 25 ºC, 15 min


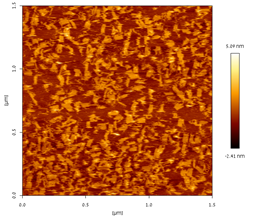

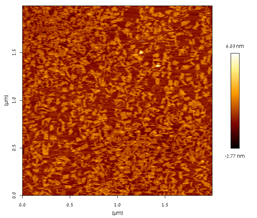


**Fig S9.** 26S rRNA:DNA rectangles folded according to folding protocol 2

Folding protocol 3:

- 60 ºC, 1min
- 59 ºC - 56 ºC, -1 ºC/0.5 min
- 55 ºC, 5 min
- 54 ºC - 51 ºC, -1 ºC/0.5 min
- 50 ºC, 10 min
- 49 ºC - 38 ºC, -1 ºC/0.5 min
- 37 ºC, 10 min
- 36 ºC - 26 ºC, -1 ºC/0.5 min
- 25 ºC, 10 min


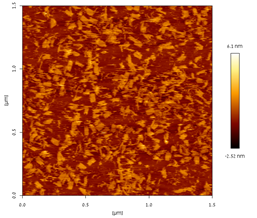

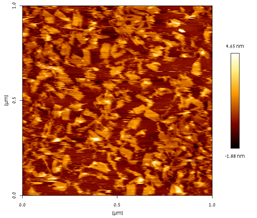


**Fig S10.** 26S rRNA:DNA rectangles folded according to folding protocol 3

Folding protocol 4:

- 60 ºC, 1min
- 59 ºC - 56 ºC, -1 ºC/0.5 min
- 55 ºC - 25 ºC, -1 ºC /1 min

**
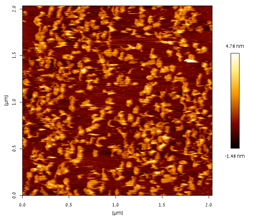

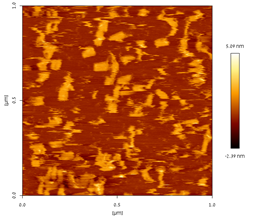
**

**Fig S11.** 26S rRNA:DNA rectangles folded according to folding protocol 4

Additional tested folding protocols mix folding of 18S and 26S rRNA:DNA rectangles

Folding protocol 1:

- 60 ºC, 1 min
- 55 ºC, 5 min
- 50 ºC, 10 min
- 37 ºC, 10 min
- 25 ºC, 10 min
- 37 ºC, 7 days to improve yields


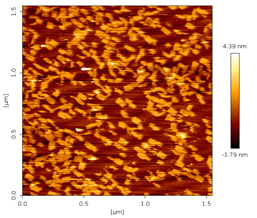

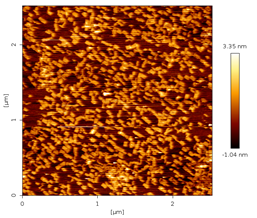


**Fig S12.** 18S and 26S rRNA:DNA rectangles folded according to folding protocol 1

Additional tested folding protocols for folding of 2D and 3D cuboctahedron

Folding protocol 1:

- 60 ºC, 1 min
- 55 ºC, 5 min
- 50 ºC, 5 min
- 45 ºC, 10 min
- 40 ºC - 15 ºC, -1 ºC/10 min
- 37 ºC, up to 4 days to improve yields


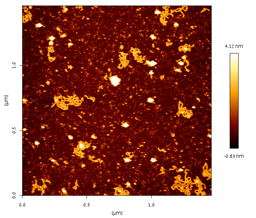

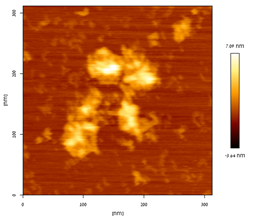


**Fig S13.** 2D and 3D rRNA:DNA cuboctahedron s folded according to folding protocol

**Supplementary note 6**

**Filtration and purification of folded 18S rRNA:DNA rectangles**

Folded 18S rRNA:DNA rectangles were filtered and purified to remove staple excess using Amicon Ultra with two different cut-off membranes: 30 K & 100 K. All samples were centrifuged at 5000 g and 10,000 g forces as described above. Purified and enriched 18S rRNA:DNA rectangles were observed only when using Amicon 100 kDa at both forces (5000 g and 10,000 g). Filtration of samples using Amicon 30 kDa at both forces did not succeed, and staples were still apparent in solution as evident in the agarose gel.


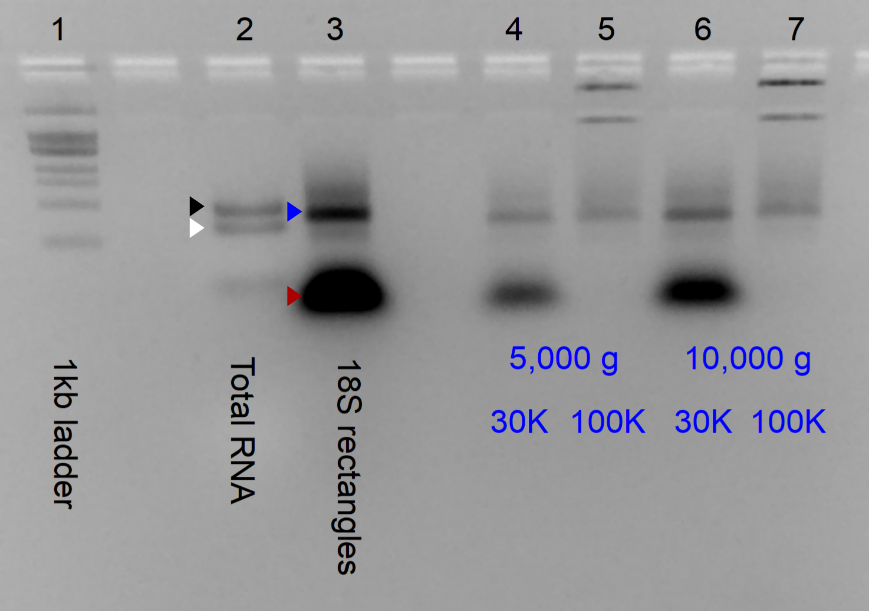


**Figure S14. Filtration and purification of 18S rRNA:DNA rectangles.** Agarose gel electrophoresis demonstrating folded 18S rRNA:DNA rectangles before and after filtration process to remove staple excess using Amicon Ultra 30 K and 100 K at two different centrifugal forces: 5000 g and 10,000 g. The black arrow represents the bands of 26S subunits, the white arrow indicates the bands of 18S subunits, the blue arrow marks the bands of the folded 18S rRNA:DNA rectangles and the red arrow staples’ leftovers.

**Supplementary note 7**

**K_10_-PEG_5K_ coating and uncoating using chondroitin sulfate of 18S rRNA:DNA rectangles**

Folded 18S rRNA:DNA rectangles were purified using Amicon Ultra 100 kDa (cut-off membrane) as described above to remove staple excess. Following, shapes were coated with K_10_-PEG_5K_ to improve structures stability and integrity. Successful coating, appeared as absence of migration in agarose gel electrophoresis (AEG) due to increased Mw of the structures. 18S rectangle retained its regular AEG running pattern after removal of the K_10_-PEG_5K_ shell by addition of chondroitin sulfate at × 1000 excess (described in materials and methods section), indicating that the structure and integrity were not affected by chondroitin sulfate.


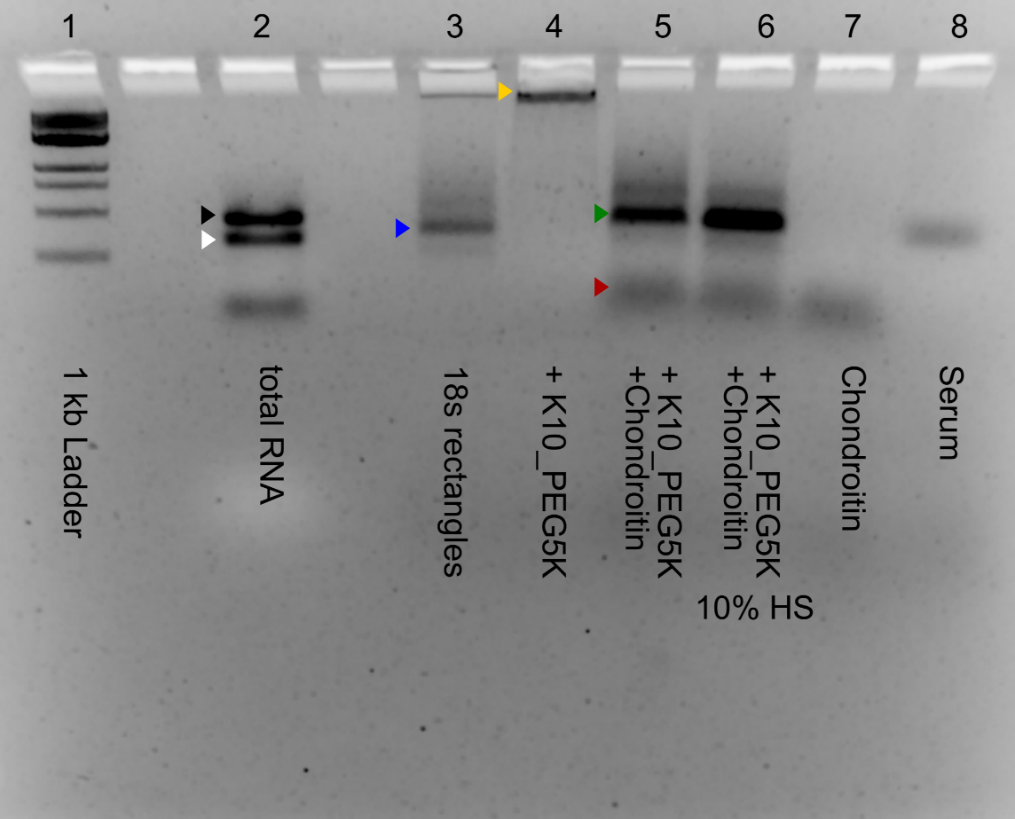


**Figure S15. K_10_-PEG_5K_ Coating and chondroitin sulfate uncoating of 18S rRNA:DNA rectangles. A,** Agarose gel electrophoresis demonstrating folded 18S rRNA:DNA rectangles (lane 3), K_10_-PEG_5K_ coated rectangles (lanes 4) and 18S rRNA:DNA rectangles after removal of the K_10_-PEG_5K_ shell in folding buffer using chondroitin sulfate (lane 5). Lane 6, represented 18S rRNA:DNA rectangles that were maintained in folding buffer supplemented with 10 % human serum. Prior to running in agarose gel, the K_10_-PEG_5K_ coating was removed. Lane 1 represent 1kb ladder, lane 2 contains total RNA extracted from *S.Cerevisiae* and lane 3 contains sample of purified folded 18S rectangles prior the K_10_-PEG_5K_ coating & chondroitin uncoating step. Lanes 7 and 8 contained chondroitin and human serum only respectively. The black arrow represents the bands of 26S subunits, the white arrow indicates the bands of 18S subunits, the blue arrow marks the bands of the folded 18S rRNA:DNA rectangles and the red arrow staples’ leftovers. As for the coating and removal of the K_10_-PEG_5K_ shell - the yellow indicated the K_10_-PEG_5K_ coated 18S rectangles, and the green the 18S rectangles after removal of the coating.

**Supplementary note 8**

**Total RNA extraction from *S. cerevisiae***

A

**
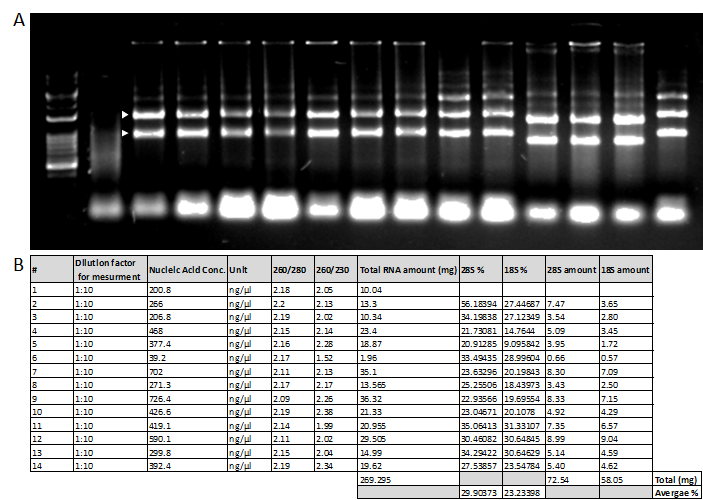
**

B

**Figure S16. Total RNA extraction from *S. cerevisiae*. A,** Gel electrophoresis of extracted samples. Lane 1 contains 1 kb marker, while lanes 2-15 contains the samples. In sample 1 (lane 2) the RNA was degraded. Top arrow indicates the 26S rRNA band, while the bottom indicates the 18S rRNA band. The lowest bands represent all the rest extracted RNA molecules. **B,** 18S and 26S rRNA quantification using Nanodrop (NanoDrop 2000C, Thermo Scientific).

**Supplementary note 9**

**Improved folding yields and shaped integrity by maintaining the samples at 37 ºC upon folding**

18S rRNA:DNA rectangle were folded in 1 × TAE, 12 mM MgCl_2_ buffer in a thermal cycler according to the following folding protocol: 60 ºC/1 min, following by 55 ºC/5 min, and 10 min at 50 ºC, 37 ºC and 25 ºC. Upon folding, the samples maintained at 37 ºC over 9 days as part of a stability assay at human body temperature. Interestingly, the gel indicated that keeping the samples at 37 ºC over a period of 9 days improved the folding yields by ~ 40% , as it was later confirmed by AFM **(Fig. 2E, 2F).**


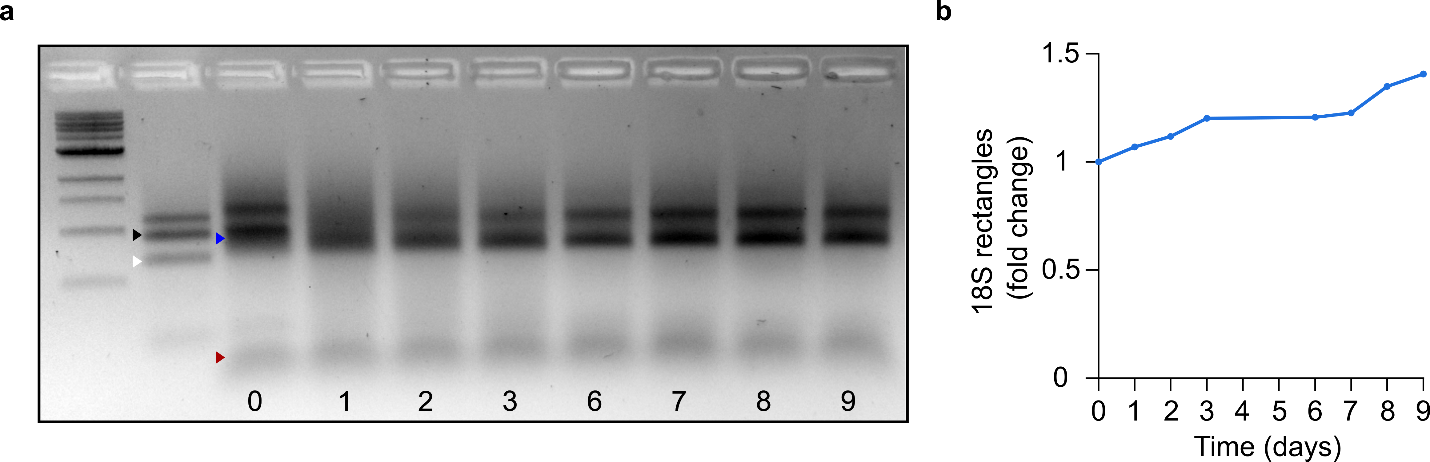


**Figure S17. Improved yields of 18S rRNA:DNA rectangles held at 37 ºC over following folding over 9 days. A, Agarose gel analysis.** Lane 1 and lane 2 contains 1kb marker and total RNA extracted from S. cerevisiae respectively. Lanes 3-10 contains samples of folded 18S rectangles that were kept at 37 ºC over 9 days. Numbers below the lanes indicate the days.  The black arrow represents the bands of 26S subunits, the white arrow indicates the bands of 18S subunits, the blue arrow marks the bands of the folded 18S rRNA:DNA rectangles and the red arrow staples’ leftovers. **B, Graph representing the fold change of the folded** 18S rRNA rectangles while being kept at 37 ºC during a period of 9 days. Total band intensity of each time point was normalized to time point 0, which represents the amount of well-folded 18S rectangles immediately after the folding protocol described above, and indicated by ‘1’. Quantification was performed using Image Lab gel analysis software (Bio-Rad, version 6.0.1).

**Supplementary note 10**

**The effect of MgCl_2_ concentration and edge staples on the folding of 18S rectangle**

18S rRNA:DNA were folded as described in the material and method section once with edge staples, and once without, at MgCl_2_ concentrations ranging from 10 - 20 mM, at +2 mM steps. There is no significant difference on the folding when increasing MgCl_2_, however adding 12 edge staples slightly improves the folding.


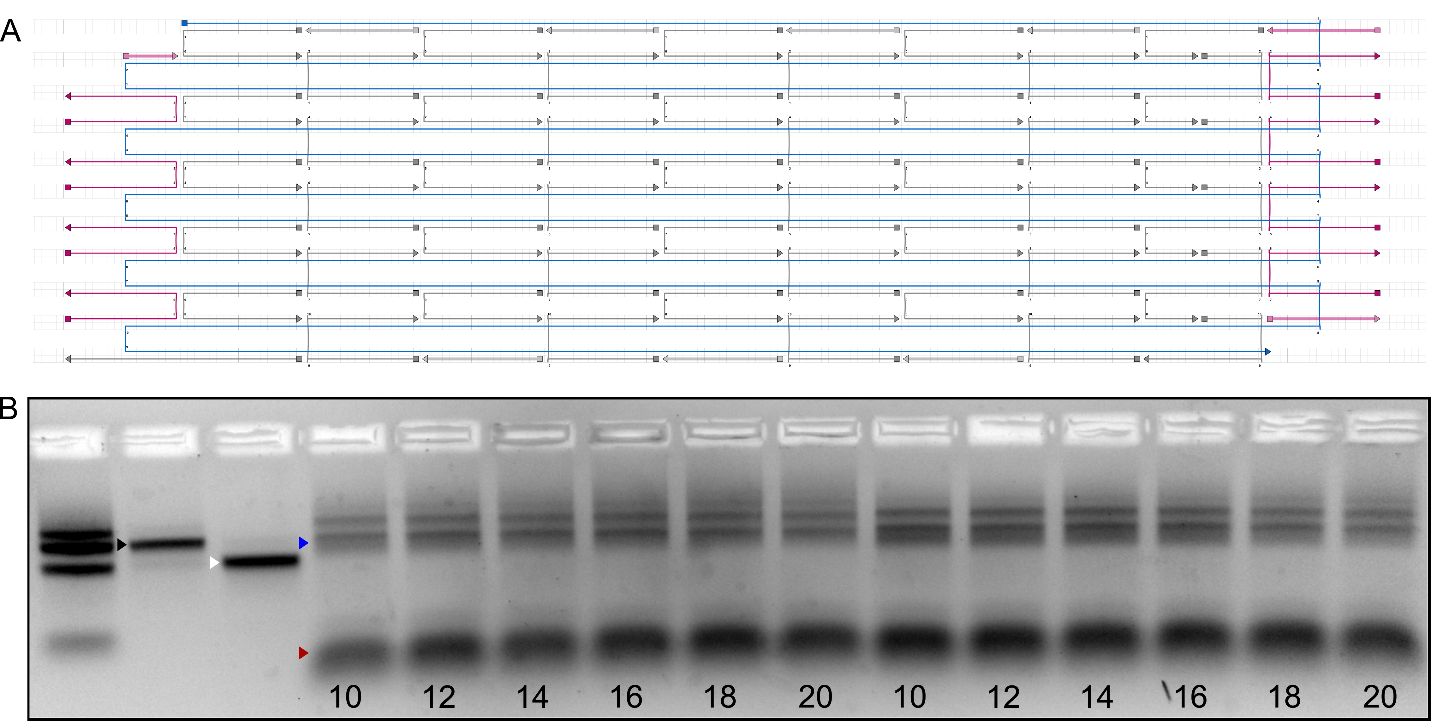


**Figure S18. Effect of edge staples and MgCl_2_ concentration on the folding of 18S rectangles. A,** Schematic representation of 18S rRNA:DNA rectangle. 18S rRNA used as a scaffold strand is colored in blue, while core and edge staples are in gray and fuchsia respectively. **B,** Gel electrophoresis of rectangles folded without (lanes 4 – 9) and with edge staples (lanes 10 – 15) at different MgCl_2_ concentrations ranging from 10 - 20 mM (indicated below the lames), at +2 mM steps. The white arrow indicates the band of the folded 18S rectangles. Lane 1-3, represents 1 kb, 26S rRNA and 18S rRNA respectively. The black arrow represents the bands of 26S subunits, the white arrow indicates the bands of 18S subunits, the blue arrow marks the bands of the folded 18S rRNA:DNA rectangles and the red arrow staples’ leftovers.

**Supplementary note 11**

**Critical folding** **temperature prediction of 18S and 26S rectangles assembly**

We configure the critical temperature of folding as the melting temperature required for complete folding of a shape at constant temperature. Based on our model that tracks the folding kinetics of a shape, the critical temperature is where around ~50 % of the shape is folded according to the simulation [^39^](https://paperpile.com/c/FGinqH/uFTIy). To simulate the folding kinetics of 18S and 26S rectangles, we adapted our model to fit RNA:DNA melting table[^40^](https://paperpile.com/c/FGinqH/PZNpW), rather than DNA:DNA, by changing the T_m_ calculation using Bio.SeqUtils. MeltingTemp (python), with the following parameters compatible with our folding conditions (DNA/RNA concentration were given in nM, while were given in mM):

*seq_Tm = mt.Tm_NN(seq, nn_table = mt.R_DNA_NN1, dnac1 = 100, dnac2 = 10, Mg = 12, Na = 1, K = 0, Tris = 40, saltcorr = 0)*

According to the simulation, the predicted critical temperatures of 18S and 26S are around 53 ºC and 56 ºC respectively. Since we saw that total RNA was susceptible to high temperatures **(Fig. 2A)**, and since we wanted to fold both shapes simultaneously, we chose to fold both shapes at 50 ºC.


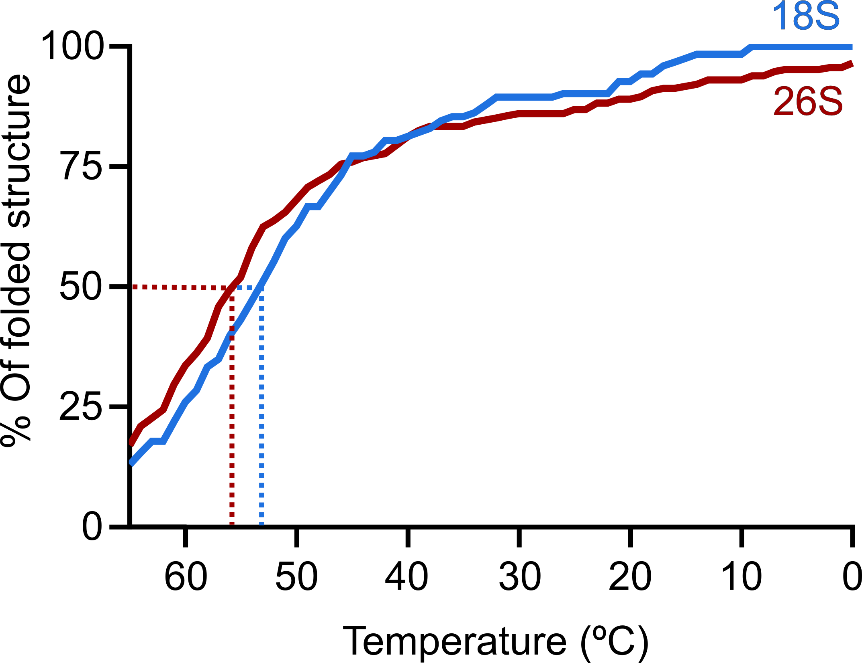


**Fig S19. Critical temperature of folding prediction for 18S and 26S rRNA:DNA rectangles.** 18S is represented in blue, while 26S in red.

**Supplementary Note 12**

**Loading of 18S rRNA:DNA rectangles with Streptavidin**

18S rRNA:DNA rectangle was slightly redesigned, such that 4 core staples (colored in yellow) were replaced by staples comprising biotin at 5’ without changing the nucleotide sequence **(Fig. S20A)**. Shapes were folded, and analyzed by agarose gel electrophoresis **(Fig. S20B)** and atomic force microscopy **(Fig. S20C)** as described in the **material and methods section**.


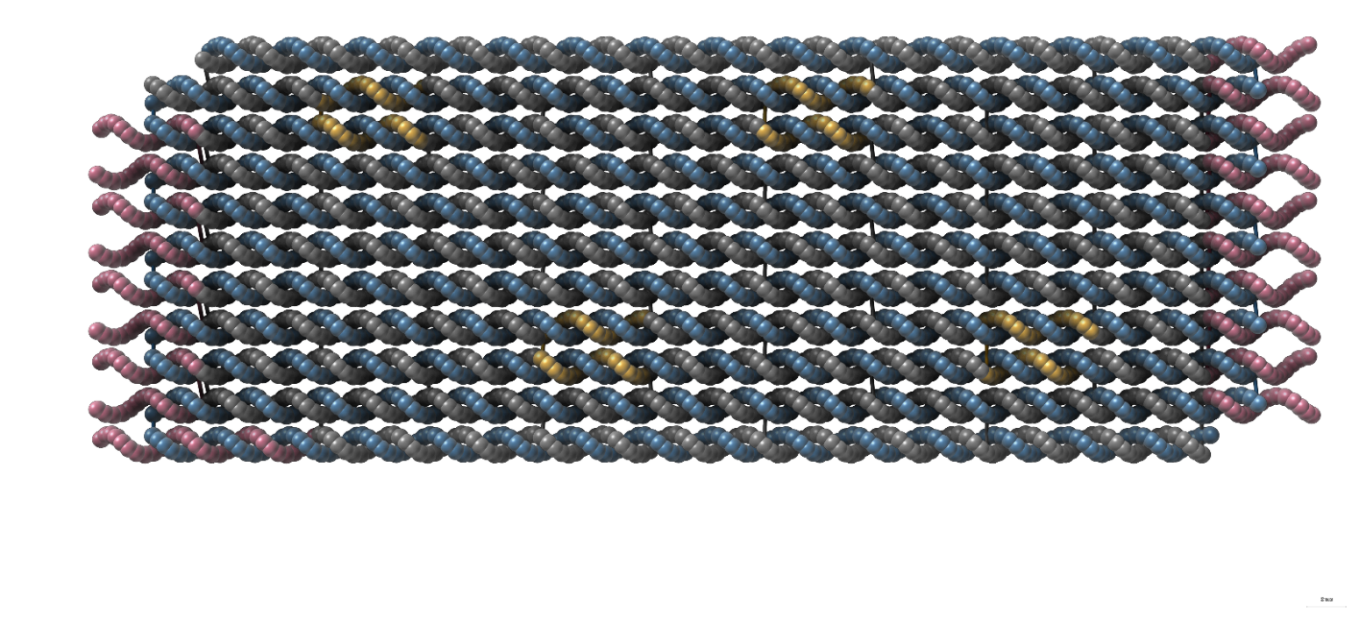
A

**
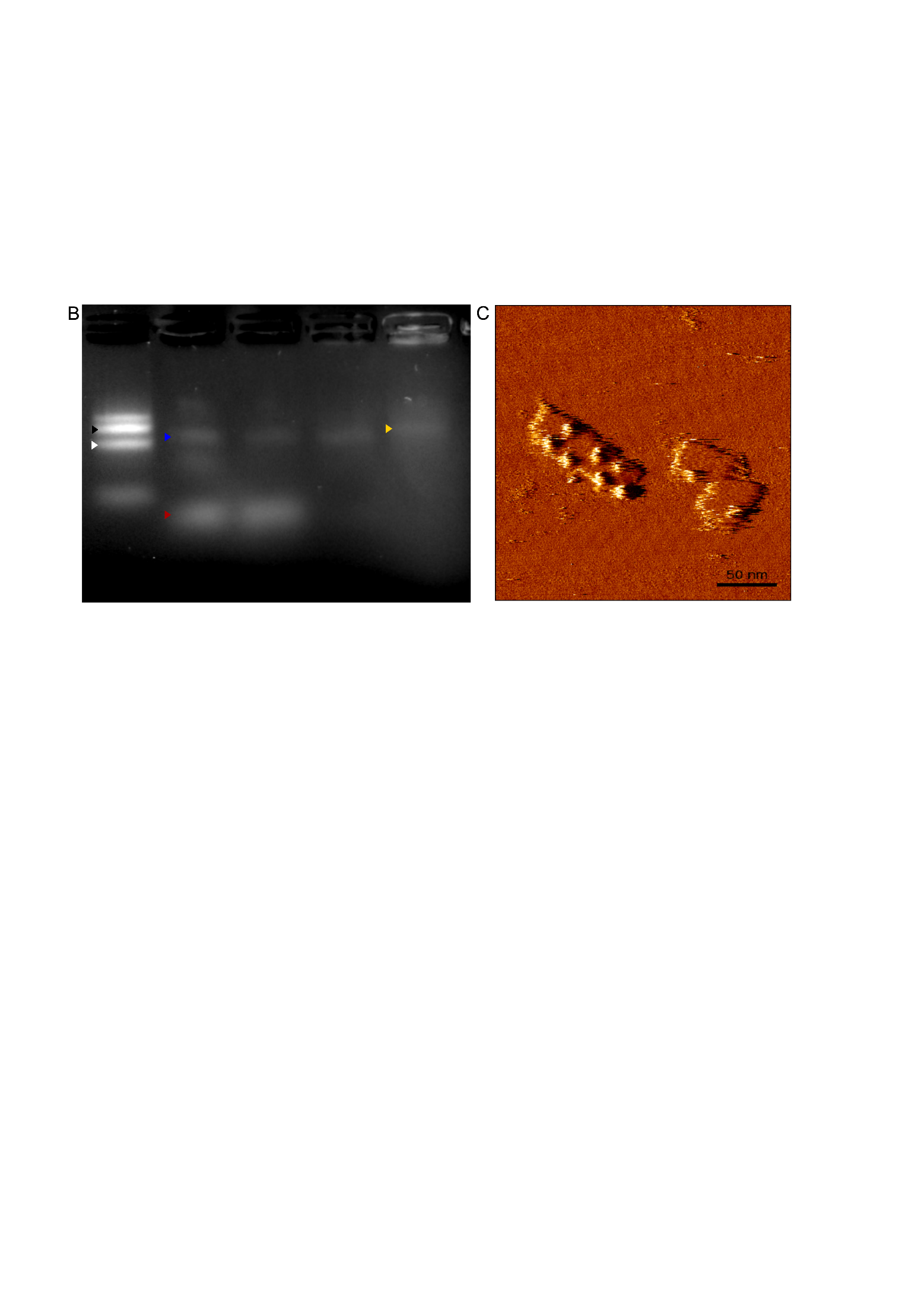
**

**Figure S20. Loading of 18S rRNA:DNA rectangles with Streptavidin. A,** Schematic illustration of 18S rectangles comprising four binding sites for streptavidin molecules- 4 yellow staples with biotin tagged to 5’. **B,** Agarose gel electrophoresis demonstrating the folded structures (lane 2 & 3), which were purified using Amicon Ultra 100K (cut-off membrane) from staple leftovers (lane 5) and coated with streptavidin (lane 6). Lane 1 represents 1kb ladder. **C,** AFM image of Streptavidin molecules bound to 18S rectangles at specific designed four binding sites (4 staples comprising biotin at 5’)**.**

**Supplementary Note 13**

**Shelf life of 18S rRNA:DNA rectangles at 37 ºC and 50 ºC**

**
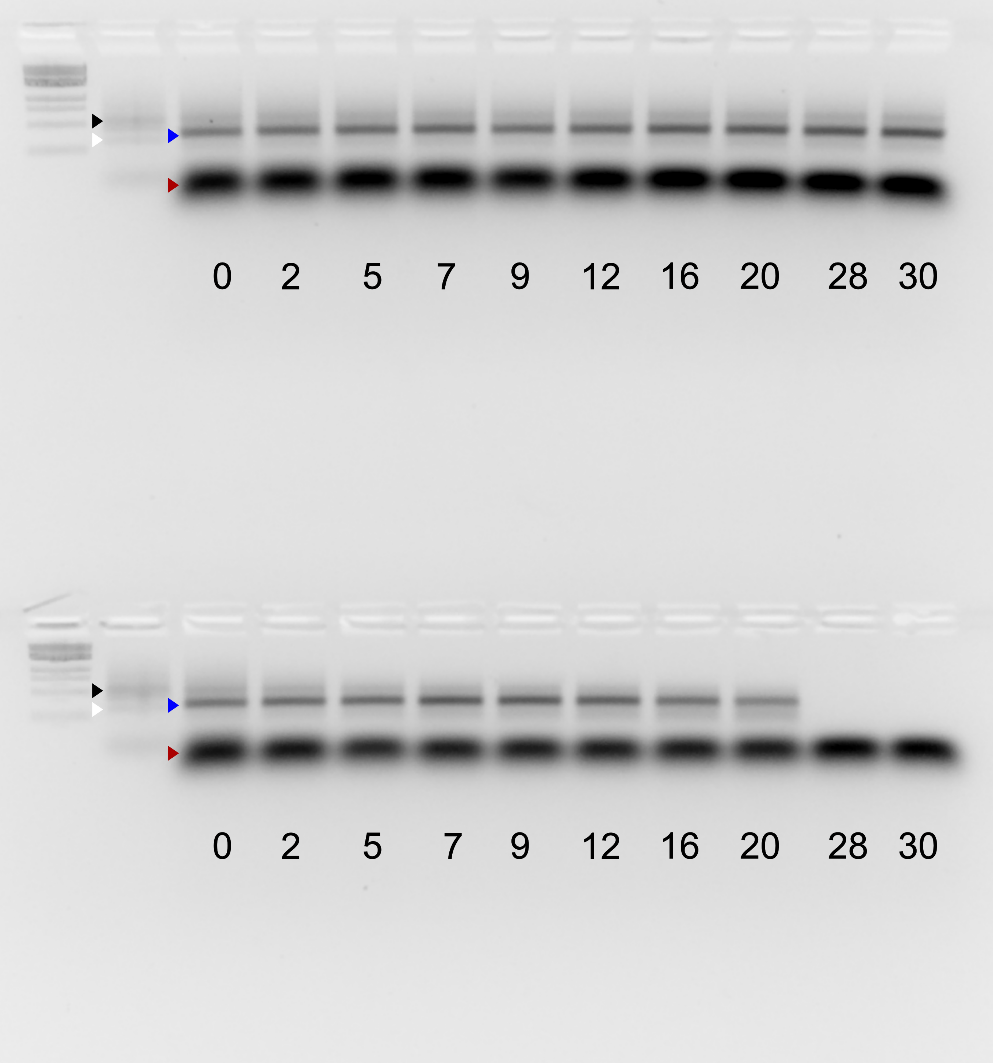
**

**Figure S21. Shelf life of 18S rRNA:DNA rectangles at 37 ºC and 50 ºC over a month.** Representative agarose gel of 18S rRNA:DNA rectangles kept at 37 ºC (top gel) and 50 ºC (bottom gel) for a month to assess life shelf. Bands’ intensities of the folded structures at each time point were translated to % of the stable structures, as time point = 0 d represented 100 %. The black arrow represents the bands of 26S subunits, the white arrow indicates the bands of 18S subunits, the blue arrow marks the bands of the folded 18S rRNA:DNA rectangles respectively, and the red arrow indicates the bands of the staple leftovers as well as the degraded rectangles. Collected time points indicated below in days. Lane 1 contains 1 kb marker, while lane 2 contains total RNA extracted from S. *cerevisiae*.

**Supplementary note 14**

**DNA-DNA rectangle resistance to DNaseI**

Rothmunds’ well known DNA:DNA rectangles (p7249) were folded and purified. Subsequentially, DNA rectangles were diluted in a folding buffer (1 × TAE, 12 mM MgCl_2_) containing 2 units of DNase I such that the final Mg^2+^ concentration was adjusted to 12 mM. Subsequently, the samples were incubated at 37 ºC over 1 hour in a thermal cycler, and run on 1 % agarose gel for analysis.

**
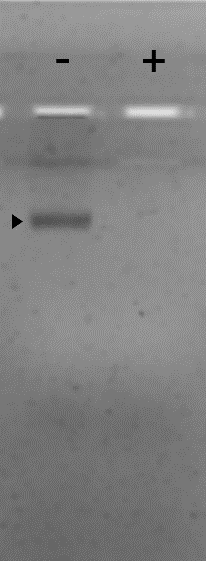
**

**Figure S22. DNA-DNA rectangle resistance to DNaseI.** The band of well folded DNA rectangles are indicated by black arrow. In the control sample, which did not contained DNase I (lane 1, indicated by ‘-‘), the rectangles remain stable, while DNA rectangles in the presence of 2-units of DNase I were totally degraded after 1 hour (lane 2, indicated by ‘+’).

**Supplementary note 15**

**AFM images of K_10_-PEG_5K_ coated 18S rRNA-DNA rectangles**

18S rRNA:DNA (18S rRNA extracted from *S. cerevisiae*) rectangles were folded, purified using Amicon 100K and then coated using polylysine-polyethelyne glycol K_10_-PEG_5K_ as described in the **materials and methods section**. Structures’ shape and integrity after coating was verified by agarose gel and atomic force microscopy (AFM).


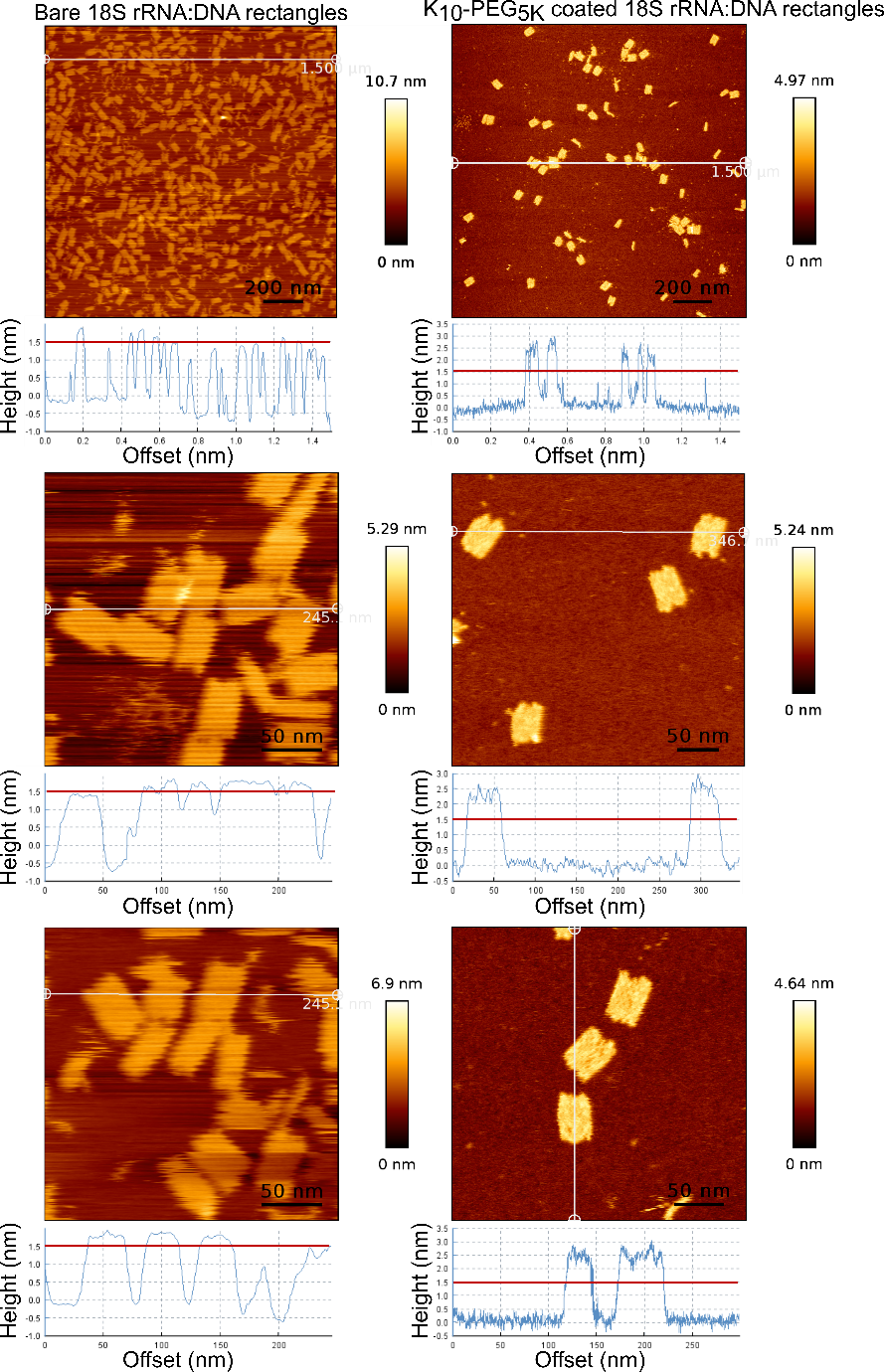


**Figure S23. AFM images of K_10_-PEG_5K_ coated 18S rRNA:DNA rectangles.** Left column represents bare 18S rRNA:DNA rectangles, while right column K_10_-PEG_5K_ rectangles. Hight cross-section profile are added below each picture. White lines represent the cross sections, and the red lines indicate of 1.5 nm height.

**Supplementary note 16**

**Tolerance of 18S rRNA:DNA rectangles to treatment with DNase I**

Upon folding of 18S rRNA:DNA rectangles, samples were purified and enriched using filtration to remove staple leftovers. Following half of the samples were coated using K_10_-PEG_5K_, while the other remained “Bare”. Next, 2-units of DNase I were added to all the samples, and MgCl^2+^ concentrations were adjusted to 12 mM. The resistance of coated vs. bare 18S rectangles against DNase I was tested over 24 hours while the samples maintained at 37 ºC.

**
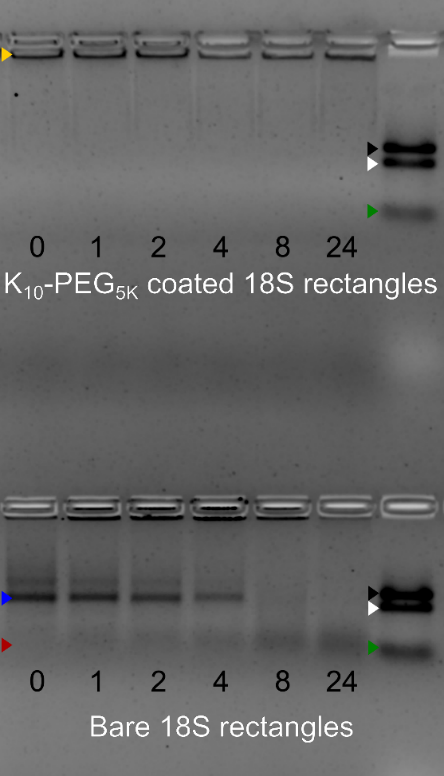
**

**Figure S24. Tolerance of 18S bare and K_10_-PEG_5K_ coated rRNA:DNA rectangles to treatment with DNase I.** Representative agarose gel of K_10_-PEG_5K_ coated (top) and bare (bottom) 18S rRNA:DNA rectangles in the presence of 2-units of DNase I at 37 ºC over 24 hours. K_10_-PEG_5K_ shell was not removed from the coated sample before loading the samples on agarose gel to prevent degradation. Bands’ intensities of the folded structures at each time point were translated to % of the stability, as time point = 0 d represented 100%. The black arrow represents the bands of 26S subunits, the white arrow indicates the bands of 18S subunits, the yellow and blue arrows mark the bands of the K_10_-PEG_5K_ coated or bare folded 18S rRNA:DNA rectangles respectively. The red arrow indicates the bands of the degraded rectangles, while the green band indicates the “total” RNA molecules extracted from cells (lanes 7). Collected time points indicated below in hours. Lane 7 contained total RNA extracted from *S. cerevisiae*.

**Supplementary note 17**

**Tolerance of 18S rRNA:DNA rectangles to treatment with different amounts of RNase H**

Upon folding of 18S rRNA:DNA rectangles, samples were purified and enriched using filtration to remove staple leftovers. Following half of the samples were coated using K_10_-PEG_5K_, while the other remained “Bare”. Next, different amounts of RNase H were added to all the samples, and MgCl^2+^ concentrations were adjusted to 12 mM. The resistance of coated vs. bare 18S rectangles against RNase H was tested over 20 minutes while the samples maintained at 37 ºC.


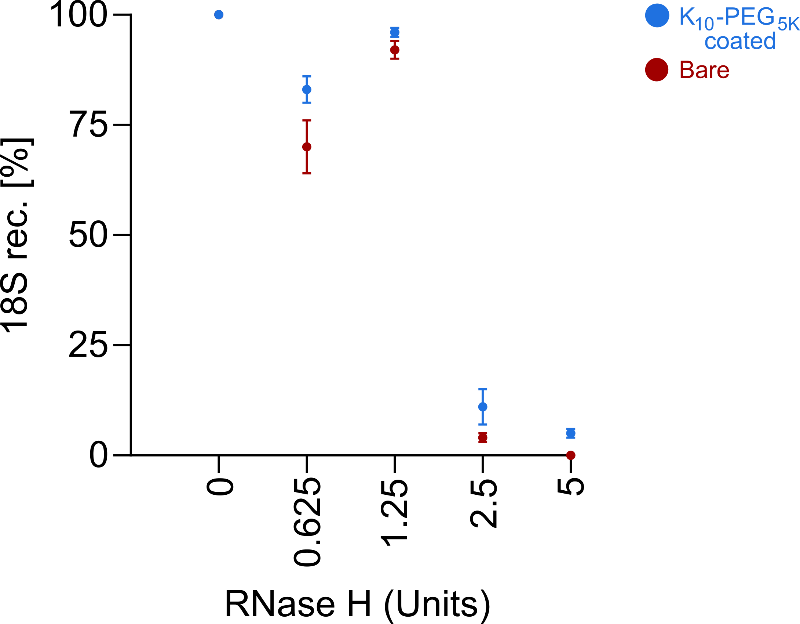


**Figure S25. Tolerance of 18S bare and K_10_-PEG_5K_ coated rRNA:DNA rectangles to treatment with different amounts of RNase H.** Stability of K_10_-PEG_5K_ coated (blue) and bare (red) 18S rRNA:DNA rectangles in the presence of x-units (where x is = 0, 0.625, 1.25, 2.5 and 5 units) of RNase H after 20 minutes at 37 ºC. At time point 0, both the bare and the K_10_-PEG_5K_ coated rectangles are at 100%.

**Supplementary note 18**

**Tolerance of 18S rRNA:DNA rectangles to treatment with 1.25 units of RNase H over 4 hours**

Upon folding of 18S rRNA:DNA rectangles, samples were purified and enriched using filtration to remove staple leftovers. Following half of the samples were coated using K_10_-PEG_5K_, while the other remained “Bare”. Next, 1.25-units of RNase H were added to all the samples, and MgCl^2+^ concentrations were adjusted to 12 mM. The resistance of coated vs. bare 18S rectangles against RNase H was tested over a period of 4 hours while the samples maintained at 37 ºC.


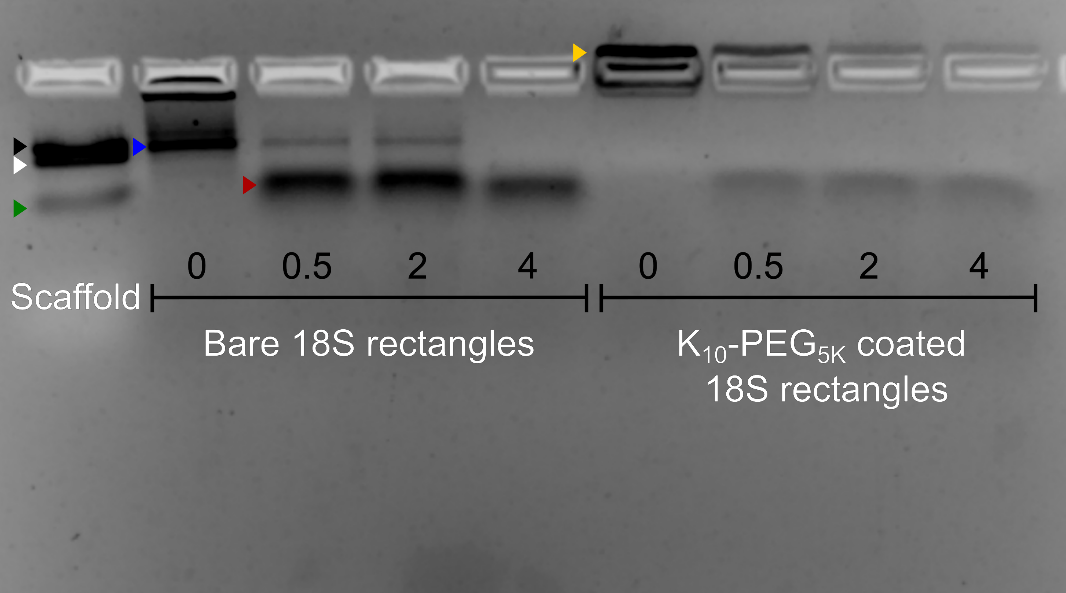


**Figure S26. Tolerance of 18S bare and K_10_-PEG_5K_ coated rRNA:DNA rectangles to treatment with 1.25-units of RNase H.** Representative agarose gel of K_10_-PEG_5K_ coated and bare 18S rRNA:DNA rectangles in the presence of 1.25-units of RNase H at 37 ºC over a period of 4 hours. K_10_-PEG_5K_ shell was not removed from the coated sample before loading the samples on agarose gel to prevent degradation. Bands’ intensities of the folded structures at each time point were translated to % of the stability, as time point = 0 d represented 100%. The black arrow represents the bands of 26S subunits, the white arrow indicates the bands of 18S subunits, the yellow and blue arrows mark the bands of the K_10_-PEG_5K_ coated or bare well-folded 18S rRNA:DNA rectangles respectively. The red arrow indicates the bands of the degraded rectangles, while the green band indicates the “total” RNA molecules extracted from cells (lanes 7). Collected time points indicated below in hours . Lane 1 contained total RNA extracted from *S. cerevisiae*.

**Supplementary note 19**

**18S rRNA:DNA rectangles stability in 10 % human serum**


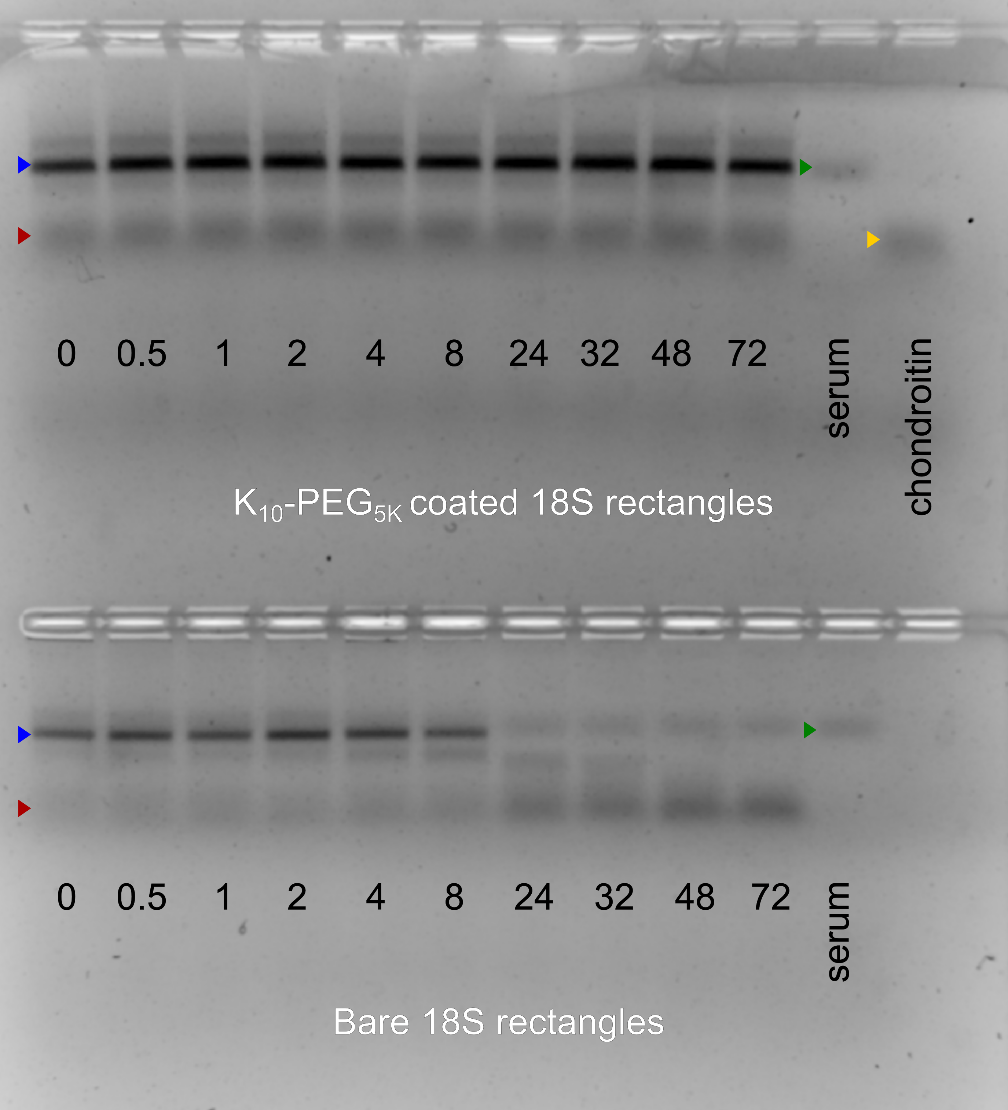


**Figure S27. 18S rRNA:DNA rectangles stability in 10 % human serum.** Representative agarose gel of 18S rRNA:DNA rectangles kept at 37 ºC in folding buffer (1 × TAE, 12 mM MgCl_2_) supplemented with 10 % human serum. Top gel represents 18S rectangles coated with K_10_-PEG_5K_ to improve stability, while bottom gel represents bare rectangles. Bands’ intensities of the folded structures at each time point were translated to % of the stability, as time point = 0 min represented 100%. The blue arrows mark the bands of the K_10_-PEG_5K_ coated (and later removed) or bare folded 18S rRNA:DNA rectangles. The red arrow indicates the bands of the degraded rectangles, the green arrow indicates the serum while the yellow represents the chondroitin sulfate. Collected time points indicated below in hours.

**Supplementary note 20**

**Growth inhibition of *Staphylococcus aureus* and *Escherichia coli***

Through our extensive research into rRNA and rRNA:DNA origamis, we formulated a hypothesis suggesting that employing origami folding techniques to lock biologically-active rRNA within bacterial cells could potentially induce growth inhibition, or even prevent it altogether. To investigate this hypothesis, we conducted experiments to assess the impact on the growth of *Staphylococcus aureus (S. aureus)* and *Escherichia coli (E. coli)*. We started with targeting the smallest rRNA subunit, namely the 5S rRNA, in *S. aureus*. For this purpose, we designed two 45-base RNA staples that would form an immobile junction and induce the folding of the 5S rRNA (comprising 115b) into a small rectangle **(Fig. S28A)**. Notably, Growing *S. aureus* in the presence of these staples (passive uptake of staples) resulted in growth inhibition **(Fig. S28B)**. Next, we wanted to assess the effect of folding a larger rRNA subunit on the growth of *E.coli*. In contrast to the passive uptake of the staples by *S. aureus*, our approach here involved encoding the staples on a plasmid, since folding a larger shape required a greater quantity of longer staples. However, we encountered synthesis and production limitations related to the number of staples with repetitive regions that could effectively be encoded on one plasmid.

In light of these constraints, we redesigned our 16S rRNA-DNA rectangle and extended the length of the staples. Four of these staples (colored in red in **Fig. S28C**) were encoded on the plasmid (P plasmid), each governed by T7 promoter and lac operon transcription regulation. Consequently, the transcribed staples were RNA staples, and upon hybridization with the 16S rRNA, they formed RNA:RNA duplexes that retain a 11-fold helical geometry, resembling the rRNA:RNA duplexes.

The P plasmid, carrying the encoded staples, was transformed into *E.coli* via a heat shock, and *E. coli* were grown overnight at 37 ºC in U-bottom 96-well plates in a plate reader or on LB-Agar separate plates in an incubator, with both setups involving the presence of 50 µg/mL Kanamycin to maintain plasmid selection. The addition of IPTG, acting as an inducer, at a low concentration (0.1 mM) resulted in the inhibition of E. coli growth, while at higher IPTG concentrations (>= 0.5mM), the transcribed staples effectively hindered E. coli growth over a period of 1 day **(Fig. S28D, S28E).**

The ctrl plasmid, which does not encode to any staple, did not show inhibiting effect on E. coli growth **(Fig. S29)**.

**
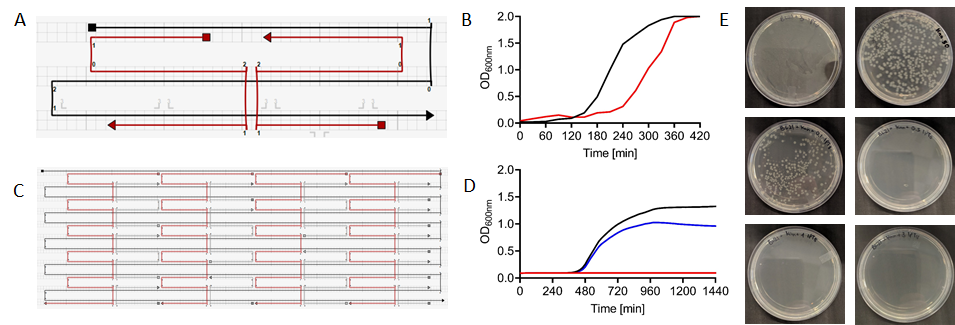
**

**Figure S28. Bacteria growth inhibition. A,** Schematic illustration of the staples designed to 5S rRNA and folding it into a rRNA-RNA rectangle**. B,** *S.* *aureus* growth curve over 8 hours in the presence of the staples (red). Black curve represents S. aureus that were not treated. **C,** Schematic illustration of the staples designed to 16S rRNA of E.coli and folding it into a rRNA-RNA rectangle**. D,** E. coli growth curve over 24 hours in the presence of 50 mM Kanamycin and addition of different IPTG concentrations: 0 mM (black), 0.1 mM (blue) and 0.5 mM (red). 1mM, 2mM and 3mM showed the same results as the 0.5 mM IPTG concentration. **E,** E. coli growth in agar plates containing different IPTG concentration over 24 hours. All plates, besides the top left, contained Kanamycin and E. coli transformed with P plasmid. Top: left - Native E. coli that were grown in plates containing 3 mM IPTG, right - 0 mM IPTG. Middle row: left - 0.1 mM IPTG, right - 0.5 mM IPTG, bottom: left - 1 mM IPTG, right - 3 mM IPTG.

Effect of the ctrl plasmid and IPTG concentration on E. coli growth

As a control, we assessed the impact of the ctrl plasmid at various IPTG concentrations on the growth of *E. coli*. The ctrl plasmid was designed to share the same backbone as the P plasmid, although it did not encode any staple or oligonucleotide, despite containing an insert.  *E. coli* cells transformed with the ctrl plasmid were grown in a 96-well plate within a plate reader at 37 ºC overnight. The control plasmid, even upon addition of IPTG at various concentrations, did not exhibit any inhibition or prevention of *E. coli* growth (**Fig. S29**) as opposed to the observed effects of the P plasmid (**Fig. S28D, S28E**). Interestingly, the presence of IPTG results in an increase in *E. coli* growth **(S29A)**.

**
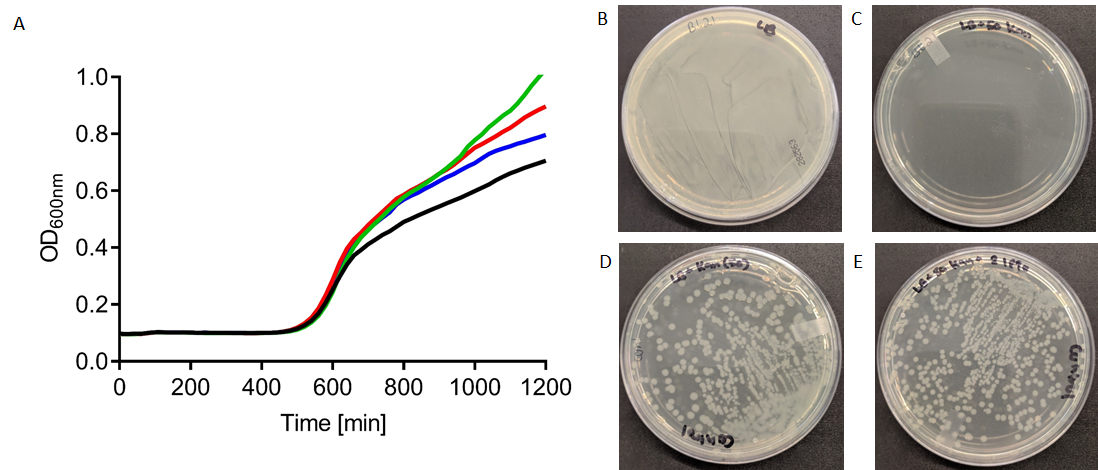
**

**Fig S29. Effect of control plasmid on E. coli growth. A,** E. coli transformed with ctrl plasmid, and grown in 50 µg/mL Kanamycin and different IPTG concentrations: 0, 0.1 mM, 0.5 mM and 2 mM represented by black, blue, red and green curves respectively. **B,** E. coli grown on LB. **C,** E. coli grown on LB with 50 µg/mL Kanamycin. **D,** Transformed E. coli with ctrl plasmid grown on LB with 50 µg/mL Kanamycin. **E,** Transformed E. coli with ctrl plasmid grown on LB with 50 µg/mL Kanamycin and 2 mM IPTG.

**Plasmid design.** The plasmids, P and Ctrl, have PJ281 backbones and were ordered from ATUM. The Ctrl plasmid does not encode to any staple although it contains an insert with sp1 sequence. P plasmid contains an insert that encodes to four staples, which were designed to fold 16S rRNA of E. coli. To enable transcription of the staples inside the cells, T7 promoter was added at 5’ of each staple (TAATACGACTCACTATAGGGG), and transcription termination sequence at 3’ (CTAGCATAACCCCTTGGGGCCTCTAAACGGGTCTTGAGGGGTTTTTTG). Between the staples we added 8×T followed by one of the spacers (sp1, sp2 and sp3). All sequences and the full inserts are listed in **Supplementary note 21**.

*E.coli* growth in the presence of different plasmids in 96-well plates in plate reader. DH5α were transformed with P or Ctrl plasmid for preparation of stab culture (RBC HIT Competent cell) and plasmids’ purification (Qiagen, QIAprep Spin Miniprep Kit) following the manufacturer's instructions. BL21 (DE3) (New England Biolabs, NEB-C2527H) were transformed with P plasmid following the manufacturer's protocol. Briefly, BL21 and the plasmids (P and Ctrl) were thawed on ice for 10 minutes. 1-1.3 µl of plasmid (294.6 ng) was added to 50 µl BL21, following gentle flicks of the tubes to mix the cells with the plasmid. Subsequently the mixture was placed on ice for 30 min, following a heat shock at exactly 40 ºC for 10 seconds and then holding the samples for 5 minutes on ice. Next, 950 µl of SOC (at room temperature) were added to the cells. The samples were diluted by 64-fold with LB in 15 mL tubes, and 200 µl of each sample were seeded in 96 well plate (Costar 96 U transparent) and grown in plate reader (Infinite M Plex, TECAN) at 37 ºC while shaking. Upon 1 hour, Kanamycin was added to the samples to final concentration of 50 µg/ml, then after additional 1 hour, IPTG (SIGMA #367931) was added at different concentrations. BL21 were grown overnight at 37 ºC while shaking and OD was measured every 20 minutes.

**LB-Agar plates preparation for *E.coli* experiments.** LB-Agar plates with 50 µg/mL kanamycin and different concentrations IPTG concentration were prepared as follows: LB-Agar was melted in a microwave (in pulses at low power) and transferred into a heated 60 ºC water bath. Next, it was transferred to tubes (20 mL/tube), and Kanamycin (50 µg/mL) and IPTG (0.1, 0.5, 1 and 3 mM) were added. The mixtures were mixed gently and poured on plates to solidify overnight.

***E. coli* growth in LB-Agar plates.** LB-Agar plates with 50µg/ml kanamycin and different IPTG concentration were prepared as follows: LB-Agar was melted in a microwave (in pulses at low power) and transferred into a heated 60 ºC water bath. Next, it was transferred to tubes (20mL/tube), and Kanamycin (50 µg/mL) and IPTG (0.1, 0.5, 1 and 3 mM) were added. The mixtures were mixed gently and poured into plates to solidify overnight. On the following day, BL21(DE3) was transformed as described above, and grown for 1 hour at 250 rpm, 37 ºC in eppendorf, to obtain antibiotics resistance. Meanwhile, plates were heated to 37 ºC. After 1 hour, 100 µl of bacteria was seeded on each plate.

***Staphylococcus aureus* system.** Staph. Aureus was ordered from ATCC (ATCC-BAA-1717)**.** Two 45 base RNA staples were designed to fold 5S rRNA (accession number NC_007795.1) of Staph. aureus into a small rectangle. The oligos were ordered from IDT, and comprise /3CholTEG/ modification to enhance their permeability into the bacteria.

S1 - UAUAGUCACCAGAAUCGACGCUAAGGAGCUCUAGCGGAACGUAAG/3CholTEG/

S2 - GGCAACGUUCUACUUAACUUCUGUGUUCGGAACAGGUGUGACCUC/3CholTEG/

The staples were reconstituted to 100 µM with ultrapure, DNase/RNase free water (Biological Industries, 01-869-1A) and stored at -80 ºC.

S. aureus were grown overnight in 3 mL LB in growing tubes in a shaker incubator at 37 ºC and 250 RPM. On the following day, the bacteria were 1000 times diluted into a final volume of 2 mL (2 µl bacteria were added into 1998 µl LB), and 25 µl of each staple were added (to final concentration of 1.20 µM). Subsequently, the bacteria were incubated at 37 ºC and 250 RPM, and the OD was measured every 30 minutes over 8 hours using spectrophotometers (biochrom, Ultrospec 10). The graphs were generated with GraphPad Prism (version 8.3.0).

**Supplementary note 21**

**Plasmid’s design and list of sequences for inhibiting *E. coli* growth**

At first, we wanted to encode all 53 staples on the plasmid, but since it was complicated to synthesize an insert with many repetitive sequences (T7 promoter, terminator and linkers). Therefore, we adjusted our original 16S rRNA: DNA rectangle design for E. coli, and elongated the existing core staples to ~200 bases by adding additional crossovers. Since rRNA:RNA duplex has an A-helical geometry,11-fold as found in RNA:DNA hybrids, no changes were needed in staples cross-over positions.

Next, we designed an insert comprising four of the core staples. As mentioned in the article, each insert contains its own T7 promoter and terminator. Between the staples there are a unique spacer sequence. The insert was cloned into ATUM pJ281 plasmid under lac operon regulation. For Ctrl we used the same plasmid with an insert containing SP1 sequence.

All the relevant sequences are listed below:

| T7 Promoter | TAATACGACTCACTATAGGGG |
| --- | --- |
| Operator | AATTGTGAGCGGATAACAATT |
| T7 TER | CTAGCATAACCCCTTGGGGCCTCTAAACGGGTCTTGAGGGGTTTTTTG |
| sp1 | ttttttttACCTTCCTTTTCATTTCCCTTCCTTTCCCCCTTTTTTTCCTCTCCTTTCTCCCTTTATTTCTCCTTACCCTTCCTACTTCTCTCTTTCCCTCCCCTCCTCTACTACCCCATCTTTACTCCCCTTTCCTATCCCTCCAACTCACACTAAAC |
| sp2 | ttttttttTCTTCACTTTAACTCACACTACCCCACATCCCCCTCCTACTCAAAAACTCCCCTCCCACAACCCAAACCCACCACACCAACAACAACCAAAACTCACCCACCCCCCCTCACTAACCCAAAAAAAAAACACCACCAACCAACCATAACTAC |
| sp3 | ttttttttTCAATTACCTCTCTCTCATCTCACCTCCTACACCACACCCTCCCAACTAACCACACACCAACACACTCAAACTTCTCTCTATCCCTCCCAATCACCCTACTCCGCGCCTTCCCCTTCCCTTCAGTTTCCCCTTTTTCTCTCTTTCTTCAC |
| st1 | CCGCCAGCGTTCAATCTGAGCCATGATCAAACTAGGTGAGCCGTTACCCCTCATCCTCTCAGACCAGTAACGCTTGCACCCTCCGCCTGCGTGCGCTTTACCCTCCAAGTCGACATCGGTTAGCTCCGGAAGCCAGGTTGCGCTCGTTGCGGTTCCCGGCCGGACCGCTACAAGGCCCGGGAACGTCAACCCACTCCCATGGTGTGACGGG |
| st2 | CCGCCACTCGTCAGCGAAGCAGCAAGCTGTTTCGGTCCCCCTCTTTGGTCGTGCAATATTCCCCACTTAACGTCAATGAGCAAAAGCTTGCCAGTATCAGATTCGCACCTGAGCGTCAGGGCCCCCGTCAATTCACTGTCTCACGGTTCCCGAAGGGCCATGATGACTTGAGTCGAGTTGCAGACTTCACCCCAGTCATGAAT |
| st3 | CATCAGGCAGTTTCCCAGACATTACTCACCCGTTATTAGCTACCGTTTCCACGCGGCATGGCTGCATCCTTCCTCCCCGCTGAACACCTGGAATTCTACCCCCGCCTTCGCCACCGGTATCGAATTAAACCACATTCTGAAAACTTCCGTGGGCGCCATTGTAGCACGTCGCACTTTATGAGGTCCCGCAGGTTCCCCTACGG |
| st4 | CTGTTACCGTTCGACTTGCATGTGTTAGGCCTGCATCTGGGCACATCCGAGTCTGGACCGTGTCTCATGGCACGGAGTTAGCCGAGCCCGGGGATTTCACACCAGGGTATCTAATCCTGCGGCCGTACTCCCCAGTCACAACACGAGCTGACTCCTCCAGTTTATCACTTGATCCACGATTACTAGCCCTCCCGAAGGTTAAG |
| ctrl insert | ACCTTCCTTTTCATTTCCCTTCCTTTCCCCCTTTTTTTCCTCTCCTTTCTCCCTTTATTTCTCCTTACCCTTCCTACTTCTCTCTTTCCCTCCCCTCCTCTACTACCCCATCTTTACTCCCCTTTCCTATCCCTCCAACTCACACTAAAC |
| P insert | cgtacggaattcgctagcTAATACGACTCACTATAGGGGAATTGTGAGCGGATAACAATTCCGCCAGCGTTCAATCTGAGCCATGATCAAACTAGGTGAGCCGTTACCCCTCATCCTCTCAGACCAGTAACGCTTGCACCCTCCGCCTGCGTGCGCTTTACCCTCCAAGTCGACATCGGTTAGCTCCGGAAGCCAGGTTGCGCTCGTTGCGGTTCCCGGCCGGACCGCTACAAGGCCCGGGAACGTCAACCCACTCCCATGGTGTGACGGGCTAGCATAACCCCTTGGGGCCTCTAAACGGGTCTTGAGGGGTTTTTTGttttttttACCTTCCTTTTCATTTCCCTTCCTTTCCCCCTTTTTTTCCTCTCCTTTCTCCCTTTATTTCTCCTTACCCTTCCTACTTCTCTCTTTCCCTCCCCTCCTCTACTACCCCATCTTTACTCCCCTTTCCTATCCCTCCAACTCACACTAAACTAATACGACTCACTATAGGGGAATTGTGAGCGGATAACAATTCCGCCACTCGTCAGCGAAGCAGCAAGCTGTTTCGGTCCCCCTCTTTGGTCGTGCAATATTCCCCACTTAACGTCAATGAGCAAAAGCTTGCCAGTATCAGATTCGCACCTGAGCGTCAGGGCCCCCGTCAATTCACTGTCTCACGGTTCCCGAAGGGCCATGATGACTTGAGTCGAGTTGCAGACTTCACCCCAGTCATGAATCTAGCATAACCCCTTGGGGCCTCTAAACGGGTCTTGAGGGGTTTTTTGttttttttTCTTCACTTTAACTCACACTACCCCACATCCCCCTCCTACTCAAAAACTCCCCTCCCACAACCCAAACCCACCACACCAACAACAACCAAAACTCACCCACCCCCCCTCACTAACCCAAAAAAAAAACACCACCAACCAACCATAACTACTAATACGACTCACTATAGGGGAATTGTGAGCGGATAACAATTCATCAGGCAGTTTCCCAGACATTACTCACCCGTTATTAGCTACCGTTTCCACGCGGCATGGCTGCATCCTTCCTCCCCGCTGAACACCTGGAATTCTACCCCCGCCTTCGCCACCGGTATCGAATTAAACCACATTCTGAAAACTTCCGTGGGCGCCATTGTAGCACGTCGCACTTTATGAGGTCCCGCAGGTTCCCCTACGGCTAGCATAACCCCTTGGGGCCTCTAAACGGGTCTTGAGGGGTTTTTTGttttttttTCAATTACCTCTCTCTCATCTCACCTCCTACACCACACCCTCCCAACTAACCACACACCAACACACTCAAACTTCTCTCTATCCCTCCCAATCACCCTACTCCGCGCCTTCCCCTTCCCTTCAGTTTCCCCTTTTTCTCTCTTTCTTCACTAATACGACTCACTATAGGGGAATTGTGAGCGGATAACAATTCTGTTACCGTTCGACTTGCATGTGTTAGGCCTGCATCTGGGCACATCCGAGTCTGGACCGTGTCTCATGGCACGGAGTTAGCCGAGCCCGGGGATTTCACACCAGGGTATCTAATCCTGCGGCCGTACTCCCCAGTCACAACACGAGCTGACTCCTCCAGTTTATCACTTGATCCACGATTACTAGCCCTCCCGAAGGTTAAGCTAGCATAACCCCTTGGGGCCTCTAAACGGGTCTTGAGGGGTTTTTTGggatccgagctccacgtg |
